# Supplementary material for: Poisoning cases and their management in Amhara National Regional State, Ethiopia: Hospital-based prospective study
Source: PLoS One. 2024 May 31;19(5):e0303438. doi: 10.1371/journal.pone.0303438 (PMC11142576; doi:10.1371/journal.pone.0303438)
Supplement: S1 File — The distribution of patient outcomes in the categorical variables, which are not included in the multivariate analysis model. Besides, it provides the details of the patien outcomes with the continuous variables that appeared in the multivariate analysis model. (DOCX) [file pone.0303438.s001.docx]

**Poisoning cases and their management in Amhara National Regional State, Ethiopia: hospital-based prospective study**

**Supporting Information**

This supporting information gives the distribution of patient outcomes in the categorical variables, which are not included in the multivariate analysis model. Besides, it provides the details of the continuous variables that appeared in the multivariate analysis model.

**S1 Table:** Distribution of patient outcome with categorical variables.

| Variables | | Outcome | | Total |
| --- | --- | --- | --- | --- |
|  |  | Cure | Death |  |
| Sex | Male | 137 | 26 | 163 |
|  | Female | 227 | 52 | 279 |
| Religion | Orthodox Christian | 296 | 67 | 363 |
|  | Muslim | 67 | 10 | 77 |
|  | Protestant | 1 | 1 | 2 |
| Marital status | Married | 154 | 47 | 201 |
|  | Single | 199 | 24 | 223 |
|  | Other | 11 | 7 | 18 |
| Educational status | Cannot read and write | 91 | 37 | 128 |
|  | Can read and write | 63 | 9 | 72 |
|  | Primary school | 59 | 8 | 67 |
|  | Secondary school | 100 | 16 | 116 |
|  | College level | 35 | 8 | 43 |
|  | University level | 16 | 0 | 16 |
| Occupation | Student | 124 | 15 | 139 |
|  | Farmer | 93 | 37 | 130 |
|  | Self employed | 79 | 12 | 91 |
|  | House wife | 23 | 3 | 26 |
|  | Government employee | 14 | 6 | 20 |
|  | Daily laborer | 15 | 3 | 18 |
|  | Other | 16 | 2 | 18 |

S1 Table presents data detailing the distribution of treatment outcomes, specifically focusing on categorical variables that were excluded from the multivariate analysis. In essence, the table serves as a supplemental resource providing additional context on treatment outcomes based on categorical variables that were not part of the main multivariate analysis, offering a more comprehensive view of the data and potentially enabling further insights or comparisons.

**S2 Table:** The source of poisoning agents encountered.

| Source | Frequency | Percent |
| --- | --- | --- |
| Home | 243 | 55.0 |
| Shop | 159 | 36.0 |
| Hotel/cafeteria | 24 | 5.4 |
| Other | 16 | 3.6 |
| Total | 442 | 100.0 |

S2 Table offers thorough insights into the various sources of poisoning agents, complementing the data presented in Fig. 1 within the main article. While the figure exclusively displays percentage distributions, this table includes both frequencies and percentages, providing a more comprehensive perspective.

**S3 Table:** Age and patient outcome.

| **Age (years)** | **Outcome** | | **Total** |
| --- | --- | --- | --- |
|  | **Cure** | **Death** |  |
| 1 | 1 | 0 | 1 |
| 4 | 1 | 0 | 1 |
| 14 | 1 | 0 | 1 |
| 15 | 9 | 3 | 12 |
| 16 | 14 | 1 | 15 |
| 17 | 13 | 1 | 14 |
| 18 | 39 | 4 | 43 |
| 19 | 17 | 1 | 18 |
| 20 | 35 | 7 | 42 |
| 21 | 13 | 0 | 13 |
| 22 | 18 | 7 | 25 |
| 23 | 12 | 3 | 15 |
| 24 | 14 | 2 | 16 |
| 25 | 35 | 9 | 44 |
| 26 | 11 | 3 | 14 |
| 27 | 6 | 1 | 7 |
| 28 | 16 | 3 | 19 |
| 29 | 4 | 0 | 4 |
| 30 | 15 | 2 | 17 |
| 31 | 3 | 0 | 3 |
| 32 | 2 | 1 | 3 |
| 33 | 1 | 0 | 1 |
| 35 | 15 | 3 | 18 |
| 36 | 3 | 0 | 3 |
| 37 | 2 | 0 | 2 |
| 38 | 5 | 4 | 9 |
| 40 | 17 | 5 | 22 |
| 41 | 3 | 0 | 3 |
| 42 | 6 | 0 | 6 |
| 43 | 1 | 0 | 1 |
| 45 | 5 | 6 | 11 |
| 46 | 3 | 0 | 3 |
| 48 | 2 | 0 | 2 |
| 50 | 4 | 4 | 8 |
| 52 | 1 | 0 | 1 |
| 55 | 0 | 4 | 4 |
| 59 | 1 | 0 | 1 |
| 60 | 8 | 1 | 9 |
| 62 | 3 | 0 | 3 |
| 63 | 0 | 1 | 1 |
| 65 | 1 | 0 | 1 |
| 68 | 1 | 0 | 1 |
| 70 | 2 | 1 | 3 |
| 75 | 1 | 1 | 2 |
| Total | 364 | 78 | 442 |

S3 Table contains patient outcomes and age, providing additional information into Table 5 of the article, which includes age in the multivariate analysis of treatment outcomes. S3 Table shows the details of age values versus patient outcomes.

**S4 Table:** The time interval between exposure to the poison and presentation to the hospital and patient outcome.

| **Time (hr)** | **Patient outcome** | | **Total** |
| --- | --- | --- | --- |
|  | **Cured** | **Died** |  |
| 0.25 | 4 | 0 | 4 |
| 0.33 | 10 | 1 | 11 |
| 0.50 | 32 | 2 | 34 |
| 0.58 | 2 | 0 | 2 |
| 0.67 | 5 | 1 | 6 |
| 0.75 | 5 | 3 | 8 |
| 0.83 | 7 | 0 | 7 |
| 1.00 | 41 | 5 | 46 |
| 1.17 | 3 | 1 | 4 |
| 1.33 | 2 | 2 | 4 |
| 1.42 | 1 | 0 | 1 |
| 1.50 | 7 | 1 | 8 |
| 1.67 | 2 | 0 | 2 |
| 1.75 | 3 | 1 | 4 |
| 1.83 | 3 | 0 | 3 |
| 2.00 | 33 | 5 | 38 |
| 2.17 | 0 | 2 | 2 |
| 2.25 | 0 | 1 | 1 |
| 2.33 | 3 | 0 | 3 |
| 2.50 | 5 | 3 | 8 |
| 2.75 | 1 | 0 | 1 |
| 3.00 | 29 | 6 | 35 |
| 3.33 | 1 | 0 | 1 |
| 3.40 | 1 | 0 | 1 |
| 3.50 | 4 | 0 | 4 |
| 3.55 | 0 | 1 | 1 |
| 3.58 | 1 | 0 | 1 |
| 3.67 | 1 | 0 | 1 |
| 4.00 | 33 | 9 | 42 |
| 4.25 | 1 | 0 | 1 |
| 4.33 | 2 | 1 | 3 |
| 4.50 | 2 | 1 | 3 |
| 4.58 | 1 | 0 | 1 |
| 4.67 | 1 | 0 | 1 |
| 4.83 | 0 | 1 | 1 |
| 4.92 | 1 | 1 | 2 |
| 5.00 | 20 | 6 | 26 |
| 5.33 | 1 | 0 | 1 |
| 5.38 | 1 | 0 | 1 |
| 5.50 | 2 | 2 | 4 |
| 5.75 | 1 | 0 | 1 |
| 6.00 | 14 | 6 | 20 |
| 6.25 | 0 | 1 | 1 |
| 6.33 | 1 | 0 | 1 |
| 6.50 | 2 | 0 | 2 |
| 6.67 | 0 | 1 | 1 |
| 7.00 | 9 | 1 | 10 |
| 7.50 | 1 | 1 | 2 |
| 7.83 | 1 | 0 | 1 |
| 8.00 | 9 | 3 | 12 |
| 8.83 | 1 | 0 | 1 |
| 9.00 | 5 | 2 | 7 |
| 10.00 | 8 | 1 | 9 |
| 10.67 | 1 | 0 | 1 |
| 11.00 | 5 | 1 | 6 |
| 12.00 | 3 | 2 | 5 |
| 12.50 | 2 | 0 | 2 |
| 13.00 | 2 | 1 | 3 |
| 14.00 | 4 | 0 | 4 |
| 15.00 | 3 | 0 | 3 |
| 16.00 | 1 | 0 | 1 |
| 16.50 | 1 | 0 | 1 |
| 17.00 | 1 | 0 | 1 |
| 17.17 | 1 | 0 | 1 |
| 18.00 | 3 | 0 | 3 |
| 19.00 | 1 | 0 | 1 |
| 24.00 | 4 | 1 | 5 |
| 28.00 | 0 | 1 | 1 |
| 32.00 | 2 | 0 | 2 |
| 40.00 | 1 | 0 | 1 |
| 48.00 | 2 | 0 | 2 |
| 51.00 | 1 | 0 | 1 |
| 72.00 | 1 | 0 | 1 |
| 96.00 | 1 | 0 | 1 |
| 120.00 | 1 | 0 | 1 |

S4 Table provides more information in Table 5 of the article regarding the time of arrival to the hospital, one of the variables included in the multivariate analysis of treatment outcomes. It details the time elapsed from poisoning incidence to presentation to the hospital versus patient outcome.

**S5 Table:** Duration of hospitalization and treatment outcome.

| **Duration of hospitalization (hr)** | **Patient outcome** | | **Total** |
| --- | --- | --- | --- |
|  | **Cured** | **Died** |  |
| 0.33 | 0 | 1 | 1 |
| 0.50 | 0 | 1 | 1 |
| 0.58 | 1 | 0 | 1 |
| 0.67 | 0 | 2 | 2 |
| 1.00 | 1 | 6 | 7 |
| 2.00 | 9 | 7 | 16 |
| 2.50 | 0 | 1 | 1 |
| 3.00 | 2 | 3 | 5 |
| 4.00 | 12 | 4 | 16 |
| 4.50 | 3 | 0 | 3 |
| 5.00 | 3 | 2 | 5 |
| 6.00 | 21 | 8 | 29 |
| 7.00 | 1 | 0 | 1 |
| 8.00 | 13 | 0 | 13 |
| 9.00 | 0 | 3 | 3 |
| 10.00 | 4 | 1 | 5 |
| 10.50 | 1 | 0 | 1 |
| 11.00 | 2 | 1 | 3 |
| 11.33 | 0 | 1 | 1 |
| 11.50 | 1 | 0 | 1 |
| 12.00 | 24 | 4 | 28 |
| 13.00 | 2 | 2 | 4 |
| 14.00 | 1 | 3 | 4 |
| 15.00 | 3 | 1 | 4 |
| 16.00 | 2 | 1 | 3 |
| 17.00 | 2 | 0 | 2 |
| 18.00 | 4 | 1 | 5 |
| 19.00 | 4 | 0 | 4 |
| 20.00 | 4 | 1 | 5 |
| 21.00 | 1 | 0 | 1 |
| 23.00 | 5 | 0 | 5 |
| 24.00 | 71 | 14 | 85 |
| 25.00 | 1 | 1 | 2 |
| 25.50 | 1 | 0 | 1 |
| 26.00 | 5 | 0 | 5 |
| 27.00 | 1 | 0 | 1 |
| 29.00 | 1 | 0 | 1 |
| 30.00 | 3 | 0 | 3 |
| 31.00 | 1 | 0 | 1 |
| 33.00 | 2 | 1 | 3 |
| 36.00 | 3 | 2 | 5 |
| 37.00 | 2 | 0 | 2 |
| 38.00 | 2 | 0 | 2 |
| 40.00 | 2 | 0 | 2 |
| 42.00 | 2 | 0 | 2 |
| 43.00 | 2 | 0 | 2 |
| 44.00 | 2 | 0 | 2 |
| 45.00 | 1 | 0 | 1 |
| 46.00 | 2 | 0 | 2 |
| 48.00 | 52 | 5 | 57 |
| 49.00 | 3 | 0 | 3 |
| 50.00 | 1 | 0 | 1 |
| 52.00 | 1 | 0 | 1 |
| 58.00 | 1 | 0 | 1 |
| 60.00 | 2 | 0 | 2 |
| 68.00 | 1 | 0 | 1 |
| 69.00 | 1 | 0 | 1 |
| 70.00 | 1 | 0 | 1 |
| 71.00 | 2 | 0 | 2 |
| 72.00 | 34 | 1 | 35 |
| 96.00 | 16 | 0 | 16 |
| 99.00 | 1 | 0 | 1 |
| 120.00 | 7 | 0 | 7 |
| 124.00 | 1 | 0 | 1 |
| 144.00 | 4 | 0 | 4 |
| 168.00 | 4 | 0 | 4 |
| 192.00 | 1 | 0 | 1 |
| 216.00 | 1 | 0 | 1 |

S5 Table provides details in Table 5 of the article regarding the length of hospitalization as one of the predictor variables of treatment outcome. It details the duration of hospitalization versus patient outcome. Table 5 provides a general correlation between the duration of hospitalization and patient outcomes, while this table provides specific details on duration of hospitalization versus patient outcomes.

**S6 Table:** Distance from the place of poisoning incident to the hospital.

| **Distance (km)** | **Patient outcome** | | **Total** |
| --- | --- | --- | --- |
|  | **Cured** | **Died** |  |
| 0.10 | 1 | 0 | 1 |
| 0.15 | 1 | 0 | 1 |
| 0.50 | 1 | 0 | 1 |
| 1.00 | 19 | 4 | 23 |
| 1.50 | 1 | 1 | 2 |
| 1.80 | 2 | 0 | 2 |
| 2.00 | 47 | 1 | 48 |
| 2.50 | 5 | 0 | 5 |
| 2.80 | 2 | 0 | 2 |
| 3.00 | 27 | 3 | 30 |
| 4.00 | 16 | 1 | 17 |
| 5.00 | 33 | 3 | 36 |
| 6.00 | 3 | 0 | 3 |
| 7.00 | 5 | 3 | 8 |
| 8.00 | 4 | 0 | 4 |
| 9.00 | 0 | 1 | 1 |
| 10.00 | 8 | 1 | 9 |
| 12.00 | 1 | 5 | 6 |
| 13.00 | 1 | 0 | 1 |
| 15.00 | 6 | 0 | 6 |
| 16.00 | 2 | 0 | 2 |
| 17.00 | 11 | 1 | 12 |
| 18.00 | 5 | 2 | 7 |
| 19.00 | 2 | 0 | 2 |
| 20.00 | 10 | 5 | 15 |
| 21.00 | 2 | 0 | 2 |
| 22.00 | 5 | 2 | 7 |
| 23.00 | 1 | 0 | 1 |
| 24.00 | 3 | 1 | 4 |
| 25.00 | 7 | 1 | 8 |
| 26.00 | 2 | 0 | 2 |
| 27.00 | 11 | 1 | 12 |
| 28.00 | 3 | 0 | 3 |
| 30.00 | 20 | 9 | 29 |
| 31.00 | 3 | 0 | 3 |
| 32.00 | 3 | 0 | 3 |
| 34.00 | 2 | 0 | 2 |
| 35.00 | 6 | 2 | 8 |
| 36.00 | 1 | 1 | 2 |
| 37.00 | 2 | 1 | 3 |
| 38.00 | 1 | 0 | 1 |
| 40.00 | 11 | 3 | 14 |
| 41.00 | 1 | 0 | 1 |
| 42.00 | 11 | 3 | 14 |
| 43.00 | 1 | 0 | 1 |
| 44.00 | 1 | 0 | 1 |
| 45.00 | 3 | 2 | 5 |
| 46.00 | 1 | 0 | 1 |
| 47.00 | 1 | 0 | 1 |
| 48.00 | 0 | 1 | 1 |
| 50.00 | 4 | 5 | 9 |
| 55.00 | 2 | 0 | 2 |
| 57.00 | 0 | 1 | 1 |
| 60.00 | 11 | 3 | 14 |
| 64.00 | 0 | 1 | 1 |
| 65.00 | 2 | 1 | 3 |
| 68.00 | 1 | 1 | 2 |
| 70.00 | 4 | 1 | 5 |
| 72.00 | 1 | 0 | 1 |
| 73.00 | 1 | 0 | 1 |
| 74.00 | 1 | 0 | 1 |
| 78.00 | 1 | 0 | 1 |
| 79.00 | 1 | 0 | 1 |
| 80.00 | 4 | 1 | 5 |
| 82.00 | 2 | 0 | 2 |
| 85.00 | 1 | 0 | 1 |
| 100.00 | 1 | 1 | 2 |
| 102.00 | 2 | 0 | 2 |
| 110.00 | 1 | 0 | 1 |
| 120.00 | 0 | 3 | 3 |
| 130.00 | 1 | 0 | 1 |
| 140.00 | 0 | 1 | 1 |
| 148.00 | 1 | 0 | 1 |
| 168.00 | 0 | 1 | 1 |
| 180.00 | 1 | 0 | 1 |
| 200.00 | 4 | 0 | 4 |
| 418.00 | 1 | 0 | 1 |
| 430.00 | 1 | 0 | 1 |
| Total | 364 | 78 | 442 |

S6 Table gives supplementary information regarding distance from the place of poisoning incident to the hospital attended in Table 5 of the article. In Table 5 of the article, distance has been included in the multivariate analysis of the associated factors with patient outcome. The values in this aspect are presented in compressed manner. This supplementary table provides specific details on distance versus patient outcomes.
