# Supplementary material for: Poisoning cases and their management in Amhara National Regional State, Ethiopia: Hospital-based prospective study
Source: PLoS One. 2024 May 31;19(5):e0303438. doi: 10.1371/journal.pone.0303438 (PMC11142576; doi:10.1371/journal.pone.0303438)
Supplement: Attachment — Submitted filename: PONE-D-22-26894.pdf [file pone.0303438.s002.pdf]

PLOS ONE

Poisoning Cases and Their Management in Amhara Region, Ethiopia: Hospital Based Prospective Study

--Manuscript Draft--

**Manuscript Number:**	PONE-D-22-26894
**Article Type:**	Research Article
**Full Title:**	Poisoning Cases and Their Management in Amhara Region, Ethiopia: Hospital Based Prospective Study
**Short Title:**	Poisoning cases and Their Management
**Corresponding Author:**	Assefa Belay Asrie, MSc<br>University of Gondar<br>Gondar, Amhara National Regional State ETHIOPIA
**Keywords:**	Poisoning, poisoning agents, organophosphate compounds, metal phosphides, poisoning management, treatment outcome
**Abstract:**	Background: Poisoning is a significant public health problem globally and cases are being increased from time to time. As the advances in technological and social developments have led to ease accessibility of most drugs and chemical substances in the society, the number of incidences of poisoning can be higher. Ethiopia is one of the low-income countries that share its considerable burden of poisoning incidences and deaths.<br>Methods: An institution based prospective observational study was employed, as a study design, in selected hospitals of the region.<br>Results: Various poisoning agents involved were identified in this study. Organophosphate compounds, 145 (32.81%), and metal phosphides (majorly aluminum phosphide), 115 (26.02%), were identified to be the first and the send most common causes of poisoning. Most of the cases, 243 (55.00%), accessed the poisoning agents at home followed by at local shops, 159 (35.97%). Almost all poisoning cases are intentional poisonings of attempted suicide. The most frequently reported reasons for self-poisoning were family dispute, 205 (46.38%) and psychiatric disorders, 58 (13.12%). Both pharmacologic and nonpharmacologic methods of treatment were used in the treatment of poisoning cases. The most frequently used pharmacologic agent was cimetidine, 267 (60.41%), followed by atropine, 99 (22.40%), magnesium sulphate, 78 (17.65%), and others, whereas IV maintenance fluids, 239 (54.07%), and gastric lavage, 75 (16.97%), where the first and the second most frequently utilized nonpharmacologic agents/approaches, respectively. More than half of the cases, 265 (59.95%), were hospitalized for shorter or 24 hours periods and 15.38% of the death records were among these section of the cases. Of the total cases, 127 (28.73%) were unconscious up on arrival to the emergency department of the hospitals. From the total, 78 (17.65%) of the cases were died and 40 (9.05%) of them were among those who were unconscious on arrival. The majority (9.75%) of fatal cases were among metal phosphide intoxicated patients followed by in organophosphate compound poisoned cases (5.66%). From multivariate logistic, age, altered level of consciousness on hospital arrival, distance from the area where the poisoning incidence happened to the hospital they attended, and no laboratory result as part of diagnosis process or investigation of extent of toxicity were found to be significantly associated with likelihood of poor treatment outcome or death.<br>Conclusion: Organophosphate compounds and metal phosphides were the first and second most frequently encountered poisoning agents, respectively, and it was noted that the later one was responsible for most of the fatal cases. The fatality rate of poisoning in this study was found to be much higher than in other similar studies.
**Order of Authors:**	Assefa Belay Asrie, MSc
	Kefyalew Ayalew Getahun, MSc
	Eshetie Melese Birru, MSc
	Gashaw Binega Mekonnen, MSc
	Geta Asrade Alemayehu, MPH

Powered by Editorial Manager® and ProduXion Manager® from Aries Systems Corporation

	Berhanu Fikadie Endehabtu, MPH
	Marta Berta, PhD Candidate
	Getinet Mequanint Adinew, MSc
**Additional Information:**	
**Question**	**Response**
**Financial Disclosure**<br><br>Enter a financial disclosure statement that describes the sources of funding for the work included in this submission. Review the submission guidelines for detailed requirements. View published research articles from *PLOS ONE* for specific examples.<br><br>This statement is required for submission and **will appear in the published article** if the submission is accepted. Please make sure it is accurate.<br><br>**Unfunded studies**<br>Enter: *The author(s) received no specific funding for this work.*<br><br>**Funded studies**<br>Enter a statement with the following details:<br>• Initials of the authors who received each award<br>• Grant numbers awarded to each author<br>• The full name of each funder<br>• URL of each funder website<br>• Did the sponsors or funders play any role in the study design, data collection and analysis, decision to publish, or preparation of the manuscript?<br>• **NO** - Include this sentence at the end of your statement: *The funders had no role in study design, data collection and analysis, decision to publish, or preparation of the manuscript.*<br>• **YES** - Specify the role(s) played.<br><br>* typeset	This study was done using a grant awarded by University of Gondar, Ethiopia, Reference № R/T/T/C/Eng.58/03-2018
**Competing Interests**<br><br>Use the instructions below to enter a competing interest statement for this submission. On behalf of all authors, disclose any competing interests that could be perceived to bias this	We have no competing interests regarding this work

work—acknowledging all financial support and any other relevant financial or non-financial competing interests.<br><br>This statement is required for submission and **will appear in the published article** if the submission is accepted. Please make sure it is accurate and that any funding sources listed in your Funding Information later in the submission form are also declared in your Financial Disclosure statement.<br><br>View published research articles from *PLOS ONE* for specific examples.<br><br>**NO authors have competing interests**<br><br>Enter: *The authors have declared that no competing interests exist.*<br><br>**Authors with competing interests**<br><br>Enter competing interest details beginning with this statement:<br><br>*I have read the journal's policy and the authors of this manuscript have the following competing interests: [insert competing interests here]*<br><br>* typeset	
**Ethics Statement**<br><br>Enter an ethics statement for this submission. This statement is required if the study involved:<br><br>• Human participants<br>• Human specimens or tissue<br>• Vertebrate animals or cephalopods<br>• Vertebrate embryos or tissues<br>• Field research<br><br>Write "N/A" if the submission does not require an ethics statement.	The protocol of the study was reviewed and approved by University of Gondar, College of Medicine and Health Sciences, Ethical Review Committee. In addition, each hospital administration office was requested by formal letter from the University of Gondar, College of Medicine and Health Sciences, Research and Community Service Office and permission was obtained from each hospital to conduct the study. Furthermore, participant informed consent to participate in the study was obtained from each patient and/or care giver before the data collection. Those who were unwilling to participate were excluded. No personal identifier was included in the data collection tool and the data record was kept confidential and used only for the study purpose.

General guidance is provided below. Consult the submission guidelines for detailed instructions. **Make sure that all information entered here is included in the Methods section of the manuscript.**<br><br>**Format for specific study types**<br><br>**Human Subject Research (involving human participants and/or tissue)**<br>• Give the name of the institutional review board or ethics committee that approved the study<br>• Include the approval number and/or a statement indicating approval of this research<br>• Indicate the form of consent obtained (written/oral) or the reason that consent was not obtained (e.g. the data were analyzed anonymously)<br><br>**Animal Research (involving vertebrate animals, embryos or tissues)**<br>• Provide the name of the Institutional Animal Care and Use Committee (IACUC) or other relevant ethics board that reviewed the study protocol, and indicate whether they approved this research or granted a formal waiver of ethical approval<br>• Include an approval number if one was obtained<br>• If the study involved *non-human primates*, add *additional details* about animal welfare and steps taken to ameliorate suffering<br>• If anesthesia, euthanasia, or any kind of animal sacrifice is part of the study, include briefly which substances and/or methods were applied<br><br>**Field Research**<br><br>Include the following details if this study involves the collection of plant, animal, or other materials from a natural setting:<br>• Field permit number<br>• Name of the institution or relevant body that granted permission	
**Data Availability**<br><br>Authors are required to make all data underlying the findings described fully	Yes - all data are fully available without restriction

Powered by Editorial Manager® and ProduXion Manager® from Aries Systems Corporation

available, without restriction, and from the time of publication. PLOS allows rare exceptions to address legal and ethical concerns. See the PLOS Data Policy and FAQ for detailed information.<br><br>A Data Availability Statement describing where the data can be found is required at submission. Your answers to this question constitute the Data Availability Statement and **will be published in the article**, if accepted.<br><br>**Important:** Stating 'data available on request from the author' is not sufficient. If your data are only available upon request, select 'No' for the first question and explain your exceptional situation in the text box.<br><br>Do the authors confirm that all data underlying the findings described in their manuscript are fully available without restriction?	
**Describe where the data may be found in full sentences. If you are copying our sample text, replace any instances of XXX with the appropriate details.**<br><br>• If the data are **held or will be held in a public repository**, include URLs, accession numbers or DOIs. If this information will only be available after acceptance, indicate this by ticking the box below. For example: *All XXX files are available from the XXX database (accession number(s) XXX, XXX.*).<br>• If the data are all contained **within the manuscript and/or Supporting Information files**, enter the following: *All relevant data are within the manuscript and its Supporting Information files.*<br>• If neither of these applies but you are able to provide **details of access elsewhere**, with or without limitations, please do so. For example:<br><br>*Data cannot be shared publicly because of [XXX]. Data are available from the XXX Institutional Data Access / Ethics*	The datasets of the study can be obtained from the corresponding author on reasonable request.

Committee (contact via XXX) for researchers who meet the criteria for access to confidential data.<br><br>*The data underlying the results presented in the study are available from (include the name of the third party and contact information or URL).*<br>• This text is appropriate if the data are owned by a third party and authors do not have permission to share the data.<br><br>* typeset	
Additional data availability information:	Tick here if the URLs/accession numbers/DOIs will be available only after acceptance of the manuscript for publication so that we can ensure their inclusion before publication.

Powered by Editorial Manager® and ProduXion Manager® from Aries Systems Corporation

Manuscript

Click here to access/download;Manuscript;Poisoning Manuscript for PLOS.docx

Poisoning Cases and Their Management in Amhara Region, Ethiopia: Hospital Based Prospective Study

Assefa Belay Asrie[1], Seyfe Asrade Atnafie[1], Kefyalew Ayalew Getahun[1], Eshetie Melese Birru[1], Gashaw Binega Mekonnen[2], Geta Asrade Alemayehu[3], Berhanu Fikadie Endehabtu[4], Marta Berta[5], Getinet Mequanint Adinew[1]

[1]Department of Pharmacology, School of Pharmacy, College of Medicine and Health Sciences, University of Gondar, Gondar, Ethiopia

[2]Department of Clinical Pharmacy, School of Pharmacy, College of Medicine and Health Sciences, University of Gondar, Gondar, Ethiopia

[3]Department of Health Service Management and Health Economics, Institute of Public Health, College of Medicine and Health Sciences, University of Gondar, Ethiopia

[4]Department of Health Informatics, Institute of Public Health, University of Gondar, Gondar, Ethiopia

[5]Department of Midwifery, College of Medicine and Health Sciences, University of Gondar, Gondar, Ethiopia

Abstract

Background: Poisoning is a significant public health problem globally and cases are being increased from time to time. As the advances in technological and social developments have led to ease accessibility of most drugs and chemical substances in the society, the number of incidences of poisoning can be higher. Ethiopia is one of the low-income countries that share its considerable burden of poisoning incidences and deaths.

Methods: An institution based prospective observational study was employed, as a study design, in selected hospitals of the region.

Results: Various poisoning agents involved were identified in this study. Organophosphate compounds, 145 (32.81%), and metal phosphides (majorly aluminum phosphide), 115 (26.02%), were identified to be the first and the se[illegible]nd most common causes of poisoning. Most of the cases, 243 (55.00%), accessed the poisoning agents at home followed by at local shops, 159 (35.97%). Almost all poisoning cases are intentional poisonings of attempted suicide. The most frequently

reported reasons for self-poisoning were family dispute, 205 (46.38%) and psychiatric disorders, 58 (13.12%). Both pharmacologic and nonpharmacologic methods of treatment were used in the treatment of poisoning cases. The most frequently used pharmacologic agent was cimetidine, 267 (60.41%), followed by atropine, 99 (22.40%), magnesium sulphate, 78 (17.65%), and others, whereas IV maintenance fluids, 239 (54.07%), and gastric lavage, 75 (16.97%), where the first and the second most frequently utilized nonpharmacologic agents/approaches, respectively. More than half of the cases, 265 (59.95%), were hospitalized for shorter or 24 hours periods and 15.38% of the death records were among these section of the cases. Of the total cases, 127 (28.73%) were unconscious up on arrival to the emergency department of the hospitals. From the total, 78 (17.65%) of the cases were died and 40 (9.05%) of them were among those who were unconscious on arrival. The majority (9.75%) of fatal cases were among metal phosphide intoxicated patients followed by in organophosphate compound poisoned cases (5.66%). From multivariate logistic, age, altered level of consciousness on hospital arrival, distance from the area where the poisoning incidence happened to the hospital they attended, and no laboratory result as part of diagnosis process or investigation of extent of toxicity were found to be significantly associated with likelihood of poor treatment outcome or death.

Conclusion: Organophosphate compounds and metal phosphides were the first and second most frequently encountered poisoning agents, respectively, and it was noted that the later one was responsible for most of the fatal cases. The fatality rate of poisoning in this study was found to be much higher than in other similar studies.

Key words: Poisoning, poisoning agents, organophosphate compounds, metal phosphides, poisoning management, treatment outcome

Background

A *poison* is any substance with a potential to cause harm on health. Normally, poisons are considered as substances which are dangerous in small amounts such as cyanide, arsenic and strychnine. However, clinically useful drugs and medications are also poisons if they are consumed in an overdose and even life-sustaining substances including water and oxygen can harm the human body if they are extremely excess in the body (1). Chronic poisonings or poisonings with delayed health effects are often more problematic in the long run (2). If the signs of toxicity manifested short time after exposure to the toxic substance, usually within hours, it is regarded as acute poisoning (3). Poisoning is a common cause for emergency visit and hospital admission worldwide and a major reason for illness and death in many countries (3). The incidence of poisoning could be intentional or unintentional, and in children, because of the desire for imitating adults, the unintentional or accidental poisoning is frequent among them (4).

Poisoning is a significant public health problem globally and cases are being increased from time to time due to life style and social development changes (5). According World Health Organization (WHO), about 193,460 deaths due to unintentional poisoning were reported worldwide and out of them 84% were in low- and middle-income countries (5). As the advances in technological and social developments have led to ease accessibility of most drugs and chemical substances in the society, the actual number of incidences of poisoning can be higher, because most cases of poisoning actually go unreported (6). Ethiopia is one of the low-income countries that share its considerable burden of poisoning incidences and deaths. Poisoning was reported as the second most common means of attempted suicide in a district of Ethiopia. The authors also reported that higher numbers poisoning cases women, and strong detergents and rodenticides were the most frequently used poisons (7). One prospective study done in selected hospitals in western Ethiopia reported that about 7.10% poisoning cases were died from the poisonings (8). Another retrospective study reported that 6.60% of the poisoning cases were died (9). It can be depicted from such dep[illegible]s that poisoning is one of the health problems contributing its own to morbidity and mortality burdens in the country.

The types and frequencies of encountered poisoning agents vary considerably across the world and even in different parts of the same country due to socioeconomic and cultural differences. The frequency of poisoning incidences in a given community depends on

the availability of poisonous agents, the common occupational activity in that society, and religious and sociocultural influences (10-12). Household chemicals and prescribed drugs are the most common poisoning agents in the developed world, while in developing countries; agricultural chemicals are the most common poisoning agents (10). More importantly, limited drug and chemical regulatory activities, inadequacy of surveillance systems, poor enforcement, and easy access to various kinds of drugs or chemicals have been implicated for the higher poisoning prevalence in underdeveloped countries (13, 14).

This study was carried out to determine the frequently encountered poisoning agents, common signs and symptoms of poisoning, the clinical managements and the final outcome as cure or death and associated factors.

Methods

The study area

The study was conducted in four referral hospitals in Amhara Region from January, 2018 to December, 2019. The Amhara Region is one of the 10 Regional States in Ethiopia. There were more than 70 hospitals, 100 health centres and 700 health stations the region during the proposal development for this study. Four referral hospitals were conveniently selected as the study site of this study. The hospitals included in the study are University of Gondar Comprehensive Specialized Hospital, Felege Hiwot Referral Hospital, Debre Markos Referral Hospital and Dessie Referral Hospital which are found in Gondar, Bahir, Dessie, and Debre Markos Towns of Amhara Regional State, respectively.

Study design and sampling technique

An institution based prospective observational study was employed, as a study design, in selected hospitals of the region. Considering the number of referral hospitals and their distribution in the region, four referral hospitals were conveniently selected for the study. All poisoning cases admitted to the emergency departments of the hospitals, during the data collection period, were included in the study regardless of their age and causes of poisoning. The cases that were not willing to participate in the study and from whom adequate data were not obtained had been excluded from the study.

Sample size determination

The sample size was determined by using a single population proportion formula considering the following assumptions: prevalence (p) of poisoning cases 50% to get the larger sample size, standard normal distribution value at 95% confidence level of Z/2 = 1.96, and margin of error (w) = 5%. This gives a sample size of 384 patients as shown below.

$$N = \frac{Z^2 p(1-p)}{w^2} = \frac{(1.96)^2 0.5(1-0.5)}{(0.05)^2} = 384.16 \approx \ 384 \text{ patients}$$

Where, N is the sample size, Z is confidence interval, p is a proportion, and w is the margin of error.

Once the minimum number of sample was obtained in this way, by adding a 15% non-response rate, the total sample size was determined to be 442. This whole sample was distributed for the four hospitals. Accordingly, 110 cases were taken from each University of Gondar Referral Hospital and Felege Hiwot Referral Hospital and 111 cases from Debre Markos Referral Hospital and Dessie Referral Hospital.

The data collection tool, process, and quality control

Data was collected by using a structured questionnaire. The questionnaire was designed to collect data regarding the patients' socio-demographic details (gender, age, ethnicity, occupation, educational status, and marital status) and facts related to the poisoning, such as the type of poison used, reasons for deliberate self-poisoning, the clinical state of the patient on presentation at the emergency department, sign and symptoms of poisoning and the treatment and outcome of the case. In each site, the data were collected by two trained nurses and working in the emergency department of the hospital. The data collection process was by filling the questionnaire on spot during diagnosis by attendant physician and in part by reviewing the patient record chart.

To ensure the quality of the data collected, the data collectors were trained on the overall study design and objectives, the contents of the questionnaire, and approaching interviewees. Moreover, supervisors were assigned from the study team for periodic monitoring of the data collection process. In the supervision, some filled questionnaires were randomly selected and crosschecked with their respective patient medical records. Furthermore, each filled questionnaire was checked for its completeness and clarity before received from the data collectors.

Data entry, analysis, and interpretation

The data was entered in to SPSS Version 20 and analyzed using statistical tests. The interpretations were done using descriptive statistics: percentages and frequency distributions. Furthermore the impacts of potential predictors of treatment outcomes (cure or death) were estimated using binary logistic regression analysis at a cutoff point, $p < 0.05$, for statistically significant association and confidence interval (CI) of 95%. In this regard, multivariate analysis was carried out for predictor variables using backward stepwise multiple logistic regression. Adjusted odds ratio (AOR) and its 95% CI was calculated for each predictor variable. The variables found to be significantly associated with the likelihood of death were considered as the risk factors for poor treatment outcome of poisoning.

Ethical approval and patient consent

The proposal of the study was presented to University of Gondar, College of Medicine and Health Sciences, Ethical Review Committee and ethical clearance was obtained from the committee. In addition, each hospital was requested for permission to conduct the study by formal letter. In addition, the participants were asked for verbal informed consent prior to collection of data and their decision was respected. In addition, the data collection questionnaire had no personal identifiers so that the privacy of each respondent was kept. Furthermore, the data collected from each case was kept confidential and used only for the purpose of the study.

Results

Socio-demographic characteristics of the participants

Of the study participants, 279 (63.12%) patients were females and 163 (36.90%) males. Regarding their age, 147 (33.26%), 235 (53.17%), and 60 (13.58%) of them were ≤20, 20-40, and >60 years of old in order. In religion, the majority, 363 (82.13%), of the participants were Orthodox Christians and the remaining, 77 (17.42%) and 2 (0.50%), were Muslims and Protestants, respectively. Considering residence, 226 (51.13%) of the cases were from the urban and 216 (48.87%) from rural residential areas. In their marital status, 223 (50.45%), 201 (45.48%), and 18 (4.07%) were in order single, married and others including divorced, widowed, and separated. Almost all, 434 (98.20%), were Amhara in their ethnic group. Many of the participants, 128 (28.96%), could not read and write or simply they were illiterates and 116 (26.24%) were at secondary school level. The others, 72 (16.29%) could read and write and 67

(15.16%), 43 (9.73%), and 16 (3.62%) were at primary school, college and university educational level, respectively. In occupational aspect, 139 (31.45%), 130 (29.41%), 91 (20.59%), 26 (5.88%), 20 (4.52%), 18 (4.07%), 18 (4.07%) of the study subjects were students, farmers, self-employees, house wives, government employees, daily laborers and others in order (Table 1).

Table 1 Socio-demographic characteristics of the participants.

**Variables**		**Frequency**	**%**
Sex	Male	163	36.90
	Female	279	63.12
Age	≤20 years	147	33.26
	20-40 years	235	53.17
	>40 years	60	13.57
Regi[illegible]	Orthodox Christian	363	82.13
	Muslim	77	17.42
	Protestant	2	0.50
Ethnicity	Amhara	434	98.20
	Others	8	1.80
Residence	Rural	216	48.87
	Urban	226	51.13
Marital status	Married	201	45.48
	Single	223	50.45
	Other	18	4.07
Educational status	Cannot read and write	128	28.96
	Can read and write	72	16.29
	Primary school	67	15.16
	Secondary school	116	26.24
	College level	43	9.73
	University level	16	3.62
Occupation	Student	139	31.45
	Farmer	130	29.41
	Self-employee[a]	91	20.59
	House wife	26	5.88
	Government employee	20	4.52
	Daily laborer	18	4.07
	Others	18	4.07

Distance from the place of poisoning and time of arrival to the hospital after poisoning

Many of the poisoning cases, 193 (43.67%), came from places ≤10 km far from the hospitals they atten[illegible]he others 51 (11.54%), 68 (15.38%), 36 (8.14%), and 94 (21.27%) were from areas of 10-20, 20-30, 30-40, and >40 km far, respectively. The majority, 260 (58.82%), of the cases were arrived to the hospitals after 2 hours of poisoning incidence (Table 2).

Table 2 Distance from the place of poisoning and time of arrival to the hospital after poisoning.

**Distance from place of poisoning incident to hospital (km)**	**Time of arrival to hospital**		**Total (%)**
	**within 2 hours**	**after 2 hours**	
≤10	142	51	193 (43.67)
10-20	21	30	51(11.54)
20-30	12	56	68 (15.38)
30-40	3	33	36 (8.14)
>40	4	90	94 (21.27)
Total (%)	182 (41.18)	260 (58.82)	442 (100)

Sign and symptoms of poisoning

The patients were with a variety of signs and symptoms. In most instances, each case showed two or more signs and symptoms. The most common, 252 (57.01%), sign and symptom of was vomiting followed by epigastric pain, 137 (31.00%). This may be because of as the most common route of exposure is oral route. Other signs and symptoms reported and/or observed were salivation and/or generalized sweating 63 (14.25%), hypothermia 40 (9.05%), hypotension 38 (8.60%), headache 22 (4.98%), diarrhea 18 (4.07), and others including sniffing, unexplained bad odor, restlessness, weakness and seizure (Table 3).

Table 3 Sign and symptoms of the poisoned patients in Amhara Region, February 2019-March 2020.

**Sign and symptom**	**Frequency***	**%**
Altered level of consciousness	234	52.94
Vomiting	252	57.01
Epigastric pain	137	31.00
Salivation and/or generalize body secretion	63	14.25
Hypothermia[a]	40	9.05
Hypotension through shock[b]	38	8.60
Headache	22	4.98
Diarrhea	18	4.07
Sniffing	15	3.39
Unexplained bad odor	15	3.39
Restlessness	12	2.71
Weakness	12	2.71
Seizure	11	2.49

[a]Out of them, 16 patients were with cold extremities
[b]Out of them, 16 patients were under shock

* Symptoms of frequency 10 and less have been excluded

Types or causes poisoning

Organophosphate compounds, 145 (32.81%), and metal phosphides, 115 (26.02%), were the first and the se[illegible] most common causes of poisoning followed by bleaching agents, 44 (9.95%), medicines, 33 (7.47%), rat poisons, 26 (5.88%), alcohol, 20 (4.52%), carbon monoxide, 14 (3.17%), and food poisoning, 12 (2.71%). Other less common causes of poisoning were unknown chemicals, 7 (1.58%), herbs, 5 (1.13%), snake bite, 5 (1.13%), DDT, 4 (0.90%), and others (Table 4).

Table 4 Types/causes poisoning involved.

**Poisoning agent**	**Frequency**	**%**
Organophosphates	145	32.81
Metal phosphides	115	26.02
Bleaching agents	44	9.95
Medicines	33	7.47
Rat poison	26	5.88
Alcohol	20	4.52
Carbon monoxide	14	3.17
Food poisoning	12	2.71
Unknown chemicals	7	1.58
Herbs	5	1.13
Snake bite	5	1.13
DDT	4	0.90
Others*	12	2.71

*Include fertilizer, unspecified herbicides & insecticides, and match head

Dosage form of the poisons encountered and the routes of exposure

By dosage form, 217 (49.10%) of the poisoning agents were in solid dosage form and almost all were taken through oral route of exposure. The other 204 (46.15%) were regarded as liquid dosage forms of which 198 were consumed orally. This shows that the majority of poising cases were caused by poisons of solid and liquid dosage forms and the commonest route of exposure was the oral route, 414 (93.67%). The liquid dosage forms with other routes of exposure were snake venoms because of five snake bite accidents and one topical exposure of an

organophosphate compound. Twenty (4.52%) of the agents were in gaseous forms and out of them 19 were with inhalational routes of exposure (Table 5).

Table 5 Dosage form of the poisons involved and the route of exposure

Dosage form of the poison	Route of exposure			Total (%)
	Oral	Inhalational	Other	
Solid	216	1	0	217 (49.10%)
Liquid	198	0	6	204 (46.15%)
Gas	0	19	1	20 (4.52)
Other[#]	0	0	1	1 (0.23%)
**Total (%)**	414 (93.67%	20 (4.52%)	8 (1.81%)	442 (100%)

[#]Semisolid herbal preparation of topical exposure

Sources of the poisoning agents

Home was reported by most of the poisoned patients as source of poisoning agents, 243 (55.00%), followed by shops, 159 (35.97%), hotel/cafeteria, 24 (5.43%), and other sources, 16 (3.62%). The other sources reported include pharmacy, veterinary drug vender, work place, fire accident smoke, traditional medical practitioner (Figure 1).

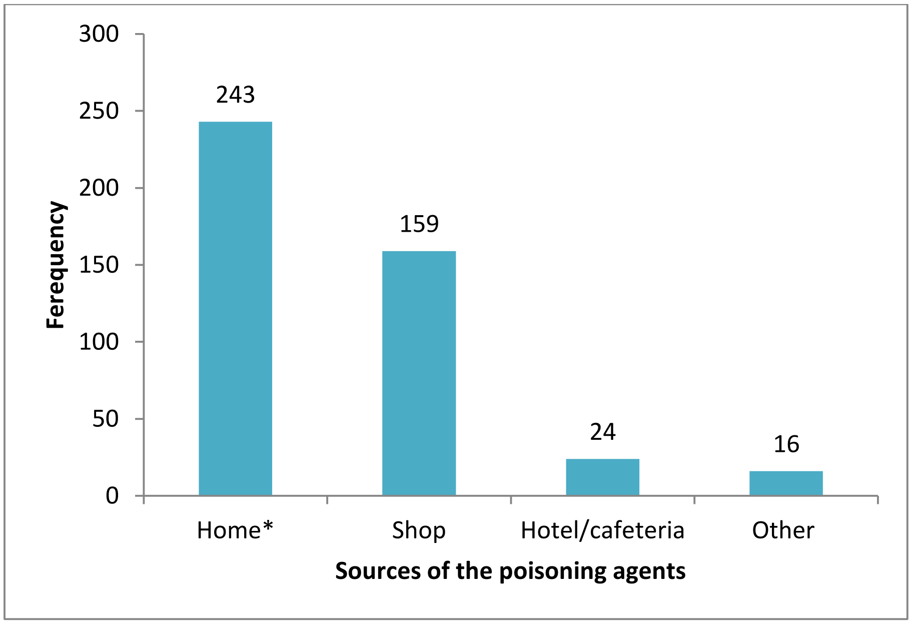

Figure 1 Source of poisons used for poisoning of patients attending the hospital emergency departments. *Includes homes other than they were living in.

Reasons for poisoning incidents

Near to half of the patients, 205 (46.38%), reported family dispute as a reason for intentional poisoning. Other reasons that were reported by the victims/care givers include psychiatric disorder, 58 (13.12%), love affairs, 45 (10.18%), accidental exposure, 32 (7.24%), joblessness, 18 (4.07%), disease treatment, 11 (2.49%), unplanned pregnancy, 8 (1.81%), loss of beloved ones, 7 (1.58%), academic failure, 6 (1.36%) and others, 7 (1.58%). In addition to specified reasons, 34 (7.69%) of the patients took the poisons for unknown reasons (Table 6).

Table 6 Reported reasons behind the poisoning incidents

**Reason**	**Frequency**	**%**
Dispute related[a]	205	46.38
Psychiatric disorder	58	13.12
Love affairs	45	10.18
Unknown	34	7.69
Accident	32	7.24
Joblessness	18	4.07
Disease treatment	11	2.49
Recreational purpose	11	2.49
Unplanned pregnancy	8	1.81
Loss of beloved ones	7	1.58
Academic failure	6	1.36
Other	7	1.58

[a]Most dispute related reasons are disputes within families.

Treatments

Both pharmacologic and nonpharmacologic methods of treatment were used in the treatment of poisoning cases. The most frequently used pharmacologic agent was cimetidine, 267 (60.41%), followed by atropine, 99 (22.40%), magnesium sulphate, 78 (17.65%), antibiotics, 54 (12.22%), hydrocortisone 46 (10.41%), omeprazole 41 (9.28%), frusemide, 28 (6.33%), dopamine, 21 (4.75%), vitamin B complex, 16 (3.62%), calcium gluconate, 10 (2.26%), tramadol, 6 (1.36%) and antacids, 5 (1.13%). Nonpharmacologic agents/approaches used were IV maintenance fluids, 239 (54.07%), gastric lavage, 75 (16.97%), 40% glucose solution, 15 (3.39%) and ORS, 8 (1.81%). Other agents, that were less frequently used, include paracetamol, cathartics, emetics diazepam, heparin, mannitol snake antivenom sera, vitamin K, pantoprazole, and diclofenac. Interestingly, in all cases dialysis was used only for one case, specifically at Felege Hiwot Referral Hospital (Table 7).

Table 7 Treatment given to the poisoned patients

Treatment	Number of patients	%
Cimetidine	267	60.41
Maintenance fluid	239	54.07
Atropine	99	22.40
Magnesium sulphate	78	17.65
Gastric lavage	75	16.97
Antibiotic/s	54	12.22
Hydrocortisone	46	10.41
Omeprazole	41	9.28
Frusemide	28	6.33
Dopamine	21	4.75
Vit. B complex	16	3.62
40% glucose	15	3.39
Calcium gluconate	10	2.26
ORS	8	1.81
Tramadol	6	1.36
Antacids	5	1.13
Others	26	5.88

Hospital stays of the poisoning cases after admission and treatment outcome

The duration of hospital stay of each case was from the time of admission to termination of hospital stay because of discharge following recovery from poisoning symptoms or because of death. Accordingly, 265 (59.95%) of the poisoned patients were hospitalized for ≤24 hours and the remaining, 94 (21.27%), 48 (10.86%), and 35 (7.92%) were hospitalized for 24-48, 48-72, and longer than 72 hours, respectively (Figure 3). The majority of death records, 15.38%, were from the patients with 24 hours or shorter period of hospital stay. In the patients under the category of 24-48 hours duration of stay, 2.04% death was recorded as calculated from the total cases. No fatal case was recorded among those admitted for longer than 72 hours in the hospitals (Figure 2).

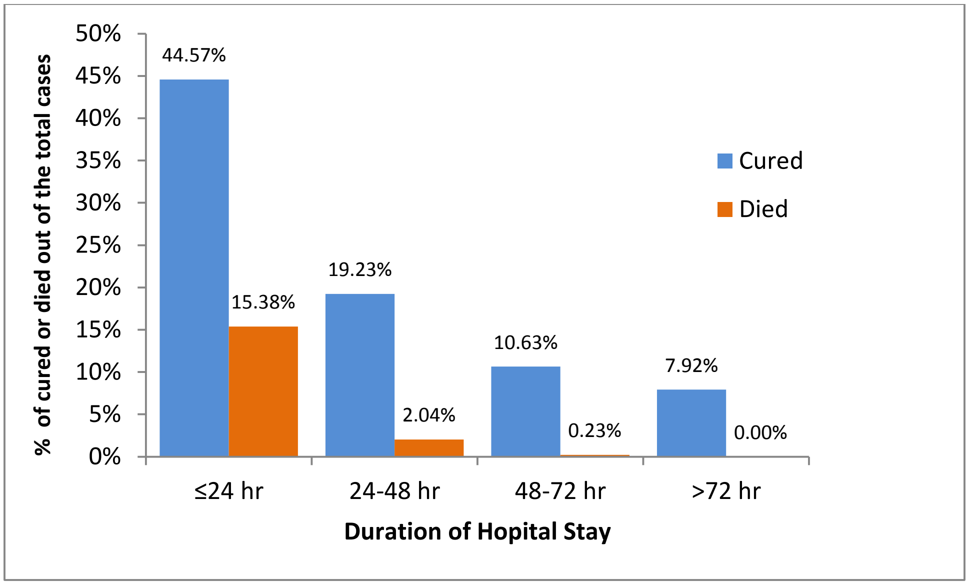

Figure 2 Duration of hospital study and treatment outcome.

The treatment outcome of the poisoning cases by the levels of consciousness

Based on observational and physical assessments up on hospital arrival, the cases were classified as conscious, semiconscious or unconscious by level of consciousness. Accordingly, 208 (47.06%), 107 (24.21%), and 127 (28.73%) of the cases were conscious, semiconscious, and unconscious, respectively, when they arrived at the emergency department of the hospitals. From the total, 78 (17.65%) of the cases were died; and the distributions by the level of consciousness, 19 (4.30%), 19 (4.30%), and 40 (9.05%) were from the cases arrived in conscious, semiconscious and unconscious state, respectively. This result shows that the majority of death record was from the cases arrived at the hospital in unconscious state. Considering percent of cases died within each level of consciousness, higher proportion of individuals were died in the cases arrived in unconscious state, 40 (31.50%), followed by 19 (17.76%) in those arrived in semiconscious state (Figure 3).

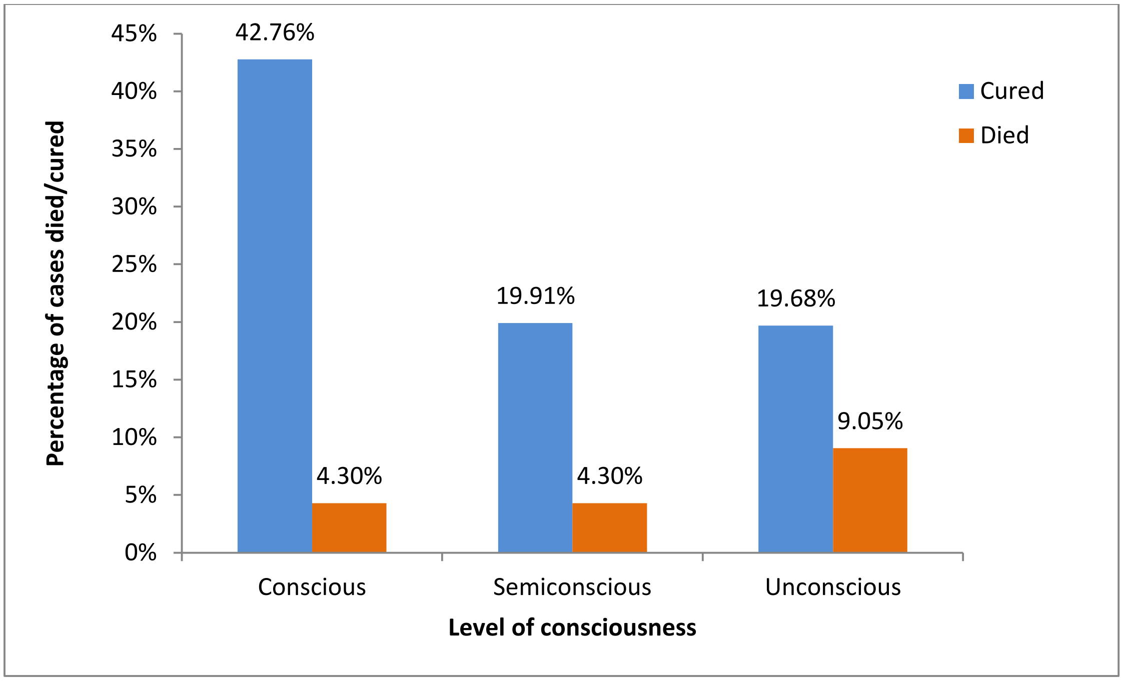

Figure 3 Patient-consciousness upon arrival to the hospital and the treatment outcome.

Distribution of poisoning deaths by the type of poisoning agents

The fatality rate in this study was found to be 17.65%. The majority (9.75%) of fatal cases were among metal phosphide intoxicated patients followed by in organophosphate compound poisoned cases (5.66%). The other agents, drugs, chemicals mentioned as rat poison, and unknown chemicals contributed 0.45%, 0.90%, and 0.23% deaths out of the total cases (Table 8)

Table 8 Distribution of poisoning cases ant treatment outcome by the poisoning agents involved

**Poisoning agents**	**Treatment outcome, n(%)**		**Total, n(%)**
	**Cured**	**Died**	
Organophosphates	120(27.15)	25 (5.66)	145 (32.81)
Metal phosphides	72(16.29)	43(9.75)	115 (26.02)
Bleaching agents	44(9.95)	0(0.00)	44(9.95)
Drugs	31(7.01)	2(0.45)	33(7.47)
Rat poison	22(4.98)	4(0.90)	26(5.88)
Alcohol	20(4.52)	0(0.00)	20(4.52)
Carbon monoxide	14(3.17)	0(0.00)	14(3.17)
Food	12(2.71)	0(0.00)	12(2.71)
Unknown chemicals	6(1.36)	1(0.23)	7(1.58)
Herbal	5(1.13)	0(0.00)	5(1.13)

Snake bite	5(1.13)	0(0.00)	5(1.13)
Organochlorine	4(0.90)	0(0.00)	4(0.90)
Others	9(2.04)	3(0.00)	12(2.71)
Total	364(82.35)	78 (17.65)	442(100.00)

Factors Associated With Treatment Outcome of Poisoning Emergency

First bivariate analysis was done for each potential variable assumed to be associated with treatment outcome and the variables that were verified to significantly associate with treatment outcome were included in multivariate logistic regression model. The analysis showed that the patients aged 20–40 years were 2.106 times more likely to die of poisoning (AOR: 2.106, 95% CI: 1.097–4.043, $p < 0.05$) than those aged 20 years and younger. The patients who were older than 40 years were also 3.630 times more likely to die (AOR: 3.630, 95% CI: 1.561-8.440, $p < 0.01$) than those aged 20 years or younger. Based on the patients' level of consciousness up on arrival at the emergency departments of the hospitals, those that were in semiconscious state were 2.673 times more likely to die (AOR: 2.673, 95% CI: 1.292–5.532, $p < 0.01$) those that were in conscious state. Moreover, those that were in unconscious state at arrival were 5.297 times more likely to die (AOR: 5.297, 95% CI: 2.762-10.160, $p < 0.001$) than those that were in conscious state. Considering the distance from the area where the poisoning incidence happened and the hospital they attended, the odds of the likelihood to die were 3.575 times higher among the poisoned patients who came from areas 10-20 km far than among patients from areas 10 km or nearer (AOR: 3.575, 95% CI: 1.410-9.062, $p < 0.01$). Those who came from areas more than 40 km far were 4.277 times more likely to die (AOR: 4.277, 95 CI: 1.699-10.763, $p <0.001$) than those who came from areas of 10 km or nearer. In addition, the odds of death among poisoned patients who had no laboratory result as part of their diagnosis process or investigation of extent of toxicity was 3.904 times (AOR: 3.904, 95% Cl: 1.880-8.105, $p < 0.001$) than of those whose diagnosis involve laboratory methods (Table 9).

Table 9 Multivariate analysis of factors associated with treatment outcome (R^2=0.230), Hosmer and Lemeshow goodness of Fit Test, p=0.367)

**Explanatory Variables**	**Treatment outcome**		**AOR (95% C.I.)**	**P-value**
	**Cured**	**Died**		
**Age**				0.009
≤20 years	130	17	1	
20-40 years	192	43	**2.106 (1.097-4.043)***	
>40 years	42	18	**3.630 (1.561-8.440)****	
**Residence**				0.526
Rural	169	47	1	

Urban	195	31	1.227 (0.652-2.311)	
**Patient consciousness when arrived to hospital**				0.000
Conscious	189	19	1	
Semiconscious	88	19	**2.673 (1.292-5.532)****	
Unconscious	87	40	**5.297 (2.762-10.160)*****	
**Distance from the place of poisoning to hospital (km)**				0.020
<10	175	18	1	
10-20	38	13	**3.575 (1.410-9.062)****	
20-30	54	14	2.493 (0.975-6.379)	
30-40	29	7	1.944 (0.608-6.208)	
>40	68	26	**4.277 (1.699-10.763)****	
**Time of arrive to arrival**				0.641
Within 2 hours	160	22	1	
After 2 hours	204	56	0.835 (0.392-1.780)	
**Laboratory test**				0.000
Yes	111	11	1	
No	253	67	**3.904 (1.880-8.105)*****	

The cut point for significant association is at $p<0.05$
*p < 0.05, **p < 0.01, ***p < 0.001

Discussion

This study dedicated to investigate poisoning in concern with the poisoning patterns in relation to socio-demographic characteristics, the type of poisoning agents involved, treatments given and associated factors with clinical outcomes as death or cure among poisoning cases attended the selected referral hospitals in Amhara Regional state, Ethiopia. The main findings of the study are explained and/or compared and contrasted with previously published findings of similar studies.

The poisoning incidence may vary with different categories of socio-demographic characteristics such as by age group, sex, residence, and occupation. In this study, the majority of the poisoning cases were females (63.12%). This result is consistent with the results of previous studies in Ethiopia and Taiwan reported 59.20% and 54.76% of incidences, respectively, were females (8, 15). More than half, 53.17%, of the cases were in the age group of 20-40 years and this is in line with the results of similar studies (9, 16). Regarding residence, the poisoning incidence was

found somewhat greater in the urban areas; 51.13% in urban and 48.87% in rural areas. This difference may be at least in part because of some of the cases from the rural areas may not attend hospitals as they may be treated in health stations and health posts in their locality. Considering distribution of the cases by marital status, 50.45% of the poisoned patients were single. This proportion is lower than reports of similar studies in Addis Ababa, Ethiopia and Selected Hospitals in Western Ethiopia which are 54.98% and 57.10%, respectively (8, 17). The majority of the cases (28.96%) were illiterates followed by those with educational level of secondary school (26.24%). Majority of the poisoning cases were students (31.45%) followed by farmers (29.41%). This may be due to that students are prone to emotional liability associated with academic failure and love related issues leading to intentional poisoning for suicide purpose. The majority of our community is farmer in occupation and this can be the reason for the higher incidence of poisoning cases was from farmers.

As it can be depicted from Table 2, the time of arrival to the health facilities seems correlated with the distance from the patients' residential areas to the hospitals. Accordingly, as the distance category from which the patients come is more remote the proportion of the patients arrived after 2 hours of the poisoning incidence become higher though not at all points. Moreover, greater than half, 58.82%, of the cases were arrived to the hospitals after 2 hours of poisoning incidence. This may negatively affect the treatment outcome as the poisoning is a medical emergency that should be treated as soon as possible after its occurrence. Also regarding their consciousness when arrived at the emergency department of the hospitals, more than half of the cases, 52.94%, were arrived with their level of consciousness compromised; out of the total cases, 24.21% were in semiconscious and 28.73% in unconscious stated upon arrival. Most of the deaths were among the patients arrived with their level of consciousness compromised, especially in those that were unconscious. This shows that treatment outcome might be affected by the state of consciousness of the victims when arrived at the health facilities. Therefore, it is advisable to come to the health facilities soon after the incidence of poisoning before the symptoms of poisoning is worsened and consciousness is altered.

The signs and symptoms manifested in the poisoning cases were nausea and vomiting, epigastric pain, salivation and/or generalized sweating, hypothermia, hypotension, headache, and diarrhea among others from the most to least frequently reported signs in order. The spectrum of the

signs and symptoms of toxicity seen in this study are similar with those reported by other studies of poisoning cases (17, 18). Nausea and vomiting and epigastric pain were the first and second most frequently encountered symptoms of toxicity and this may be due to direct irritant effects of the poisoning agents to gastrointestinal tract as the most frequently employed route of exposure was the oral route. Regarding the poisoning agents involved, organophosphate compounds, and metal phosphides (majorly aluminum phosphide), were the first and the send most commonly reported causes of poisoning followed by other agents such as bleaching agents, medicines, unknown chemicals regarded as rat poison, alcohol and carbon monoxide in decreasing order of frequency. Concerning organophosphates, this result is consistent with the findings of other studies reporting these compounds as the most frequently encountered poisoning agents (16, 19, 20, 21), but inconsistent with another study at Jimma University Specialized Hospital (10). In Ethiopia organophosphate compounds and aluminum phosphide are widely available as pesticides. This may increase the availability and ease their access for intentional poisoning as seen in this study.

As shown in Table 5 of the results section, the majority of poising cases were caused by poisons of solid and liquid dosage forms and the commonest route of exposure was the oral route. The liquid dosage forms with other routes of exposure were snake venoms because of five snake bite accidents encountered in this study and one topical exposure of an organophosphate compound. Inhalational route of exposure was for gaseous forms of toxicants and majorly of carbon monoxide poisoning. With respect to the sources of the poisoning agents, the majority were obtained at the residential homes and local shops. This result is in agreement with the results of other studies in that most of the poisoning agents were obtained at home (9, 17). This may be because of the inappropriate storage of the poisoning agents at home especially when leftover from their use as insecticides or against grain storage pasts. The second most frequently reported source of poison agents was shop and is in line with the result report by a similar study carried out in western Ethiopia (8). The most common reason for intentional poisoning was dispute related, mainly family disharmony, followed by psychiatric illnesses, love affairs, and unknown reason among additional others. The reasons reported in this study are similar with those in a systematic review by Chelkeba *et al* (22). Furthermore, regarding the most frequently reported reason for poisoning, the result of this study is in line with other previous studies (19, 23).

Both pharmacologic and nonpharmacologic methods of treatment were employed in managing the poisoning cases. The most frequent use of cimetidine may be, at least in part, because of an assumption that most poisoning cases have acute gastroenteritis and suggested by that epigastric pain is the most frequently encountered symptom across all the cases. Atropine was the second most commonly employed pharmacologic agent in the treatment of poisoning and this is because it is an antidote for organophosphorus poisoning, which was found to be the most common cause of poisoning in this study. The use of IV magnesium sulphate may be encouraged based on reports, although inconclusive, that it has promising effects as adjunctive treatment in acute organophosphorus insecticide poisoning (24, 25). In addition to this it has been demonstrated to have significant effect on survival of patients in aluminum phosphide poisoning (26). The pharmacologic agents used also include antibiotics, hydrocortisone, omeprazole, frusemide, and dopamine among others. The uses of these agents against various poisoning cases and their suggested benefits have been described in different previous studies (27, 28). The most commonly employed nonpharmacologic interventions were supportive cares (ABC, mainly IV resuscitation) and this finding is favorable as ensuring a protected airway, adequate ventilation and hemodynamic stability through ABC-approach is crucial in the management of poisoning emergency (22). Gastric lavage was the second most frequently used nonpharmacologic approach for poisoning management. But it is not recommended for routine use and should not be considered unless a patient has ingested a potentially life-threatening amount of a poison (29). Interestingly, among all the cases dialysis was used only for one case. This may be because of unavailability of the facility or unaffordability of the procedure but might not be because of lack of the need for this intervention.

The duration of hospital stay of each case was considered as of from the time of admission to termination of hospital stay because of discharge following recovery from poisoning symptoms or because of death. Based on this the majority (59.95%) of the cases were hospitalized for not longer than 24 hours. As it can be depicted from Figure 2, most of the death records were from the cases with hospital stays of ≤24 hours, accounting 15.38% of the deaths out of the total 17.65% fatality rate in this study. This is because as the cases died no further care is required and immediately discharged from the hospitals and not indicative of a relation between short duration of stay and high fatality rate. In the other time categories the fatality rates were lower and even no record of fatality was noted among the cases hospitalized for longer than 72 hours. The mean

duration of hospital stay was found to be 34.37 hours. This is longer than the findings of previous study by Adinew *et al* (23) and Dessalew *et a*l (30) which are 11.26 hours and 1 day, respectively.

The case fatality rate from acute poisoning was found to be 17.65%. This finding is much higher than previous findings of similar studies carried out in Ethiopia (8, 10, 15, 17, 31) and India (16), ranging 4.10-10.20%. Out of total 78 cases died, aluminum phosphide was responsible for 55.13% of the deaths, whereas organophosphates were attributed to 32.05% of deaths. Even though bleaching agents were the third most common cause of poisoning in this study, no death was recorded among the victims poisoned by these agents. Organophosphates, aluminum phosphide, and other chemicals are available in Ethiopia for use as rodenticide, insecticide or herbicide (32) and a comparative study showed the Ethiopian officers that handle these chemicals are less knowledgeable about these products (33). Aluminum phosphide is widely available in Ethiopia with approved use for the control of maize weevil and flour beetle on stored maize (32). Organophosphate compounds, especially Malathion, are also among commonly available pesticides for agricultural uses (33). Their availability and ease of accessibility plus the inherent to toxicity is likely to pose a great health burden in the community. This result may dictate the need for the design and implementation of strategies to create awareness about and prevent the poisonings by such frequently encountered agents in the community.

Multivariate logistic regression analysis was done to identify predictors of treatment outcome in the present study. Accordingly age, level of consciousness upon hospital arrival, distance from the place of poisoning incidence to the hospital, and use of laboratory test as part of diagnosis were identified as significant potential predictors of treatment outcome. The analysis showed that the cases aged 20–40 and >40 years had 2.106 and 3.630 times of likelihood for death, respectively, than those aged ≤20 years. In this regard, our study is consistent with a study conducted to determine whether age matters in the case fatality rate for selected poisonings and reported the likelihood for death as outcome is increased with increasing age category (34). This is also in agreement with another study that showed higher age contributed to poor treatment outcome (31). Base on level of consciousness, the patients that were in semiconscious and unconscious state upon hospital arrival were 2.673 and 5.296 times more likely to die, respectively, than who were conscious. The level of consciousness may be compromised due to delay of intervention or because of inherent toxic characteristics of a poisoning agent leading

rapid loss of consciousness and affect the treatment outcome negatively. Considering the distance from the place of poisoning incidence to the hospitals attended, the odds of the likelihood to die were generally higher, even though inconsistent, for the patients that were from more remote areas during the incidence. This may be because if the places from where the victims were during the incidence are remote, they will not arrive to the hospitals short time after the occurrence of the poisonings and hence late interventions. In addition, the odds of death among poisoned patients who had no laboratory result as part of their diagnosis process was 3.904 times than of those whose diagnosis involves laboratory methods. This might be because the laboratory diagnosis can help investigate the severity of toxicity and direct to better treatment intervention accordingly.

The prospective nature of the study design enables us to collect detail clinical and laboratory data to determine the severity of the cases as a determinant of treatment outcome. This can be taken as the strength of this study (30). But this study lacks some parameters including pre-hospital interventions (that might be given at home or local health facilities), referral cases, and appropriateness of interventions at the hospitals. Missing of such variables can be considered as the limitations of this study. If these variables were included, it would be better for more appropriate explanation of potential predictor variables of the treatment outcome. For example, the likelihood of death was not significantly increased with time of delay for hospital arrival and this might be because of pre-hospital interventions at home or local health centers and does not indicate that time of delay to come after intoxication has no negative impact on treatment outcome.

Conclusion

The majority of the poisoning cases were fem[illegible]. The first and second most frequently encountered poisoning agents were organophosphate compounds and aluminum phosphide, respectively, and it was noted that the later one was responsible for most of the fatal cases. The fatality rate of poisoning in this study was found to be much higher than in other similar studies. Older ages, impaired consciousness upon hospital arrival, distance from the hospitals, and no involvement of laboratory investigation were found to significantly associate with the likely hood of death.

Availability of data

The datasets of the study can be obtained from the corresponding author on reasonable request.

Authors' contributions

All authors were participated in the conception, design, and development of the proposal of the study for grand competition and in supervision of the data collection process. GMA had substantial contribution in the preparation of the data collection tool. ABA accomplished the data entry, analysis, interpretation, and the writing up of the manuscript. Finally, all authors read and approved the submission of the manuscript for publication. Moreover, all of the authors are responsible regarding the appropriateness and originally of this work.

Acknowledgements

We would like to thank University of Gondar for its financial support which was used for the data collection, supervision, and related costs of this study. `

Competing interests

We have no competing interests regarding this work.

Ethics approval and consent to participate

The protocol of the study was reviewed and approved by University of Gondar, College of Medicine and Health Sciences, Ethical Review Committee. In addition, each hospital administration office was requested by formal letter from the University of Gondar, College of Medicine and Health Sciences, Research and Community Service Office and permission was obtained from each hospital to conduct the study. Furthermore, participant informed consent to participate in the study was obtained from each patient and/or care giver before the data collection. Those who were unwilling to participate were excluded. No personal identifier was included in the data collection tool and the data record was kept confidential and used only for the study purpose.

Funding

This study was done using a grant awarded by University of Gondar, Ethiopia, Reference № R/T/T/C/Eng.58/03/2018.

References

1. DesLauriers C. Poisoning: A Public Health Problem. *Pharmacy Tech Topics*. 2015; 20(1):1-21.
2. WHO. Acute & Chronic Poisoning effects. 2016

3. G/Mariam ET, Gelaw BK. Global Epidemiology of Acute Poisoning with an Emphasis to Ethiopia: Systematic Review. *Int J Pharm Sci & Scient Res*. 2016; 2(4): 120-130.
4. Sawalha AF, Sweileh WM, Tufaha MT, Al-Jabi DY. Analysis of the pattern of acute poisoning in patients admitted to a governmental hospital in Palestine. *Basic Clin Pharmacol Toxicol*. 2010; 107(5):914-918.
5. WHO. Poisoning Prevention and Management. 2016
6. Abd-Elhaleem ZAE, Muqhem BAA. Pattern of acute poisoning in Al Majmaah region, Saudi Arabia. *Am J Clin Exp Med*. 2014; 2(4): 79–85.
7. Kebede D, Alem A. Suicide attempts and ideation among adults in Addis Ababa, Ethiopia. *Acta Psychiatr Scand Suppl*. 1999; 397:35-39.
8. Woyessa AH, Palanichamy T. Patterns, Associated Factors, and Clinical Outcomes of Poisoning among Poisoning Cases Presented to Selected Hospitals in Western Ethiopia: Hospital-Based Study. *Emerg Med Int*. 2020; 2020:5741692.
9. Getie A, Belayneh YM. A Retrospective Study of Acute Poisoning Cases and Their Management at Emergency Department of Dessie Referral Hospital, Northeast Ethiopia. *Drug Healthc Patient Saf*. 2020;12:41-48.
10. Teklemariam E, Tesema S, Jemal A. Pattern of acute poisoning in Jimma University Specialized Hospital, South West Ethiopia. World J Emerg Med. 2016;7(4):290-293.
11. Singh S, Sharma BK, Wahi PL, Anand BS, Chugh KS. Spectrum of acute poisoning in adults (10 year experience). *J Assoc Physicians India*. 1984 Jul;32(7):561-3. PMID: 6501193.
12. Padmanabha TS, Gumma K, Kulkarni GP. Study of profile of organophosphorus poisoning cases in a tertiary care hospital, North Karnataka, Bidar, India. *Int J Pharm Bio Sci*. 2014;5(1):332-339.
13. Kumar S, Pathak A, Mangal HM. Trends of fatal poisoning in Saurashtra region of Gujarat (a prospective study). *Journal of Indian Academy of Forensic Medicine*. 2011; 33(3):197-199.
14. Somasundaram KV, Patil A, Shukla SK. Epidemiological profile of OP poisoning cases treated at Pravara hospital, Loni, India. *Indian J Prev Soc Med*. 2009;40(3&4):184-188.
15. Lee HL, Lin HJ, Yeh ST, Chi CH, Guo HR. Presentations of patients of poisoning and predictors of poisoning-related fatality: findings from a hospital-based prospective study. *BMC Public Health*. 2008;8:7.

16. Kumar SV, Venkateswarlu B, Sasikala M, Kumar GV. A study on poisoning cases in a tertiary care hospital. *Journal of natural science, biology, and medicine*. 2010;1(1):35.
17. Zemedie B, Sultan M, Zewdie A. Acute Poisoning Cases Presented to the Addis Ababa Burn, Emergency, and Trauma Hospital Emergency Department, Addis Ababa, Ethiopia: A Cross-Sectional Study. *Emerg Med Int*. 202; 2021:6028123.
18. Alghafees MA, Abdulmonen A, Eid M, Alhussin GI, Alosaimi MQ, Alduhaimi GS, Albogami MT, Alhelail M. Poisoning-related emergency department visits: the experience of a Saudi high-volume toxicology center. *Ann Saudi Med*. 2022;42(1):36-44.
19. Adinew GM, Woredekal AT, DeVos EL, Birru EM, Abdulwahib MB. Poisoning cases and their management in emergency centres of government hospitals in northwest Ethiopia. *Afr J Emerg Med*. 2017;7(2):74-78.
20. Jamie AH, Abdosh MZ. Patterns and Clinical Outcomes of Poisoning Among Poisoning Cases Presented in a Hospital: A Retrospective Study. *Lokman Hekim Health Sci*. 2021;1(3):70–73.
21. Ramesha KN, Rao KB, Kumar GS. Pattern and outcome of acute poisoning cases in a tertiary care hospital in Karnataka, India. *Indian J Crit Care Med*. 2009;13(3):152-155.
22. Chelkeba L, Mulatu A, Feyissa D, Bekele F, Tesfaye BT. Patterns and epidemiology of acute poisoning in Ethiopia: systematic review of observational studies. *Arch Public Health*. 2018 Jul 2;76:34.
23. Adinew GM, Asrie AB, Birru EM. Pattern of acute organophosphorus poisoning at University of Gondar Teaching Hospital, Northwest Ethiopia. BMC Res Notes. 2017;10(1):149.
24. Pajoumand A, Shadnia S, Rezaie A, Abdi M, Abdollahi M. Benefits of magnesium sulfate in the management of acute human poisoning by organophosphorus insecticides. *Hum Exp Toxicol*. 2004;23(12):565-569.
25. Jamshidi F, Yazdanbakhsh A, Jamalian M, Khademhosseini P, Ahmadi K, Sistani A, Jokar A. Therapeutic Effect of Adding Magnesium Sulfate in Treatment of Organophosphorus Poisoning. *Open Access Maced J Med Sci*. 2018;6(11):2051-2056.
26. Chugh SN, Kumar P, Aggarwal HK, Sharma A, Mahajan SK, Malhotra KC. Efficacy of magnesium sulphate in aluminium phosphide poisoning--comparison of two different dose schedules. J Assoc Physicians India. 1994;42(5):373-375.

27. Priyendu A, Vandana KE, Nilima, Varma M, Prabhu N, Rahim AA, Nagappa AN. Antibiotic prophylaxis in organophosphorus poisoning: A study of health and economic outcomes. *Saudi Pharm J*. 2017;25(3):332-336.
28. Katwal S, Malbul K, Mandal SK, Kc S, Alam MZ, Karki P, Pant C. Successfully managed aluminum phosphide poisoning: A case report. *Ann Med Surg (Lond).* 2021; 70:102868.
29. Vale JA. Position statement: gastric lavage. American Academy of Clinical Toxicology; European Association of Poisons Centres and Clinical Toxicologists. *J Toxicol Clin Toxicol*. 1997;35(7):711-719.
30. Desalew M, Aklilu A, Amanuel A, Addisu M, Ethiopia T. Pattern of acute adult poisoning at Tikur Anbessa specialized teaching hospital, a retrospective study, Ethiopia. *Hum Exp Toxicol*. 2011;30(7):523-527.
31. Tefera GM, Teferi LG. Prevalence, Predictors and Treatment Outcome of Acute Poisoning in Western Ethiopia. *Open Access Emerg Med*. 2020;12:365-375.
32. Ethiopian Ministry of Agriculture. List of registered pesticides. Accessed on July 17, 2022. http://www.moa.gov.et/list-of-registered-pesticides
33. Tessema RA, Nagy K, Ádám B. Pesticide Use, Perceived Health Risks and Management in Ethiopia and in Hungary: A Comparative Analysis. *Int J Environ Res Public Health*. 2021;18(19):10431.
34. Rogers JJ, Heard K. Does age matter? Comparing case fatality rates for selected poisonings reported to U.S. poison centers. *Clin Toxicol (Phila)*. 2007;45(6):705-708.

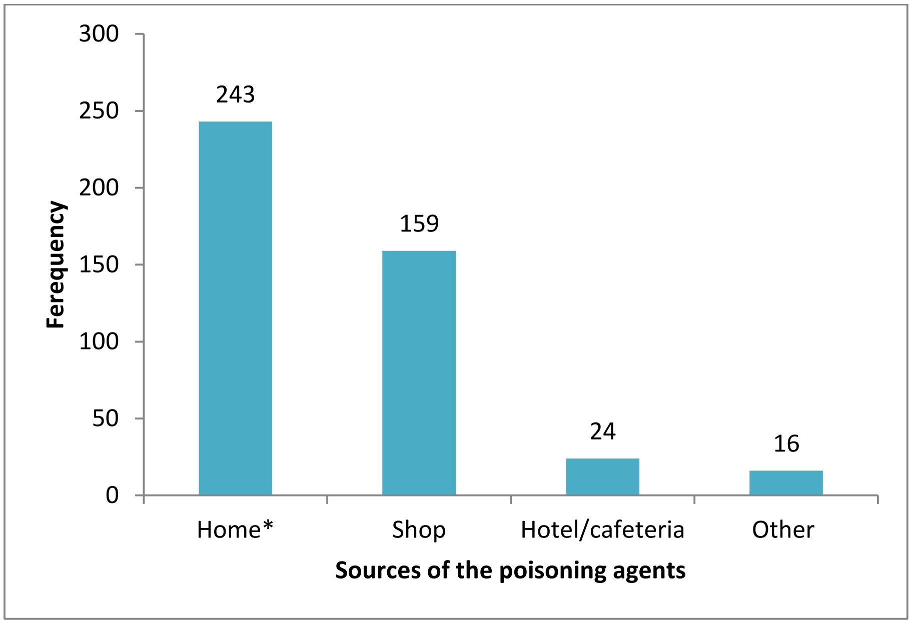

Figure 1 Source of poisons used for poisoning of patients attending the hospital emergency departments. *Includes homes other than they were living in.

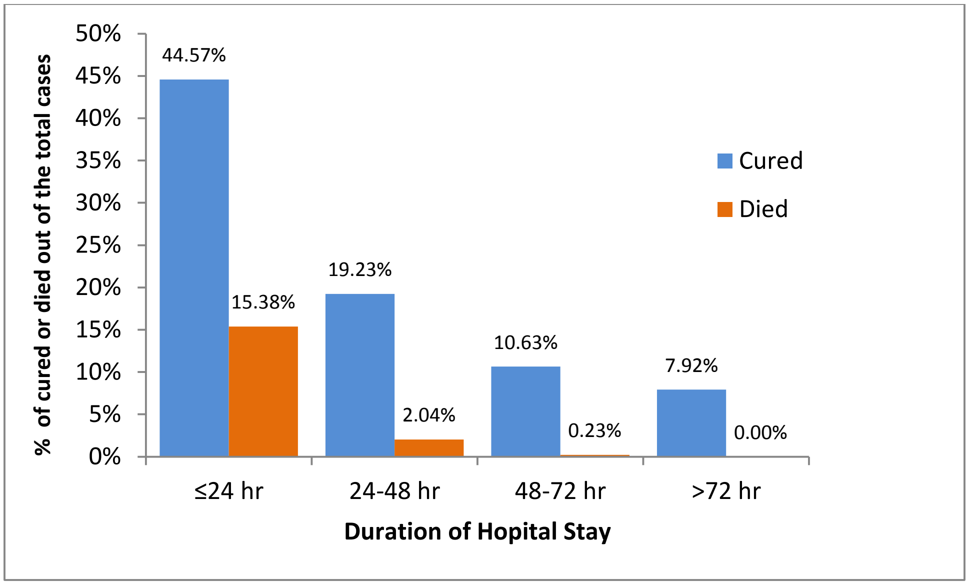

Figure 2 Duration of hospital study and treatment outcome.

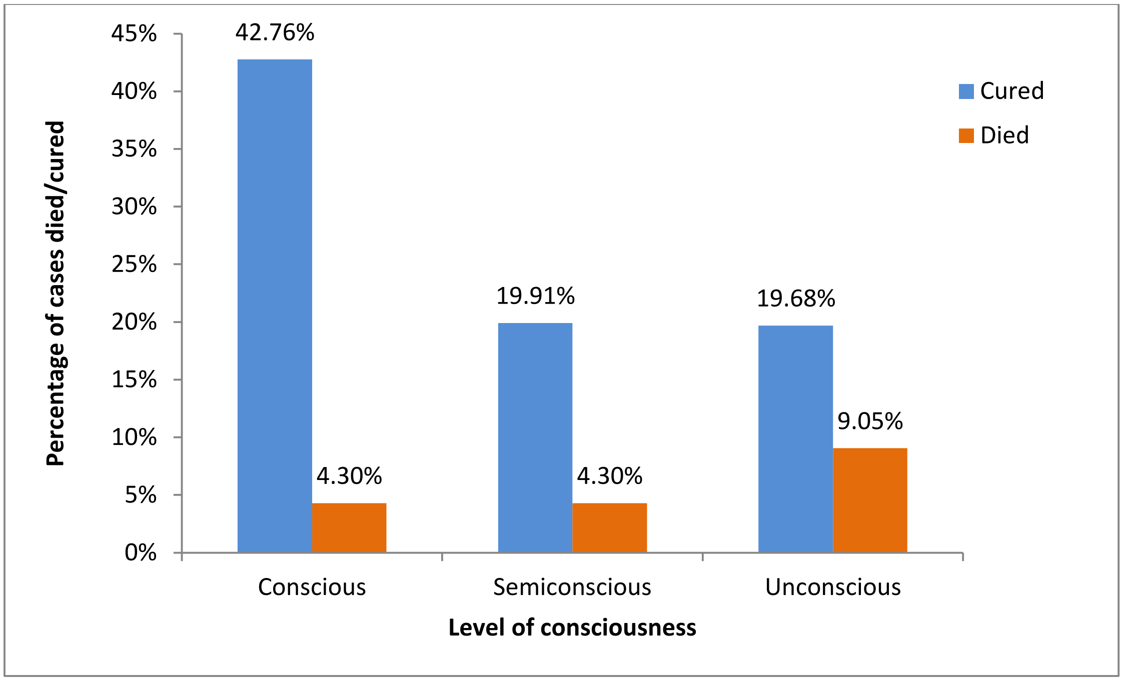

Figure 3 Patient-consciousness upon arrival to the hospital and the treatment outcome.
