# Supplementary material for: Poisoning cases and their management in Amhara National Regional State, Ethiopia: Hospital-based prospective study
Source: PLoS One. 2024 May 31;19(5):e0303438. doi: 10.1371/journal.pone.0303438 (PMC11142576; doi:10.1371/journal.pone.0303438)
Supplement: Attachment — Submitted filename: PONE-D-22-26894_R3_Edit.pdf [file pone.0303438.s005.pdf]

PLOS ONE

Poisoning Cases and Their Management in Amhara Region, Ethiopia: Hospital-Based Prospective Study

--Manuscript Draft--

Manuscript Number:	PONE-D-22-26894R3
Article Type:	Research Article
Full Title:	Poisoning Cases and Their Management in Amhara Region, Ethiopia: Hospital-Based Prospective Study
Short Title:	Poisoning cases and Their Management
Corresponding Author:	Assefa Belay Asrie, MSc<br>University of Gondar<br>Gondar, Amhara National Regional State ETHIOPIA
Keywords:	Poisoning, poisoning agents, organophosphate compounds, metal phosphides, poisoning management, treatment outcome
Abstract:	Background: Poisoning is a significant public health problem globally. Ethiopia is a low-income country undergoing technological and social change that may increase access to drugs and chemicals, potentially increasing the incidence of poisoning. This study describes the epidemiology of hospital admissions due to poisoning in a region of Ethiopia.<br>Methods: An institution based prospective observational study was employed, as a study design, in selected hospitals of the region from January, 2018 to December, 2018.<br>Results: Of 442 poisoning cases, 78 (17.6%) were died. Almost all poisoning cases were intentional self-poisonings. The most frequent poisonings were organophosphate compounds, 145 (32.8%), and metal phosphides (majorly aluminum phosphide), 115 (26.0%). The ingested poison was most frequently accessed from the patients' home, 243 (55.0%), followed by purchasing from local shops, 159 (36%). The median duration of admission was 24 hours. Of all the cases, 23 (5.2%) were admitted to intensive care unit (ICU) requiring mechanical ventilation. Most of the cases admitted to the ICU were aluminum phosphide-poisoned patients. The majority of deaths (43 of 78) were due to metal phosphides. From the multivariate logistic regression analysis, age, altered level of consciousness on hospital arrival, distance from the area where the poisoning incidence happened to the hospital, and no laboratory result as a part of the diagnosis process or investigation of the extent of toxicity were found to be significantly associated with the likelihood of poor treatment outcome.<br>Conclusion: The majority of the poisoning cases were females. The most common reasons for the intent of self-poisoning were dispute-related, mainly family disharmonies, followed by psychiatric conditions. The poisoning agents were mostly obtained from households. Organophosphate compounds and metal phosphides were the first and the second most frequently encountered poisoning agents, respectively, and it was noted that the later ones were responsible for most of the fatal cases. Of the pharmacologic, atropine was the only agent regarded as antidote. The most commonly employed agent for supportive treatment was cimetidine followed by maintenance fluids, while gastric lavage was the only GI decontamination method used among others. Cimetidine was the most often used agent for supportive treatment, followed by maintenance fluids and gastric lavage was the only GI decontamination techniques that was utilized. The fatality rate of poisoning in this study was found to be much higher than in other similar studies. Impaired consciousness upon hospital arrival, type of poisoning, and no involvement of laboratory investigation were found to significantly associate with the likelihood of death. Generally the results dictate the need for the design and implementation of strategies to create awareness, prevent, and manage poisoning incidences in the community.
Order of Authors:	Assefa Belay Asrie, MSc
	Seyfe Asrade Atnafie, MSc
	Kefyalew Ayalew Getahun, MSc

Powered by Editorial Manager® and ProduXion Manager® from Aries Systems Corporation

	Eshetie Melese Birru, MSc
	Gashaw Binega Mekonnen, MSc
	Geta Asrade Alemayehu, MPH
	Berhanu Fikadie Endehabtu, MPH
	Marta Berta Badi, PhD Candidate
	Getinet Mequanint Adinew, MSc
**Response to Reviewers:**	Response to Reviewers<br>Manuscript Title: Poisoning Cases and Their Management in Amhara Region, Ethiopia: Hospital-Based Prospective Study<br>Manuscript Submission Number: PONE-D-22-26894R2<br>Journal: PLOS ONE<br>Dear Editor,<br>We are very thankful to you for handling the publication process of this manuscript. Modified portions or newly added texts in the revision process are marked-up with greed color, whereas deletions are shown by red colored texts with Strikethrough line.<br>Response to Reviewer #3<br>Dear Reviewer,<br>We are grateful for your dedication and time spent in reviewing this manuscript. The comments forwarded are greatly appreciated and used to help improve the manuscript. They have been addressed as follows.<br>Reviewers' comments:<br>Reviewer's Responses to Questions<br>Comments to the Author<br>1.If the authors have adequately addressed your comments raised in a previous round of review and you feel that this manuscript is now acceptable for publication, you may indicate that here to bypass the “Comments to the Author” section, enter your conflict of interest statement in the “Confidential to Editor” section, and submit your "Accept" recommendation.<br>Reviewer #3: (No Response)<br>2.Is the manuscript technically sound, and do the data support the conclusions?<br>Reviewer #3: Partly<br>Thank you!<br>By taking into account the feedback you sent we have made changes to improve the manuscript in this regard. We include additional points in the conclusion part.<br>3.Has the statistical analysis been performed appropriately and rigorously?<br>Reviewer #3: I Don't Know<br>As the result is dichotomous, i.e., survived or died, we have performed binary logistic regression adjusted to potential factors that might affect patient outcome (as cured or died). First we performed bivariate logistic regression to identify potential predictors of the patient outcome. Then the factors found to significantly associate with probability of death in bivariate analysis were included in multivariate analysis. We performed backward stepwise elimination logistic regression and we selected the best model among the alternative models found in the analysis.<br><br>4.Have the authors made all data underlying the findings in their manuscript fully available?<br>Reviewer #3: No<br>We think that we made sufficient data underlying the findings available in the manuscript. Moreover, the full data set was submitted to the journal during manuscript submission. However, as another reviewer commented that there are too many tables and figures and the result could be summarized in 2-3 tables, 2 tables and 2 figures have been removed in this version.<br>5.Is the manuscript presented in an intelligible fashion and written in standard English?<br>Reviewer #3: No<br>Thanks! We have reviewed the entire document and made a number of changes to fix grammatical errors. We corrected some grammatical errors using QuillBot online grammar checker. Additionally, the document was revised for language clarity by Dr. Yoseph, a senior English language professional (PhD in Teaching English as a Foreign Language (TEFL)) at University of Gondar, and further corrections were made based on his recommendation.<br>6.Review Comments to the Author

Reviewer #3: You need be more specific about design, although it is not explicitly stated it appears that you targeted a total of 440 patients recruiting 110 in each site. If that is the case you should say that explicitly including whether all consecutive patients were approached, how many refused (and include some basic information about the refusal such as the poison they were exposed to , gender and age) Further how Lon did it take you in each site to recruit the patients eg how many weeks
Thank you for your concern!
The number of cases targeted was determined to be 442 (based on sample size calculation, page 4 of the previous version of the manuscript). And then, the subjects were divided to the four hospitals considered in this study.
−University of Gondar Comprehensive Specialized Hospital = 110 patients
−Felege Hiwot Referral Hospital = 110 patients
−Debre Markos Referral Hospital = 111 patients
−Dessie Referral Hospital = 111 patients
The number of cases (110 or 111) to be targeted from each site was determined simply by lottery method.
From preliminary assessment we made before the data collection, we had been informed that irregular numbers of poisoning cases attend each hospital. Referring log books in the emergency departments and communicating the healthcare professionals working in the emergency department of each hospital, we had been informed that the number of poisoning cases attending the departments in each year is unpredictable and roughly ranges 80 to 130 patients per year. Because of this we couldn’t approach randomly selected cases and rather we decided to and approached all consecutive cases until we got the number of cases taken from each hospital. Very few cases refused to participate in the study (refusal was so rare) and had been excluded from the study. In addition, very few cases from whom adequate data were not obtained had been excluded from the study. However, we collected no data from such cases so that we have no information about them at hand.
The data collection was started on the same date at all sites, whereas there were some differences in the length of time needed to finish the data collection at each site. In all hospitals, the data collection was started on January 01, 2018 and completed in December 2018 on different specific dates shown below.
−University of Gondar Comprehensive Specialized Hospital = on December 26, 2018
−Felege Hiwot Referral Hospital = on December 24, 2018
−Debre Markos Referral Hospital = on December 21, 2018
−Dessie Referral Hospital = on December 29, 2018
It was taken almost a year to complete the data collection at each site. For this reason, we stated that the data collection period ran from January to December of 2018 (to collect the data from the total of 442 cases).
Response to Reviewer #4
Dear Reviewer,
Thank you for your time and effort invested in reviewing this manuscript. The comments raised are highly valued and used as input to improve the manuscript. They have been addressed as follows:
Reviewers' comments:
Reviewer's Responses to Questions
Comments to the Author
1.If the authors have adequately addressed your comments raised in a previous round of review and you feel that this manuscript is now acceptable for publication, you may indicate that here to bypass the “Comments to the Author” section, enter your conflict of interest statement in the “Confidential to Editor” section, and submit your "Accept" recommendation.
Reviewer #4: (No Response)
2.Is the manuscript technically sound, and do the data support the conclusions?
Reviewer #4: No
Thank you!
We think that this issue has been improved through addressing the feedbacks you provided under the "Review comments to Author".
3.Has the statistical analysis been performed appropriately and rigorously?
Reviewer #4: No
As the outcome measure is dichotomous, i.e., survived or died, we have performed binary logistic regression adjusted to potential factors that might affect the outcome (as cured or died). Initially, we performed bivariate logistic regression to identify potential predictors of the patient outcome. Then the factors that were found to be significantly

associated with the probability of death in bivariate analysis were included in multivariate analysis. We performed backward stepwise elimination logistic regression and we selected the best model among the alternative models found in the analysis.
4.Have the authors made all data underlying the findings in their manuscript fully available?
Reviewer #4: Yes
5.Is the manuscript presented in an intelligible fashion and written in standard English?
Reviewer #4: No
We reviewed the manuscript to increase the clarity and ease of understandability. Accordingly, we made many changes at various sections in the process of revision.
6.Review Comments to the Author
Reviewer #4: Manuscript ID: PONE-D-22-26894R2
Thank you for the opportunity to review this manuscript entitled: “Poisoning Cases and Their Management in Amhara Region, Ethiopia: Hospital Based Prospective Study”
There are still numerous errors relating to grammar, syntax and academic one. I suggest that the authors employ the services of a professional English language editor with a background in medical journal articles
We have gone through the whole document and made numerous amendments in this regard. First we made corrections on grammatical errors using “quillbot online grammar checker”. Additionally, the document was revised for language clarity by Dr. Yoseph, a senior English language professional (PhD in Teaching English as a Foreign Language (TEFL)) at University of Gondar, and further corrections were made based on his recommendation. The manuscript has been also revised for academic issues by Dr. Zewdneh Shewamene Sabe (working at Population Council, Ethiopia) and additional corrections were made (eg, deletion of portions in the result and discussion parts). But we are unable to get professional language editor with a background in medical journal articles.
In the Methods section of the Abstract it is stated that data collection was from Jan to Dec 2028, which implies that a convenience sampling was used. However, in the main text under Methods, the authors indicate that sample size determination was used. This is conflicting and needs to be clarified.
Thank you!
First, we determined the number of cases to be included in the study by calculating the sample size as stated in the manuscript (not conveniently determined). Then, we distributed the 442 samples to the four study sites; 111 cases to each of two hospitals and 110 cases to each of the other two hospitals. After this, we started data collection and the period stated is the time required to collect the data in each hospital.
At all sites, the data collection was started on January 1, 2018 and completed in the fourth week of December 2018 on different specific dates. For this reason, we stated that the data collection period ran from January to December of 2018. The time was not predetermined before the data collection. We determined the sample cases to be considered first, distributed them to the four study sites. It took the stated period (January to December 2018) to complete the data collection at each site.
As of the information we got from the emergency departments of the hospitals before starting the data collection, the cases flow was not predictable. Because of this we couldn’t follow a specific sampling technique and rather we decided to consider all consecutive cases till we got the specified number of patients in each hospital. There were few refusal cases and few others with incomplete data and excluded from the study. However, since the data was collected until we achieved the predetermined number of subjects in each study site; the total cases targeted are equal to the originally calculated sample size.

Overall, there are too many tables and figures. Most of the data can be included in 2-3 tables. Also, there is a lot of repetition between the text and figures/tables.
Thank you!
In consideration of this comment, 2 tables and 2 figures that we feel are redundant or do not contain as much relevant information have been removed. The discussion points pertaining to the contents of these tables and figures have also been eliminated. These are the figures and tables that have been removed.
−Table 2 on Page 7…Variable included in the multivariate analysis
−Table 4 on page 8…We think the content is not as much relevant.
−Figure 2 on page 11…The variable included in the multivariate analysis
−Figure 3 on page 12….Because the content appeared in the multivariate analysis result table.

	Table 3: Seems like “Died” and “Cured” under “Treatment outcome” have been incorrectly labelled. The authors are encouraged to read through other similar publications which will assist them with improving the layout and design of this article.<br>The discussion section is too long.<br>Thank you!<br>We replaced the term “died” by “cured” and the vice versa.<br>The discussion part has been reduced from 5 and half pages to about 4 pages.<br><br>Please note that:<br>Texts with red font color and strikethrough line – indicate deletion in the process of revision.<br>Texts with green highlight – indicate newly added texts in the revision process.
**Additional Information:**	
**Question**	**Response**
**Financial Disclosure**<br><br>Enter a financial disclosure statement that describes the sources of funding for the work included in this submission. Review the submission guidelines for detailed requirements. View published research articles from *PLOS ONE* for specific examples.<br><br>This statement is required for submission and **will appear in the published article** if the submission is accepted. Please make sure it is accurate.<br><br>**Unfunded studies**<br>Enter: *The author(s) received no specific funding for this work.*<br><br>**Funded studies**<br>Enter a statement with the following details:<br>• Initials of the authors who received each award<br>• Grant numbers awarded to each author<br>• The full name of each funder<br>• URL of each funder website<br>• Did the sponsors or funders play any role in the study design, data collection and analysis, decision to publish, or preparation of the manuscript?<br>• **NO** - Include this sentence at the end of your statement: *The funders had no role in study design, data collection and analysis, decision to publish, or preparation of the manuscript.*<br>• **YES** - Specify the role(s) played.<br><br>* typeset	This study was done using a grant awarded by University of Gondar, Ethiopia (Reference № R/T/T/C/Eng.58/03-2018). The funder had no role in the study design, data collection and analysis, decision to publish, or preparation of the manuscript.
**Competing Interests**	The authors have declared that no competing interests exist.

Powered by Editorial Manager® and ProduXion Manager® from Aries Systems Corporation

Use the instructions below to enter a competing interest statement for this submission. On behalf of all authors, disclose any competing interests that could be perceived to bias this work—acknowledging all financial support and any other relevant financial or non-financial competing interests.<br><br>This statement is required for submission and **will appear in the published article** if the submission is accepted. Please make sure it is accurate and that any funding sources listed in your Funding Information later in the submission form are also declared in your Financial Disclosure statement.<br><br>View published research articles from *PLOS ONE* for specific examples.<br><br>**NO authors have competing interests**<br><br>Enter: *The authors have declared that no competing interests exist.*<br><br>**Authors with competing interests**<br><br>Enter competing interest details beginning with this statement:<br><br>*I have read the journal's policy and the authors of this manuscript have the following competing interests: [insert competing interests here]*<br><br>* typeset	
**Ethics Statement**<br><br>Enter an ethics statement for this submission. This statement is required if the study involved:<br><br>• Human participants<br>• Human specimens or tissue<br>• Vertebrate animals or cephalopods<br>• Vertebrate embryos or tissues	The protocol of the study has been reviewed and approved by the Institutional Ethical Review Board of University of Gondar (Reference №: O/V/P/RCS/05/372/2018). In addition, each hospital administration office was requested by formal letter from the University of Gondar, College of Medicine and Health Sciences, Research and Community Service Office and permission was obtained from each hospital to conduct the study. Furthermore, participant informed consent to participate in the study was obtained from each patient and/or caregiver before the data collection. Those who were unwilling were excluded. Furthermore, no personal identifier was included in the data collection tool, and the data record was kept confidential and used only for the study purpose.

• Field research<br><br>Write "N/A" if the submission does not require an ethics statement.<br><br>General guidance is provided below. Consult the submission guidelines for detailed instructions. **Make sure that all information entered here is included in the Methods section of the manuscript.**	

**Format for specific study types**<br><br>**Human Subject Research (involving human participants and/or tissue)**<br>• Give the name of the institutional review board or ethics committee that approved the study<br>• Include the approval number and/or a statement indicating approval of this research<br>• Indicate the form of consent obtained (written/oral) or the reason that consent was not obtained (e.g. the data were analyzed anonymously)<br><br>**Animal Research (involving vertebrate animals, embryos or tissues)**<br>• Provide the name of the Institutional Animal Care and Use Committee (IACUC) or other relevant ethics board that reviewed the study protocol, and indicate whether they approved this research or granted a formal waiver of ethical approval<br>• Include an approval number if one was obtained<br>• If the study involved *non-human primates*, add *additional details* about animal welfare and steps taken to ameliorate suffering<br>• If anesthesia, euthanasia, or any kind of animal sacrifice is part of the study, include briefly which substances and/or methods were applied<br><br>**Field Research**<br><br>Include the following details if this study involves the collection of plant, animal, or other materials from a natural setting:<br>• Field permit number<br>• Name of the institution or relevant body that granted permission	
**Data Availability**<br><br>Authors are required to make all data underlying the findings described fully available, without restriction, and from the time of publication. PLOS allows rare exceptions to address legal and ethical concerns. See the PLOS Data Policy and FAQ for detailed information.	Yes - all data are fully available without restriction

A Data Availability Statement describing where the data can be found is required at submission. Your answers to this question constitute the Data Availability Statement and **will be published in the article**, if accepted.<br><br>**Important:** Stating 'data available on request from the author' is not sufficient. If your data are only available upon request, select 'No' for the first question and explain your exceptional situation in the text box.<br><br>Do the authors confirm that all data underlying the findings described in their manuscript are fully available without restriction?	
**Describe where the data may be found in full sentences. If you are copying our sample text, replace any instances of XXX with the appropriate details.**<br><br>• If the data are **held or will be held in a public repository**, include URLs, accession numbers or DOIs. If this information will only be available after acceptance, indicate this by ticking the box below. For example: *All XXX files are available from the XXX database (accession number(s) XXX, XXX.)*.<br>• If the data are all contained **within the manuscript and/or Supporting Information files**, enter the following: *All relevant data are within the manuscript and its Supporting Information files.*<br>• If neither of these applies but you are able to provide **details of access elsewhere**, with or without limitations, please do so. For example:<br><br>*Data cannot be shared publicly because of [XXX]. Data are available from the XXX Institutional Data Access / Ethics Committee (contact via XXX) for researchers who meet the criteria for access to confidential data.*<br><br>*The data underlying the results presented in the study are available from (include the name of the third party*	All relevant data are within the paper and its Supporting Information file.

and contact information or URL).<br>• This text is appropriate if the data are owned by a third party and authors do not have permission to share the data.<br><br>* typeset	
Additional data availability information:	Tick here if the URLs/accession numbers/DOIs will be available only after acceptance of the manuscript for publication so that we can ensure their inclusion before publication.

Powered by Editorial Manager® and ProduXion Manager® from Aries Systems Corporation

Manuscript Click here to access/download;Manuscript;Manuscript.docx

Poisoning Cases and Their Management in Amhara Region, Ethiopia: Hospital-Based Prospective Study

Assefa Belay Asrie[1], Seyfe Asrade Atnafie[1], Kefyalew Ayalew Getahun[1], Eshetie Melese Birru[1], Gashaw Binega Mekonnen[2], Geta Asrade Alemayehu[3], Berhanu Fikadie Endehabtu[4], Marta Berta Badi[5], Getinet Mequanint Adinew[1]

[1]Department of Pharmacology, School of Pharmacy, College of Medicine and Health Sciences, University of Gondar, Gondar, Ethiopia

[2]Department of Clinical Pharmacy, School of Pharmacy, College of Medicine and Health Sciences, University of Gondar, Gondar, Ethiopia

[3]Department of Health Service Management and Health Economics, Institute of Public Health, College of Medicine and Health Sciences, University of Gondar, Ethiopia

[4]Department of Health Informatics, Institute of Public Health, University of Gondar, Gondar, Ethiopia

[5]Department of Women’s and Family Health, School of Midwifery, College of Medicine and Health Sciences, University of Gondar, Gondar, Ethiopia

Abstract

Background: Poisoning is a significant public health problem globally. Ethiopia is a low-income country undergoing technological and social change that may increase access to drugs and chemicals, potentially increasing the incidence of poisoning. This study describes the epidemiology of hospital admissions due to poisoning in a region of Ethiopia.

Methods: An institution based prospective observational study was employed, as a study design, in selected hospitals of the region from January~~,~~ 2018 to December~~,~~ 2018.

Results: Of 442 poisoning cases, 78 (17.6%) were died. Almost all poisoning cases were intentional self-poisonings. The most frequent poisonings were organophosphate compounds, 145 (32.8%), and metal phosphides (majorly aluminum phosphide), 115 (26.0%). The ingested poison was most frequently accessed from the patients’ home, 243 (55.0%), followed by purchasing from local shops, 159 (36%). The median duration of admission was 24 hours. Of all the cases, 23 (5.2%) were admitted to intensive care unit (ICU) requiring mechanical ventilation.

Most of the cases admitted to the ICU were aluminum phosphide-poisoned patients. The majority of deaths (43 of 78) were due to metal phosphides. From the multivariate logistic regression analysis, age, altered level of consciousness on hospital arrival, distance from the area where the poisoning incidence happened to the hospital, and no laboratory result as a part of the diagnosis process or investigation of the extent of toxicity were found to be significantly associated with the likelihood of poor treatment outcome.

Conclusion: The majority of the poisoning cases were females. The most common reasons for the intent of self-poisoning were dispute-related, mainly family disharmonies, followed by psychiatric conditions. The poisoning agents were mostly obtained from households. Organophosphate compounds and metal phosphides were the first and the second most frequently encountered poisoning agents, respectively, and it was noted that the later ones were responsible for most of the fatal cases. Of the pharmacologic, atropine was the only agent regarded as antidote. The most commonly employed agent for supportive treatment was cimetidine followed by maintenance fluids, while gastric lavage was the only GI decontamination method used among others. Cimetidine was the most often used agent for supportive treatment, followed by maintenance fluids and gastric lavage was the only GI decontamination techniques that was utilized. The fatality rate of poisoning in this study was found to be much higher than in other similar studies. Impaired consciousness upon hospital arrival, type of poisoning, and no involvement of laboratory investigation were found to significantly associate with the likelihood of death. Generally the results dictate the need for the design and implementation of strategies to create awareness, prevent, and manage poisoning incidences in the community.

Keywords: Poisoning, poisoning agents, organophosphate compounds, metal phosphides, poisoning management, treatment outcome

Background

Intentional self-poisoning is a common cause of emergency room visits and hospital admissions worldwide and a major reason for illness and death in many countries [1]. Poisoning from pesticides, industrial chemicals, pharmaceuticals, chemical products, and natural toxins is a remarkable public health problem worldwide [2]. An estimate of a systematic review that identified information from 108 nations indicates, from 2010 to 2014, there were over 110,000 pesticide self-poisoning deaths every year, or 13.7% of all suicides worldwide [3]. As advances in technological and social developments have led to ease accessibility of most drugs and chemical substances in the society, the actual number of incidences of poisoning can be higher, because most cases of poisoning actually go unreported [4]. Ethiopia is one of the low-income countries that share its considerable burden of poisoning incidences and deaths. Poisoning was reported as the second most common means of attempted suicide in a district of Ethiopia. The authors also reported that the majority of the poisoning cases were women, and strong detergents and rodenticides were the most frequently used poisons [5]. One prospective study done in selected hospitals in western Ethiopia reported that about 7.1% of poisoning cases died [6]. Similarly, another retrospective study reported that 6.6% of the poisoning cases were died [7]. It can be depicted from such reports that poisoning is one of the health problems contributing to the morbidity and mortality burdens in the country.

The types and frequencies of poisoning agents encountered vary considerably across the world and even in different parts of the same country due to socioeconomic and cultural differences. The frequency of poisoning incidences in a given community depends on the availability of poisonous agents, the common occupational activity in that society, and religious and sociocultural influences [8-10]. Household chemicals and prescribed drugs are the most common poisoning agents in the developed world, while agricultural chemicals are the most common poisoning agents in developing countries [8]. More importantly, limited drug and chemical regulatory activities, inadequacy of surveillance systems, poor enforcement, and ease of access to various kinds of drugs or chemicals have been implicated in the higher poisoning prevalence in underdeveloped countries [11, 12].

This study was carried out to determine the frequently encountered poisoning agents, the clinical managements, and the final outcome (as cure or death) and associated factors.

Methods

The study area and period

The Amhara Region is one of the 10 regional states in Ethiopia. There were more than 70 hospitals, 100 health centers, and 700 health stations in the region during the proposal development for this study. Five of the hospitals were referral-level hospitals. Four of the referral hospitals were conveniently selected as the study sites. The hospitals included in the study are University of Gondar Comprehensive Specialized Hospital, Felege Hiwot Referral Hospital, Debre Markos Referral Hospital, and Dessie Referral Hospital. University of Gondar Comprehensive Specialized Hospital is located in Gondar Town, 750 km northwest of the capital city of Ethiopia, Addis Ababa. It serves a catchment population of approximately 5 million people in Central Gondar and the neighboring zones. Felege Hiwot Referral Hospital is found in Bahir Dar City, which is 562 km northwest of Addis Ababa. The hospital serves a catchment population of more than 5 million. Debre Markos Referral Hospital is located in Debre Markos Town, 300 km northwest of Addis Ababa. The hospital serves more than 3.5 million people in East Gojjam Zone and neighboring zones. Dessie Referral Hospital is located in Dessie Town, in the northern of Ethiopia, at a distance of 400 km from Addis Ababa. It provides healthcare services for about 9 million people in the South Wollo Zone of the Amhara region and the surrounding areas. At all sites, the data collection was started on January 1, 2018 and completed in the last week of December 2018 on different specific dates. Nearly a year was needed to recruit the designated number of cases at each site.

Study design and sampling technique

An institution-based prospective observational study was employed~~,~~ as a study design~~,~~ in the selected hospitals in the region. Considering the number of referral hospitals and their distribution in the region, four referral hospitals were conveniently selected for the study. All consecutive cases admitted to the emergency departments of the hospitals~~,~~ during the data collection period~~,~~ were included in the study, regardless of their age and causes of poisoning. The cases that were not willing to participate in the study and from whom adequate data were not obtained were excluded from the study.

Sample size determination

The sample size was determined by using a single population proportion formula considering the following assumptions: prevalence (p) of poisoning cases 50% to get the larger sample size, standard normal distribution value at 95% confidence level of Z/2 = 1.96, and margin of error (w) = 5%. This gives a sample size of 384 patients, as shown below.

$$N = \frac{Z^2 p(1-p)}{w^2} = \frac{(1.96)^2 0.5(1-0.5)}{(0.05)^2} = 384.16 \approx\ 384 \text{ patients}$$

Where, N is the sample size, Z is confidence interval, p is a proportion, and w is the margin of error.

Once the minimum number of sample was obtained in this way, by adding a 15% non-response rate, the total sample size was determined to be 442. Once the sample determined in this way, it was distributed to the four study sites; 110 cases to each of University of Gondar Comprehensive Specialized Hospital and Felege Hiwot Referral Hospital and 111 cases to each of Debre Markos Referral Hospital and Dessie Referral Hospital. Lottery method was used to decide how many cases would be recruited at each site. Since the data was collected until we achieved the predetermined number of subjects in each study site, the total cases targeted are equal to the originally calculated sample size.

The data collection tool, process, and quality control

Data was collected using a structured questionnaire. The questionnaire was designed to collect data regarding the patients' socio-demographic details (sex, age, ethnicity, occupation, educational status, and marital status) and facts related to the poisoning, such as the type of poison used, reasons for deliberate self-poisoning, the clinical state of the patient on presentation at the emergency department, signs and symptoms of poisoning, and the treatment and outcome of the case. At each site, the data were collected by two trained nurses working in the emergency department of the hospital. The data collection process involved filling out the questionnaire during diagnosis by the data collectors and in part by reviewing the patient record char. To ensure the quality of the data collected, the data collectors were trained on the overall study design and objectives, the contents of the questionnaire, and approaching interviewees. Moreover, supervisors were assigned from the study team for periodic monitoring of the data collection process. In the supervision, some filled questionnaires were randomly selected and crosschecked with their respective patient medical records. Furthermore, each filled

questionnaire was checked for its completeness and clarity before received from the data collectors.

Data entry, analysis, and interpretation

The data was entered into SPSS Version 20 and analyzed using statistical tests. The interpretations were done using descriptive statistics: such as percentages and frequency distributions. Furthermore, the impacts of potential predictors of treatment outcomes (cured or died) were estimated using binary logistic regression analysis at a cutoff point, of $p < 0.05$, for statistically significant association and a confidence interval (CI) of 95%. In this regard, multivariate analysis was carried out for predictor variables using backward stepwise multiple logistic regression. The adjusted odds ratio (AOR) and its 95% CI were calculated for each predictor variable. The variables found to be significantly associated with the likelihood of death were considered the risk factors for poor treatment outcomes from poisoning.

Ethics approval and consent to participants

The protocol of the study has been reviewed and approved by the Institutional Ethical Review Board of University of Gondar (Reference №: O/V/P/RCS/05/372/2018). In addition, each hospital administration office was requested by a formal letter from the University of Gondar, College of Medicine and Health Sciences, Research and Community Service Office, and permission was obtained from each hospital to conduct the study. Furthermore, written informed consent was obtained from each patient or caregiver for inclusion in the study. Those who were unwilling were excluded. Furthermore, no personal identifier was included in the data collection tool, and the data record was kept confidential and used only for the study purposes.

Results

Socio-demographic characteristics of the participants

The majority of the patients were under 40 years of age, females, Orthodox Christians, and Amhara in ethnicity. About half of the cases were single in their marital status. A slightly higher percentage of the cases were from rural residential areas. A relatively higher proportion of the patients could not read and write, followed by those at the secondary school level. Occupationally, students and framers were the first- and second-most frequent cases (Table 1).

Table 1. Socio-demographic characteristics of the participants.

Variables		Frequency	%
Sex	Male	163	36.9
	Female	279	63.1
Age	≤20 years	147	33.3
	21-40 years	235	53.2
	>40 years	60	13.6
Religion	Orthodox Christian	363	82.1
	Muslim	77	17.4
	Protestant	2	0.5
Ethnicity	Amhara	434	98.2
	Others	8	1.8
Residence	Rural	216	48.9
	Urban	226	51.1
Marital status	Married	201	45.5
	Single	223	50.5
	Other	18	4.1
Educational status	Cannot read and write	128	29.0
	Can read and write	72	16.3
	Primary school	67	15.2
	Secondary school	116	26.2
	College level	43	9.7
	University level	16	3.6
Occupation	Student	139	31.4
	Farmer	130	29.4
	Self-employee[a]	91	20.6
	House wife	26	5.9
	Government employee	20	4.5
	Daily laborer	18	4.1
	Others	18	4.1

Types of poisoning agents and death distributions

Organophosphate compounds and metal phosphides were the first and the second most commonly encountered causes of poisoning. The total fatality rate was found to be 17.6%. The majority of fatal cases were among metal phosphide (43 of 78) and organophosphate compound (25 of 78) poisoned cases (Table 2).

Table 2. Types/causes of poisoning involved agents and death distributions by poisoning agents and treatment outcomes.

Poisoning agent	Treatment outcome, n (%)*		Total, n (%)*
	Survived	Died	
Organophosphates	120 (27.1)	25 (5.7)	145 (32.8)

Metal phosphides	72 (16.3)	43 (9.7)	115 (26.0)
Bleaching agents	44 (10.0)	0 (0.0)	44 (10.0)
Medicines	31 (7.0)	2 (0.5)	33 (7.5)
Rat poison	22 (5.0)	4 (0.9)	26 (5.9)
Alcohol	20 (4.5)	0 (0.0)	20 (4.5)
Carbon monoxide	14 (3.2)	0 (0.0)	14 (3.2)
Food poisoning	12 (2.7)	0 (0.0)	12 (2.7)
Unknown chemicals	6 (1.4)	1 (0.2)	7 (1.6)
Herbs	5 (1.1)	0 (0.0)	5 (1.1)
Snake bite	5 (1.1)	0 (0.0)	5 (1.1)
Dichlorodiphenyltrichloro ethane (DDT)	4 (0.9)	0 (0.0)	4 (0.9)
Others[#]	9 (2.0)	3 (0.7)	12 (2.7)
Total, n (%)*	364 (82.4)	78 (17.6)	442 (100.0)

[#]Include fertilizer, unspecified herbicides & insecticides, and match head
*The percentages are calculated out of all cases.

Sources of the poisoning agents

Home was reported by most of the poisoned patients as source of poisoning agents (55.0%), followed by shops (36.0%). The other sources reported include pharmacy, veterinary drug vender, work place, fire accident smoke, traditional medical practitioner (Fig. 1).

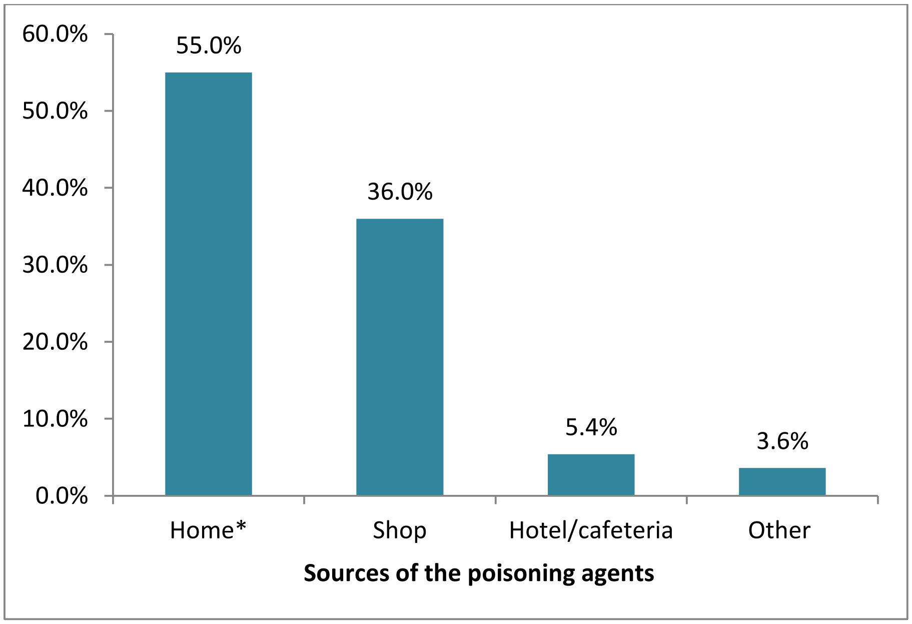

Fig 1. The percentage distribution of the source of poisoning agents encountered.

*Includes homes other than the cases were living in.

Reasons for poisoning incidents

Family dispute (46.4%), psychiatric illnesses (13.1%), and love affairs (10.2%) were the top three frequently reported reasons for self-poisoning. In addition to the specified reasons mentioned, 7.7% of the patients were poisoned for unknown reasons (Table 3).

Table 3. Reported reasons behind the poisoning incidents.

Reason	Frequency	%
Dispute related[a]	205	46.4
Psychiatric disorders	58	13.1
Love affairs	45	10.2
Unknown	34	7.7
Accident	32	7.2
Joblessness	18	4.1
Disease treatment	11	2.5
Recreational purpose	11	2.5
Unplanned pregnancy	8	1.8
Loss of beloved ones	7	1.6
Academic failure	6	1.4
Others	7	1.6

[a]Most dispute related reasons were disputes within families.

Treatments

The sole proven antidote among the treatments mentioned was atropine, which was utilized in 22.4% of the instances. Magnesium sulphate was indicated in 17.6% of the cases. Gastric lavage was the only method used gastrointestinal decontamination method and used in 17.0% of the cases. Maintenance fluids and cimetidine were the first and second most commonly used agents for supportive or symptomatic treatment of the poisoning cases (Table 4). Additionally, of the total cases in this study, 5.2% were admitted to intensive care unit (ICU) and intubated or mechanically ventilated and/or given circulatory support. Most of the cases admitted to the ICU were aluminum phosphide-poisoned patients (data not shown). The patients were admitted to the intensive care unit (ICU) because of serious symptoms, particularly those of respiratory failure

and severe hypotension that necessitated endotracheal intubation or mechanical ventilation and/or circulatory support.

Table 4. Treatments given to the poisoned patients.[♯]

Agents/method		Frequency	%
**Antidotal**	Atropine	99	22.4
	Magnesium sulfate*	78	17.6
**GI decontamination**	Gastric lavage	75	17.0
**Removal enhancer**	Frusemide	28	6.3
**Supportive/symptomatic**	Maintenance fluids	239	54.1
	Cimetidine	267	60.4
	Antibiotic/s	54	12.2
	Hydrocortisone	46	10.4
	Omeprazole	41	9.3
	Metoclopramide	25	5.7
	Dopamine	21	4.8
	Vitamin B complex	16	3.6
	40% glucose	15	3.4
	Calcium gluconate	10	2.3
	Oral rehydration salts (ORS)	8	1.8
	Tramadol	6	1.4
	Antacids	5	1.1
**Admitted to ICU**	Mechanical ventilation and other supportive cares	23	5.2

[♯]Agents with indication frequency of less have been excluded.
*Mechanistically has antidotal activity but not approved yet.

Factors associated with treatment outcome of poisoning emergency

A bivariate analysis was done for each potential variable assumed to be associated with the treatment outcome. The variables that were verified to be significantly associated with treatment outcomes in the bivariate were included in the multivariate logistic regression model. Based on the patients' level of consciousness upon arrival at the emergency departments of the hospitals, those who were in a semiconscious state were 12.145 times more likely to die (AOR: 12.145, 95% CI: 3.686-40.019, $p < 0.001$) than those who were in a conscious state. Moreover, those

who were in an unconscious state at arrival were 38.423 times more likely to die (AOR: 38.423, 95% CI: 10.408-141.848, $p < 0.001$) than those were in a conscious state. An hour increase in the time of hospital stay decreased the likelihood of death by 9.1% ($\beta = -0.096$, AOR: 0.909, 95% CI: 0.882-0.936, $p < 0.001$). Patients poisoned with metal phosphides were 6.983 times (AOR: 6.983, 95% CI: 2.559–19.056, $p < 0.001$) more likely to die than those intoxicated with organophosphate agents. In addition, the odds of death among poisoned patients who had no laboratory result as a part of their diagnosis process or investigation of extent of toxicity was 5.376 times (AOR: 5.376, 95% Cl: 1.633–17.704, $p < 0.01$) than those whose diagnosis involved laboratory tests (Table 5).

Table 5. Multivariate analysis of factors associated with treatment outcome (R^2=0.654, Hosmer and Lemeshow goodness of Fit Test, p=0.924).

Explanatory Variables	β-coefficient	Treatment outcome		AOR (95% C.I.)	p-value
		Cured	Died		
**Age (year)**	0.032	-	-	1.033 (0.997-1.069)	0.071
**Residence**					0.215
Rural	-	169	47	1	
Urban	0.693	195	31	0.657 (0.338-1.275)	
**Level of consciousness[1]**					0.000
Conscious	-	189	19	1	
Semiconscious	2.497	88	19	**12.145 (3.686-40.019)*** **	
Unconscious	3.649	87	40	**38.423 (10.408-141.848)*** **	
**Distance (km)[2]**	0.004	-	-	1.004 (.986-1.022)	0.686
**Time of arrival (hr)[3]**	-0.040	-	-	0.961 (0.890-1.038)	0.312
**Duration of hospital stay**	-0.096	-	-	**0.909 (0.882-0.936)*** **	0.000
**Major poison groups**					0.000
Organophosphates	-			1	
Metal phosphides	1.944			**6.983 (2.559-19.056)*** **	
**Laboratory test**					0.006
Yes	-	111	11	1	
No	**1.682**	253	67	**5.376 (1.633-17.704)** **	

The cut point for significant association is at $p<0.05$.

*p < 0.05, **p < 0.01, ***p < 0.001

[1]Level of consciousness measured just on arrival to the referral hospitals

[2]The distance is from the place of exposure to the referral hospital.

[3]Time of arrival is the time from the time of exposure to arrival to the referral hospitals.

Discussion

This study is dedicated to investigate poisoning in concern with the poisoning patterns in relation to socio-demographic characteristics, the type of poisoning agents involved, treatments given and associated factors with clinical outcomes as death or cure among poisoning cases attended the selected referral hospitals in Amhara Regional State, Ethiopia. The main findings of the study are explained and/or compared with previously published findings of similar studies.

The incidence of poisoning may vary with different categories of socio-demographic characteristics such as age group, sex, residence, and occupation. In this study, the majority of the poisoning cases were females (63.1%). This result is consistent with the results of previous studies in Ethiopia and Taiwan, which reported 59.20% and 54.76% of incidences, respectively, were females [6, 13]. More than half, 53.2%, of the cases were in the age group of 21–40 years, and this is in line with the results of other similar studies [7, 14]. Regarding residence, the poisoning incidences were found to be somewhat greater in urban areas, 51.1% in urban and 48.9% in rural areas. This difference might be, at least in part, because some of the cases from rural areas may not attend hospitals as they may be treated in health posts, health centers, or district hospitals in their locality. Considering the distribution of the cases by marital status, 50.5% of the poisoned patients were single. This proportion is lower than reports of similar studies in Addis Ababa, Ethiopia and Selected Hospitals in Western Ethiopia, which are 54.98% and 57.10%, respectively [6, 15]. The largest proportion of the poisoning cases were students (31.4%), followed by farmers (29.4%) in occupation. This may be due to the fact that students are prone to emotional liability associated with academic failure and love-related issues leading to intentional poisoning for suicidal purpose.

Regarding the poisoning agents involved, organophosphate compounds~~,~~ and metal phosphides (mainly aluminum phosphide)~~,~~ were the first and the second most commonly reported causes of poisons, followed by other agents such as bleaching agents, medicines, unknown chemicals regarded as rat poison, alcohol, and carbon monoxide in decreasing order of frequency. Concerning organophosphates, this result is consistent with the findings of other studies reporting these compounds as the most frequently encountered poisoning agents [14, 16-18], but

inconsistent with another study at Jimma University Specialized Hospital [10]. In Ethiopia organophosphate compounds and aluminum phosphide are widely available as pesticides. This may increase the availability and ease their accessibility for intentional poisoning.

Concerning the sources of the poisoning agents, the majority were obtained at residential homes and local shops. This result is in agreement with the results of other studies in that most of the poisoning agents were obtained at home [7, 15]. This could be because of the inappropriate storage of the poisoning agents at home, when leftover from their use as insecticides or against grain storage pastes. Shops were found to be the second most frequently reported source of poisoning agents, and this result is consistent with the result reported by a similar study carried out in western Ethiopia [9]. The most common reasons for intentional poisoning were dispute-related, mainly family disharmonies, followed by psychiatric conditions, love affairs, and unidentified causes, among others. The reasons reported in this study are similar to those in a systematic review by Chelkeba *et al* [19]. Furthermore, regarding the most frequently reported reason for poisoning, the result of this study is in line with other previous studies [16, 20].

Various interventions were used to treat the poisoning cases. The sole proven antidote among the pharmacologic treatments mentioned was atropine, known to be an antidote to organophosphate poisoning. Magnesium sulfate has been reported to have beneficial effects on aluminum phosphide and organophosphate poisonings [21, 22]. Mechanistically, it has antidotal activities, especially against organophosphate poisoning [21]. However, it has not been approved as an antidote for either organophosphate or aluminum phosphide poisoning (no literature search results show its approval). The remaining pharmacologic agents are used more for supportive treatment of poison-related symptoms rather than for antidotal effects. The most commonly employed nonpharmacologic interventions were supportive care (ABC, mainly IV resuscitation), and this finding is favorable as ensuring a protected airway, adequate ventilation, and hemodynamic stability through the ABC-approach is crucial in the management of poisoning emergencies [19].

Gastric lavage was the only GI decontamination method used in managing the poisoning. Gastric lavage is indicated for immediate removal of stomach contents after an orally ingested overdose or poisoning [23]. The use of this method in about 17.0% of the cases~~,~~ in this study~~,~~ was most

probably from this perspective. However, it is not recommended for routine use and should not be considered unless a patient has ingested a potentially life-threatening amount of a poison [24]. It should be considered only when there is a risk of significant toxicity or imminent fatality, and in situations where antidotal or other supportive modalities are inadequate, and after contraindications are ruled out [23]. Because its efficiency in removing the poisoning agents was found to be highly variable and diminished with time in experimental studies and clinical studies, it showed limited beneficial effects. Moreover, the procedure is associated with serious risks, including hypoxia, dysrhythmias, perforation of the GI tract or pharynx, aspiration pneumonitis, and others [25]. Therefore, consideration should first be given to other less invasive modalities of gastrointestinal decontamination, such as activated charcoal or whole-bowel irrigation [23]. However, there was no report of using other GI decontamination agents or methods such as activated charcoal and whole bowel irrigation.. Interestingly, among all the cases, dialysis was used only in one case. This might be because of unavailability of the facility or unaffordability of the procedure, but not because cases needing the intervention were rare.

The case fatality rate from acute poisoning was found to be 17.6%. This finding is much higher than previous findings of similar studies carried out in Ethiopia [6, 9, 13, 15, 26] and India [14], ranging 4.10-10.20%. Out of 78 deaths, metal phosphides were responsible for 55.1% of the deaths, whereas organophosphates were attributed to 32.1% of the deaths. Even though bleaching agents were the third most common cause of poisoning, no death was recorded among the victims poisoned by these agents. Organophosphates, aluminum phosphide, and other chemicals are available in Ethiopia for use as rodenticide, insecticide, or herbicide [27]. However, a comparative study showed the Ethiopian officers who handle these chemicals are less knowledgeable about the products [28]. Aluminum phosphide is widely available in Ethiopia with approved use for the control of maize weevils and flour beetles on stored maize [27]. Organophosphate compounds, especially malathion, are also among the commonly available pesticides for agricultural uses [28]. Because of their wide availability and ease of accessibility, plus their inherent toxicity, these agents are more likely to pose a great health burden to the community. This result may dictate the need for the design and implementation of strategies to create awareness about and prevent poisonings by such frequently encountered agents in the community.

Multivariate logistic regression analysis was done to identify predictors of treatment outcome in the present study. Accordingly, level of consciousness upon hospital arrival, length of hospitalization, type of poisoning agents encountered, and use of laboratory test as a part of diagnosis were identified as significant potential predictors of treatment outcome. Based on the level of consciousness, the patients who were in semiconscious and unconscious states upon hospital arrival were 12.145 and 38.423 times more likely to die, respectively, than those who were conscious. The level of consciousness may be compromised due to a delay in intervention or because of the inherent toxic characteristics of a poisoning agent, leading to rapid loss of consciousness and negatively affecting the treatment outcome. On the other hand, the analysis showed that as the length of hospitalization increased the probability of death was decreased. This could be the case because the likelihood of death decreased once the patients managed to overcome acute symptoms and received care after being admitted to the hospitals. There was no discernible relationship between distance and treatment outcome. It makes sense that the treatment outcome would be poor consistently the further a patient traveled to get to the hospital. The findings of the present study in this regard could be the consequence of pre-hospital interventions carried out at home, or in community health centers, or smaller general hospitals, which were not taken into account in this study. Patients poisoned with metal phosphides were 6.983 times more likely to die ($p < 0.001$) than those intoxicated with organophosphate agents. Moreover, 43 of 78 the fatal cases were among metal phosphide-poisoned patients. This could indicate that metal phosphide poisoning is more severe and should be considered as a great public health threat in the community. The likelihood of death among poisoned patients who had no laboratory result as a part of their diagnosis process was 5.376 times than that of those whose diagnosis involved laboratory tests. This might be because the laboratory diagnosis can help investigate the severity of toxicity and direct treatment intervention accordingly.

The prospective nature of the study design enables us to collect detail clinical and laboratory data to determine the severity of the cases as a determinant of treatment outcome. This can be taken as the strength of this study. However, the study lacks some parameters including pre-hospital interventions (that might be given at home or local health facilities), referral cases, and appropriateness of interventions. The absence of such variables can be considered a limitation of this study. Especially, the result of this study may be affected by referral bias. If these variables were included, it would be better for a more appropriate explanation of potential predictor

variables of the treatment outcome. For example, the likelihood of death was not significantly increased with time of delay for hospital arrival, and this might be because of pre-hospital interventions at home or local health centers. It does not indicate that time of delay to come after intoxication has no negative impact on treatment outcome. Similarly, the inconsistent association between the distances from the areas where the poisoning incidence occurred to the hospitals would be more objectively explained if the possible pre-hospital interventions and referral cases were considered.

Conclusion

The majority of the poisoning cases were females. The most common reasons for the intent of self-poisoning were dispute-related, mainly family disharmonies, followed by psychiatric conditions. The poisoning agents were mostly obtained from households. Organophosphate compounds and metal phosphides were the first and the second most frequently encountered poisoning agents, respectively, and it was noted that the later ones were responsible for most of the fatal cases. Of the pharmacologic, atropine was the only agent regarded as antidote. The most commonly employed agent for supportive treatment was cimetidine followed by maintenance fluids, while gastric lavage was the only GI decontamination method used among others. Cimetidine was the most often used agent for supportive treatment, followed by maintenance fluids and gastric lavage was the only GI decontamination techniques that was utilized. The fatality rate of poisoning in this study was found to be much higher than in other similar studies. Impaired consciousness upon hospital arrival, type of poisoning, and no involvement of laboratory investigation were found to significantly associate with the likelihood of death. Generally the results dictate the need for the design and implementation of strategies to create awareness, prevent, and manage poisoning incidences in the community.

Availability of data

The data set of the study is available online and can be accessed without any restriction.

Authors' contributions

All authors were participated in the conception, design, and development of the proposal of the study for grand competition and in supervision of the data collection process. GMA had substantial contribution in the preparation of the data collection tool. ABA accomplished the data entry, analysis, interpretation, and the writing up of the manuscript. Finally, all authors read and

approved the submission of the manuscript for publication. Moreover, all of the authors are responsible regarding the appropriateness and originally of this work.

Acknowledgements

We are grateful to the administration offices of the hospitals for their cooperation in facilitating the official process of getting permission to undertake the study in their respective institutions. Besides, we are thankful to the physicians and nurses who were working in the emergency departments of the hospitals for their unwavering cooperation in the data collection process.

Competing interests

The authors have declared that no competing interests exist.

Funding

This study was done using a grant awarded by University of Gondar, Ethiopia (Reference №: R/T/T/C/Eng.58/03/2018). The funder had no role in the study design, data collection and analysis, decision to publish, or preparation of the manuscript.

References

1. G/Mariam ET, Gelaw BK. Global Epidemiology of Acute Poisoning with an Emphasis to Ethiopia: Systematic Review. *Int J Pharm Sci & Scient Res*. 2016; 2(4): 120-130.
2. WHO. 2021. Chemical Safety and Health: Prevention and management of cases of poisoning. Accessed on November 12, 2022. Available on https://www.who.int/teams/environment-climate-change-and-health/chemical-safety-and-health/incidents-poisonings/prevention-and-management-of-cases-of-poisoning
3. Mew EJ, Padmanathan P, Konradsen F, Eddleston M, Chang SS, Phillips MR, Gunnell D. The global burden of fatal self-poisoning with pesticides 2006-15: Systematic review. *J Affect Disord*. 2017;219:93-104.
4. Abd-Elhaleem ZAE, Muqhem BAA. Pattern of acute poisoning in Al Majmaah region, Saudi Arabia. *Am J Clin Exp Med*. 2014; 2(4): 79–85.
5. Kebede D, Alem A. Suicide attempts and ideation among adults in Addis Ababa, Ethiopia. *Acta Psychiatr Scand Suppl*. 1999; 397:35-39.

6. Woyessa AH, Palanichamy T. Patterns, Associated Factors, and Clinical Outcomes of Poisoning among Poisoning Cases Presented to Selected Hospitals in Western Ethiopia: Hospital-Based Study. *Emerg Med Int*. 2020; 2020:5741692.
7. Getie A, Belayneh YM. A Retrospective Study of Acute Poisoning Cases and Their Management at Emergency Department of Dessie Referral Hospital, Northeast Ethiopia. *Drug Healthc Patient Saf*. 2020;12:41-48.
8. Teklemariam E, Tesema S, Jemal A. Pattern of acute poisoning in Jimma University Specialized Hospital, South West Ethiopia. World J Emerg Med. 2016;7(4):290-293.
9. Singh S, Sharma BK, Wahi PL, Anand BS, Chugh KS. Spectrum of acute poisoning in adults (10 year experience). *J Assoc Physicians India*. 1984 Jul;32(7):561-3. PMID: 6501193.
10. Padmanabha TS, Gumma K, Kulkarni GP. Study of profile of organophosphorus poisoning cases in a tertiary care hospital, North Karnataka, Bidar, India. *Int J Pharm Bio Sci*. 2014;5(1):332-339.
11. Kumar S, Pathak A, Mangal HM. Trends of fatal poisoning in Saurashtra region of Gujarat (a prospective study). *Journal of Indian Academy of Forensic Medicine*. 2011; 33(3):197-199.
12. Somasundaram KV, Patil A, Shukla SK. Epidemiological profile of OP poisoning cases treated at Pravara hospital, Loni, India. *Indian J Prev Soc Med*. 2009;40(3&4):184-188.
13. Lee HL, Lin HJ, Yeh ST, Chi CH, Guo HR. Presentations of patients of poisoning and predictors of poisoning-related fatality: findings from a hospital-based prospective study. *BMC Public Health*. 2008;8:7.
14. Kumar SV, Venkateswarlu B, Sasikala M, Kumar GV. A study on poisoning cases in a tertiary care hospital. *Journal of natural science, biology, and medicine*. 2010;1(1):35.
15. Zemedie B, Sultan M, Zewdie A. Acute Poisoning Cases Presented to the Addis Ababa Burn, Emergency, and Trauma Hospital Emergency Department, Addis Ababa, Ethiopia: A Cross-Sectional Study. *Emerg Med Int*. 202; 2021:6028123.
16. Adinew GM, Woredekal AT, DeVos EL, Birru EM, Abdulwahib MB. Poisoning cases and their management in emergency centres of government hospitals in northwest Ethiopia. *Afr J Emerg Med*. 2017;7(2):74-78.
17. Jamie AH, Abdosh MZ. Patterns and Clinical Outcomes of Poisoning Among Poisoning Cases Presented in a Hospital: A Retrospective Study. *Lokman Hekim Health Sci*. 2021;1(3):70–73.

18. Ramesha KN, Rao KB, Kumar GS. Pattern and outcome of acute poisoning cases in a tertiary care hospital in Karnataka, India. *Indian J Crit Care Med*. 2009;13(3):152-155.
19. Chelkeba L, Mulatu A, Feyissa D, Bekele F, Tesfaye BT. Patterns and epidemiology of acute poisoning in Ethiopia: systematic review of observational studies. *Arch Public Health*. 2018 Jul 2;76:34.
20. Adinew GM, Asrie AB, Birru EM. Pattern of acute organophosphorus poisoning at University of Gondar Teaching Hospital, Northwest Ethiopia. BMC Res Notes. 2017;10(1):149.
21. Eddleston M, Buckley NA, Eyer P, Dawson AH. Management of acute organophosphorus pesticide poisoning. The Lancet. 2008 ;371(9612):597-607.
22. Karimani A, Mohammadpour AH, Zirak MR, Rezaee R, Megarbane B, Tsatsakis A, Karimi G. Antidotes for aluminum phosphide poisoning–An update. Toxicology reports. 2018 1;5:1053-1059.
23. Jenny J. Lu. Gastric Lavage. Emergency Medicine Procedures., edited by Eric F. Reichman, McGraw-Hill Education, 2013, 393-398
24. Vale JA. Position statement: gastric lavage. American Academy of Clinical Toxicology; European Association of Poisons Centres and Clinical Toxicologists. *J Toxicol Clin Toxicol*. 1997;35(7):711-719.
25. Benson BE, Hoppy K, Troutman WG, Bedry R, Erdman A, Höjer J, *et al*. American Academy of Clinical Toxicology; European Association of Poisons Centres and Clinical Toxicologists. Position paper update: gastric lavage for gastrointestinal decontamination. *Clin Toxicol (Phila)*. 2013;51(3):140-146.
26. Tefera GM, Teferi LG. Prevalence, Predictors and Treatment Outcome of Acute Poisoning in Western Ethiopia. *Open Access Emerg Med*. 2020;12:365-375.
27. Ethiopian Ministry of Agriculture. List of registered pesticides. Accessed on July 17, 2022. http://www.moa.gov.et/list-of-registered-pesticides
28. Tessema RA, Nagy K, Ádám B. Pesticide Use, Perceived Health Risks and Management in Ethiopia and in Hungary: A Comparative Analysis. *Int J Environ Res Public Health*. 2021;18(19):10431.

Supporting Information

Click here to access/download

Supporting Information

Sopporting Information.sav

Poisoning Cases and Their Management in Amhara Region, Ethiopia: Hospital-Based Prospective Study

Assefa Belay Asrie[1], Seyfe Asrade Atnafie[1], Kefyalew Ayalew Getahun[1], Eshetie Melese Birru[1], Gashaw Binega Mekonnen[2], Geta Asrade Alemayehu[3], Berhanu Fikadie Endehabtu[4], Marta Berta Badi[5], Getinet Mequanint Adinew[1]

[1]Department of Pharmacology, School of Pharmacy, College of Medicine and Health Sciences, University of Gondar, Gondar, Ethiopia

[2]Department of Clinical Pharmacy, School of Pharmacy, College of Medicine and Health Sciences, University of Gondar, Gondar, Ethiopia

[3]Department of Health Service Management and Health Economics, Institute of Public Health, College of Medicine and Health Sciences, University of Gondar, Ethiopia

[4]Department of Health Informatics, Institute of Public Health, University of Gondar, Gondar, Ethiopia

[5]Department of Women's and Family Health, School of Midwifery, College of Medicine and Health Sciences, University of Gondar, Gondar, Ethiopia

Abstract

Background: Poisoning is a significant public health problem globally. Ethiopia is a low-income country undergoing technological and social change that may increase access to drugs and chemicals, potentially increasing the incidence of poisoning. This study describes the epidemiology of hospital admissions due to poisoning in a region of Ethiopia.

Methods: An institution based prospective observational study was employed, as a study design, in selected hospitals of the region from January, 2018 to December, 2018.

Results: Of 442 poisoning cases, 78 (17.6%) were died. Almost all poisoning cases were intentional self-poisonings. The most frequent poisonings were organophosphate compounds, 145 (32.8%), and metal phosphides (majorly aluminum phosphide), 115 (26.0%). The ingested poison was most frequently accessed from the patients' home, 243 (55.0%), followed by purchasing from local shops, 159 (36%). The median duration of admission was 24 hours. Of all the cases, 23 (5.2%) were admitted to intensive care unit (ICU) requiring mechanical ventilation.

Most of the cases admitted to the ICU were aluminum phosphide-poisoned patients. The majority of deaths (43 of 78) were due to metal phosphides. From the multivariate logistic regression analysis, age, altered level of consciousness on hospital arrival, distance from the area where the poisoning incidence happened to the hospital, and no laboratory result as a part of the diagnosis process or investigation of the extent of toxicity were found to be significantly associated with the likelihood of poor treatment outcome ~~or death~~.

Conclusion: The majority of the poisoning cases were females. The most common reasons for the intent of self-poisoning were dispute-related, mainly family disharmonies, followed by psychiatric conditions. The poisoning agents were mostly obtained from households. Organophosphate compounds and metal phosphides were the first and the second most frequently encountered poisoning agents, respectively, and it was noted that the later ones were responsible for most of the fatal cases. Of the pharmacologic, atropine was the only agent regarded as antidote. The most commonly employed agent for supportive treatment was cimetidine followed by maintenance fluids, while gastric lavage was the only GI decontamination method used among others. ~~Cimetidine was the most often used agent for supportive treatment, followed by maintenance fluids and gastric lavage was the only GI decontamination techniques that was utilized.~~ The fatality rate of poisoning in this study was found to be much higher than in other similar studies. Impaired consciousness upon hospital arrival, type of poisoning, and no involvement of laboratory investigation were found to significantly associate with the likelihood of death. Generally the results dictate the need for the design and implementation of strategies to create awareness, ~~about and~~ prevent, and manage poisoning~~s~~ incidences ~~by such frequently encountered agents~~ in the community. ~~Impaired consciousness upon hospital arrival, type of poisoning, and no involvement of laboratory investigation were found to significantly associate with the likelihood of death.~~

Keywords: Poisoning, poisoning agents, organophosphate compounds, metal phosphides, poisoning management, treatment outcome

Background

Intentional self-poisoning is a common cause of emergency room visits and hospital admissions worldwide and a major reason for illness and death in many countries [1]. Poisoning from pesticides, industrial chemicals, pharmaceuticals, chemical products, and natural toxins is a remarkable public health problem worldwide [2]. An estimate of a systematic review that identified information from 108 nations indicates, from 2010 to 2014, there were over 110,000 pesticide self-poisoning deaths every year, or 13.7% of all suicides worldwide [3]. As ~~the~~ advances in technological and social developments have led to ease accessibility of most drugs and chemical substances in the society, the actual number of incidences of poisoning can be higher, because most cases of poisoning actually go unreported [4]. Ethiopia is one of the low-income countries that share its considerable burden of poisoning incidences and deaths. Poisoning was reported as the second most common means of attempted suicide in a district of Ethiopia. The authors also reported that the majority of the poisoning cases were women, and strong detergents and rodenticides were the most frequently used poisons [5]. One prospective study done in selected hospitals in western Ethiopia reported that about 7.1% of poisoning cases ~~were~~ died [6]. Similarly, another retrospective study reported that 6.6% of the poisoning cases ~~were~~ died [7]. It can be depicted from such reports that poisoning is one of the health problems contributing to the morbidity and mortality burdens in the country.

The types and frequencies of ~~encountered~~ poisoning agents encountered vary considerably across the world and even in different parts of the same country due to socioeconomic and cultural differences. The frequency of poisoning incidences in a given community depends on the availability of poisonous agents, the common occupational activity in that society, and religious and sociocultural influences [8-10]. Household chemicals and prescribed drugs are the most common poisoning agents in the developed world, while agricultural chemicals are the most common poisoning agents in ~~the~~ developing countries [8]. More importantly, limited drug and chemical regulatory activities, inadequacy of surveillance systems, poor enforcement, and ease of access to various kinds of drugs or chemicals have been implicated ~~for~~ in the higher poisoning prevalence in underdeveloped countries [11, 12].

This study was carried out to determine the frequently encountered poisoning agents, the clinical managements, and the final outcome (as cure or death) and associated factors.

Methods

The study area and period

~~The study was conducted in four referral hospitals in Amhara Region from January 2018 to December 2018.~~ The Amhara Region is one of the 10 ~~Regional States~~ regional states in Ethiopia. There were more than 70 hospitals, 100 health centers, and 700 health stations in the region during the proposal development for this study. Five of the hospitals were referral-level hospitals. Four of the referral hospitals were conveniently selected as the study sites. The hospitals included in the study are University of Gondar Comprehensive Specialized Hospital, Felege Hiwot Referral Hospital, Debre Markos Referral Hospital, and Dessie Referral Hospital. University of Gondar Comprehensive Specialized Hospital is located in Gondar Town, 750 km northwest of the capital city of Ethiopia, Addis Ababa. It serves a catchment population of approximately 5 million people in Central Gondar and the neighboring zones. Felege Hiwot Referral Hospital is found in Bahir Dar City, which is 562 km northwest of Addis Ababa. The hospital serves a catchment population of more than 5 million. Debre Markos Referral Hospital is located in Debre Markos Town, 300 km northwest of Addis Ababa. The hospital serves more than 3.5 million people in East Gojjam Zone and neighboring zones. Dessie Referral Hospital is ~~found~~ located in Dessie Town, in the northern of Ethiopia, at a distance of 400 km from Addis Ababa. It provides healthcare services for about 9 million ~~catchment population~~ people in the South Wollo Zone of the Amhara region and the surrounding areas. At all sites, the data collection was started on January 1, 2018 and completed in the last week of December 2018 on different specific dates. Nearly a year was needed to recruit the designated number of cases at each site,

Study design and sampling technique

An institution-based prospective observational study was employed~~, ~~as a study design~~,~~ in the selected hospitals ~~of~~ in the region. Considering the number of referral hospitals and their distribution in the region, four referral hospitals were conveniently selected for the study. All ~~poisoning~~ consecutive cases admitted to the emergency departments of the hospitals~~,~~ during the data collection period~~,~~ were included in the study, regardless of their age and causes of poisoning. The cases that were not willing to participate in the study and from whom adequate data were not obtained ~~had been~~ were excluded from the study.

Sample size determination

The sample size was determined by using a single population proportion formula considering the following assumptions: prevalence (p) of poisoning cases 50% to get the larger sample size, standard normal distribution value at 95% confidence level of Z/2 = 1.96, and margin of error (w) = 5%. This gives a sample size of 384 patients, as shown below.

$$N = \frac{Z^2 p(1-p)}{w^2} = \frac{(1.96)^2 0.5(1-0.5)}{(0.05)^2} = 384.16 \approx 384 \text{ patients}$$

Where, N is the sample size, Z is confidence interval, p is a proportion, and w is the margin of error.

Once the minimum number of sample was obtained in this way, by adding a 15% non-response rate, the total sample size was determined to be 442. ~~This whole sample was distributed for the four hospitals.~~ Once the sample determined in this way, it was distributed to the four study sites; 110 cases to each of University of Gondar Comprehensive Specialized Hospital and Felege Hiwot Referral Hospital and 111 cases to each of Debre Markos Referral Hospital and Dessie Referral Hospital. Lottery method was used to decide how many cases would be recruited at each site. Since the data was collected until we achieved the predetermined number of subjects in each study site, the total cases targeted are equal to the originally calculated sample size.

The data collection tool, process, and quality control

Data was collected ~~by~~ using a structured questionnaire. The questionnaire was designed to collect data regarding the patients' socio-demographic details (sex, age, ethnicity, occupation, educational status, and marital status) and facts related to the poisoning, such as the type of poison used, reasons for deliberate self-poisoning, the clinical state of the patient on presentation at the emergency department, signs and symptoms of poisoning, and the treatment and outcome of the case. ~~In~~ At each site, the data were collected by two trained nurses working in the emergency department of the hospital. The data collection process ~~was by~~ involved filling out the questionnaire during diagnosis by the ~~attendant physician~~ data collectors and in part by reviewing the patient record char. To ensure the quality of the data collected, the data collectors were trained on the overall study design and objectives, the contents of the questionnaire, and approaching interviewees. Moreover, supervisors were assigned from the study team for periodic monitoring of the data collection process. In the supervision, some filled questionnaires were randomly selected and crosschecked with their respective patient medical records. Furthermore,

each filled questionnaire was checked for its completeness and clarity before received from the data collectors.

Data entry, analysis, and interpretation

The data was entered ~~in to~~ into SPSS Version 20 and analyzed using statistical tests. The interpretations were done using descriptive statistics~~:~~ such as percentages and frequency distributions. Furthermore, the impacts of potential predictors of treatment outcomes (cured or died) were estimated using binary logistic regression analysis at a cutoff point~~,~~ of $p < 0.05$~~,~~ for statistically significant association and a confidence interval (CI) of 95%. In this regard, multivariate analysis was carried out for predictor variables using backward stepwise multiple logistic regression. The ~~A~~adjusted odds ratio (AOR) and its 95% CI ~~was~~ were calculated for each predictor variable. The variables found to be significantly associated with the likelihood of death were considered ~~as~~ the risk factors for poor treatment outcomes ~~of~~ from poisoning.

Ethics approval and consent to participants

The protocol of the study has been reviewed and approved by the Institutional Ethical Review Board of University of Gondar (Reference №: O/V/P/RCS/05/372/2018). In addition, each hospital administration office was requested by a formal letter from the University of Gondar, College of Medicine and Health Sciences, Research and Community Service Office, and permission was obtained from each hospital to conduct the study. Furthermore, ~~participant~~ written informed consent ~~to participate in the study~~ was obtained from each patient ~~and/~~or caregiver ~~before the data collection~~ for inclusion in the study. Those who were unwilling were excluded. Furthermore, no personal identifier was included in the data collection tool, and the data record was kept confidential and used only for the study purposes.

Results

Socio-demographic characteristics of the participants

The majority of the patients were under 40 years of age, females, Orthodox Christians, and Amhara in ~~Ethnicity~~ ethnicity. About half of the cases were single in their marital status. A ~~S~~slightly higher percentage of the cases ~~was~~ were from rural residential areas. A relatively higher proportion of the patients could not read and write, followed by those at the secondary school level. Occupationally, students and framers were the first- and second-most frequent cases (Table 1).

Table 1. Socio-demographic characteristics of the participants.

Variables		Frequency	%
Sex	Male	163	36.9
	Female	279	63.1
Age	≤20 years	147	33.3
	21-40 years	235	53.2
	>40 years	60	13.6
Religion	Orthodox Christian	363	82.1
	Muslim	77	17.4
	Protestant	2	0.5
Ethnicity	Amhara	434	98.2
	Others	8	1.8
Residence	Rural	216	48.9
	Urban	226	51.1
Marital status	Married	201	45.5
	Single	223	50.5
	Other	18	4.1
Educational status	Cannot read and write	128	29.0
	Can read and write	72	16.3
	Primary school	67	15.2
	Secondary school	116	26.2
	College level	43	9.7
	University level	16	3.6
Occupation	Student	139	31.4
	Farmer	130	29.4
	Self-employee[a]	91	20.6
	House wife	26	5.9
	Government employee	20	4.5
	Daily laborer	18	4.1
	Others	18	4.1

~~Distances from the places of poisoning incidences and the time of arrival to the referral hospitals~~

~~More than half of the poisoning cases came from areas farther away than 10 km from the referral hospitals they attended and were arrived after 2 hours of the poisoning incidences (Table 2).~~

~~Table 2. Distance from the place of poisoning incidences and time of arrival to the hospitals after poisoning.~~

~~Distance from place of poisoning incidence to hospitals (km)~~	~~Time of arrival to hospital, n(%)~~		~~Total, n(%)~~
	~~Within 2 hours~~	~~After 2 hours~~	
~~≤10~~	~~142(32.1)~~	~~51(11.5)~~	~~193 (43.7)~~
~~11-20~~	~~21(4.8)~~	~~30(6.8)~~	~~51(11.5)~~
~~21-30~~	~~12(2.7)~~	~~56(12.7)~~	~~68 (15.4)~~
~~31-40~~	~~3(0.7)~~	~~33(7.5)~~	~~36 (8.1)~~

~~>40~~	~~4(0.9)~~	~~90(20.4)~~	~~94 (21.3)~~
~~Total (%)~~	~~182 (41.2)~~	~~260 (58.8)~~	~~442 (100.0)~~

Types of poisoning agents and death distributions

Organophosphate compounds and metal phosphides were the first and the second most commonly encountered causes of poisoning. The total fatality rate was found to be 17.6%. The majority of fatal cases were among metal phosphide (43 of 78) and organophosphate compound (25 of 78) poisoned cases (Table ~~3~~ 2).

Table ~~3~~ 2. Types/causes of poisoning involved agents and death distributions by poisoning agents and treatment outcomes.

Poisoning agent	Treatment outcome, n (%)*		Total, n (%)*
	~~Died~~ Survived	~~Cured~~ Died	
Organophosphates	120 (27.1)	25 (5.7)	145 (32.8)
Metal phosphides	72 (16.3)	43 (9.7)	115 (26.0)
Bleaching agents	44 (10.0)	0 (0.0)	44 (10.0)
Medicines	31 (7.0)	2 (0.5)	33 (7.5)
Rat poison	22 (5.0)	4 (0.9)	26 (5.9)
Alcohol	20 (4.5)	0 (0.0)	20 (4.5)
Carbon monoxide	14 (3.2)	0 (0.0)	14 (3.2)
Food poisoning	12 (2.7)	0 (0.0)	12 (2.7)
Unknown chemicals	6 (1.4)	1 (0.2)	7 (1.6)
Herbs	5 (1.1)	0 (0.0)	5 (1.1)
Snake bite	5 (1.1)	0 (0.0)	5 (1.1)
Dichlorodiphenyltrichloroethane (DDT)	4 (0.9)	0 (0.0)	4 (0.9)
Others#	9 (2.0)	3 (0.7)	12 (2.7)
Total, n (%)*	364 (82.4)	78 (17.6)	442 (100.0)

#Include fertilizer, unspecified herbicides & insecticides, and match head

*The percentages are calculated out of all cases.

~~**Dosage form of the poisons encountered and the routes of exposure**~~

~~Almost all of the poisoning agents were either in solid (49.1%) or liquid (46.3%) dosage forms. The majority of the cases were poisoned through the oral route of exposure (93.7%). The liquid~~

dosage forms with other routes of exposure were snake venoms from five snake bite accidents and one topical exposure of an organophosphate compound (Table 4).

Table 4. Dosage forms of the poisons involved and the routes of exposure.

Dosage form of the poison	Route of exposure			Total, n (%)
	Oral	Inhalational	Other	
Solid	216	1	0	217 (49.1%
Liquid	198	0	6	204 (46.2%)
Gas	0	19	1	20 (4.5)
Other#	0	0	1	1 (0.2%)
**Total, n (%)**	414 (93.7%)	20 (4.5%)	8 (1.8%)	442 (100.0%)

#Semisolid herbal preparation of topical exposure
Percentages calculated out of 442 cases.

Sources of the poisoning agents

Home was reported by most of the poisoned patients as source of poisoning agents (55.0%), followed by shops (36.0%). The other sources reported include pharmacy, veterinary drug vender, work place, fire accident smoke, traditional medical practitioner (Fig. 1).

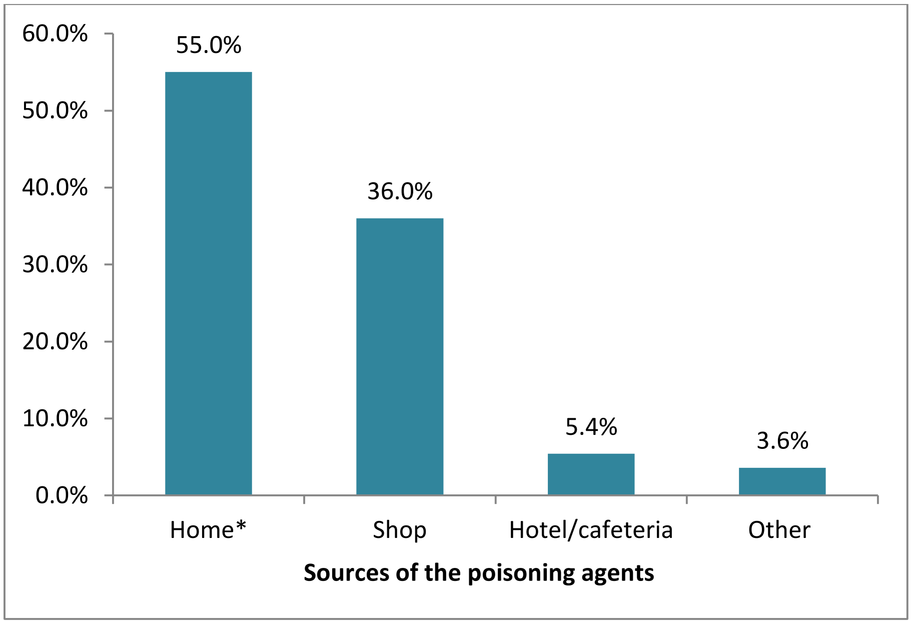

Fig 1. The percentage distribution of the source of poisoning agents encountered.

*Includes homes other than the cases were living in.

Reasons for poisoning incidents

Family dispute (46.4%), psychiatric illnesses (13.1%), and love affairs (10.2%) were the top three frequently reported reasons for self-poisoning. In addition to the specified reasons mentioned, 7.7% of the patients were poisoned for unknown reasons (Table ~~5~~ 3).

Table ~~5~~ 3. Reported reasons behind the poisoning incidents.

Reason	Frequency	%
Dispute related[a]	205	46.4
Psychiatric disorders	58	13.1
Love affairs	45	10.2
Unknown	34	7.7
Accident	32	7.2
Joblessness	18	4.1
Disease treatment	11	2.5
Recreational purpose	11	2.5
Unplanned pregnancy	8	1.8
Loss of beloved ones	7	1.6
Academic failure	6	1.4
Others	7	1.6

[a]Most dispute related reasons were disputes within families.

Treatments

The sole proven antidote among the treatments mentioned was atropine, which was utilized in 22.4% of the instances. Magnesium sulphate was indicated in 17.6% of the cases. Gastric lavage was the only method used gastrointestinal decontamination method and used in 17.0% of the cases. Maintenance fluids and cimetidine were the first and second most commonly used agents for supportive or symptomatic treatment of the poisoning cases (Table ~~6~~ 4). Additionally, of the total cases in this study, 5.2% were admitted to intensive care unit (ICU) and intubated or mechanically ventilated and/or given circulatory support ~~(data not shown)~~. Most of the cases admitted to the ICU were aluminum phosphide-poisoned patients (data not shown). The patients were admitted to the intensive care unit (ICU) because of serious symptoms, particularly those of respiratory failure and severe hypotension that necessitated endotracheal intubation or mechanical ventilation and/or circulatory support.

Table ~~6~~ 4. Treatments given to the poisoned patients.[♯]

Agents/method		Frequency	%
**Antidotal**	Atropine	99	22.4
	Magnesium sul~~ph~~fate*	78	17.6
**GI decontamination**	Gastric lavage	75	17.0
**Removal enhancer**	Frusemide	28	6.3
**Supportive/symptomatic**	Maintenance fluids	239	54.1
	Cimetidine	267	60.4
	Antibiotic/s	54	12.2
	Hydrocortisone	46	10.4
	Omeprazole	41	9.3
	Metoclopramide	25	5.7
	Dopamine	21	4.8
	Vitamin B complex	16	3.6
	40% glucose	15	3.4
	Calcium gluconate	10	2.3
	Oral rehydration salts (ORS)	8	1.8
	Tramadol	6	1.4
	Antacids	5	1.1
**Admitted to ICU**	Mechanical ventilation and other supportive cares	23	5.2

[♯]Agents with indication frequency of less have been excluded.
*Mechanistically has antidotal activity but not approved yet.

~~**Duration of hospital stay and treatment outcomes of the cases**~~

~~The duration of hospital stay of each case was from the time of admission to termination of hospital stay because of discharge following recovery from poisoning symptoms or because of death. Accordingly, 265 (60.0%) of the poisoned patients were hospitalized for ≤24 hours and the remaining, 94 (21.3%), 48 (10.9%), and 35 (7.9%), were hospitalized for 25-48, 49-72, and longer than 72 hours, respectively (Figure 3). The majority of death records, 15.4%, were among the patients with 24 hours or shorter period of hospital stay. On the other hand, 2.0% and 0.2% of~~

death records were among the patients hospitalized for 25-48 and 49-72 hours, respectively. No fatal case was recorded in the cases hospitalized for longer than 72 hours (Fig 2).

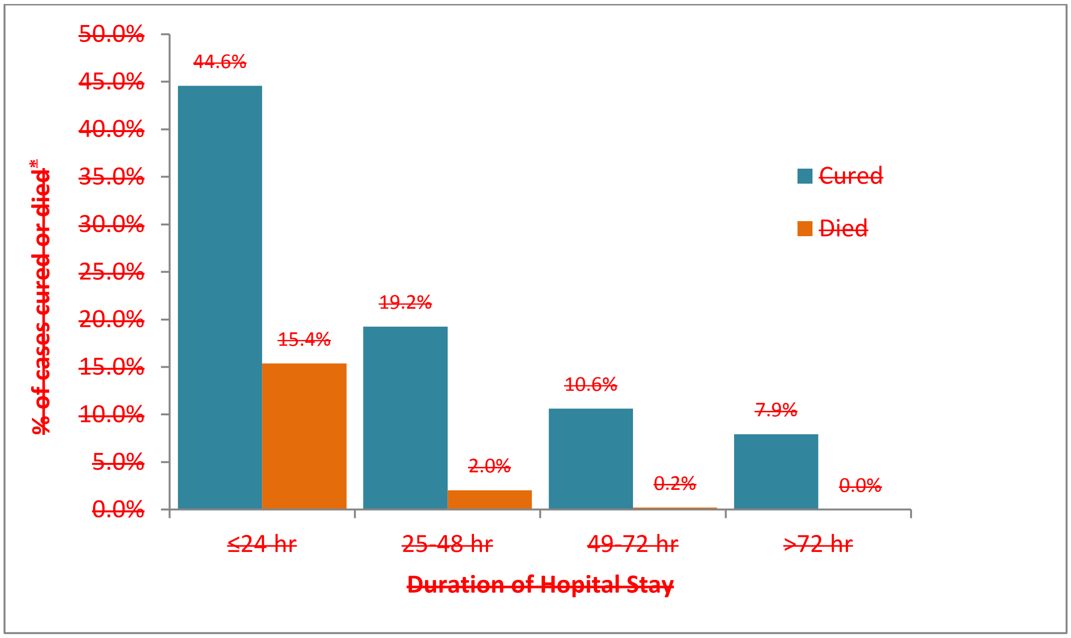

Fig 2. Duration of hospital study and treatment outcome. *Percentages calculated out of the total cases.

The treatment outcome of the poisoning cases by the levels of consciousness

Based on observational and physical assessments upon hospital arrival, the cases were classified as conscious, semiconscious or unconscious by level of consciousness. Accordingly, 208 (47.1%), 107 (24.2%), and 127 (28.7%) of the cases were conscious, semiconscious, and unconscious, respectively, when they arrived at the emergency departments of the hospitals. From the total, 78 (17.6%) of the cases were died. Their distributions by the level of consciousness were 19 (4.3%), 19 (4.3%), and 40 (9.0%) among the cases arrived in conscious, semiconscious, and unconscious state, respectively. This result shows that the majority of the death records were from the cases arrived at the hospitals in unconscious state. Considering the proportion of cases died within each level of consciousness, higher proportion of individuals were died in the cases arrived in unconscious state, 40 (31.5%), followed by 19 (17.8%) in those arrived in semiconscious state (Fig 3).

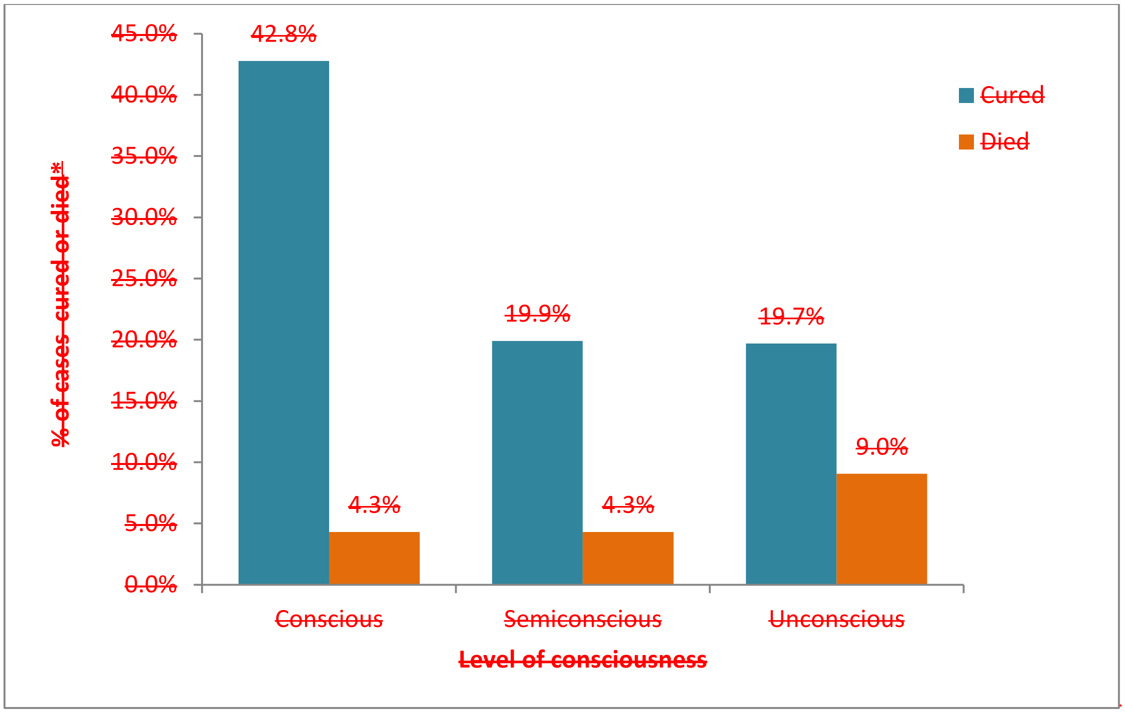

Fig 3. Patient consciousness upon arrival to the hospital and treatment outcome. *percentages calculated out of the total cases.

Factors Associated With Treatment Outcome of Poisoning Emergency

First, bivariate analysis was done for each potential variable assumed to be associated with treatment outcome and the variables that were verified to significantly associate with treatment outcome were included in multivariate logistic regression model. Based on the patients' level of consciousness upon arrival at the emergency departments of the hospitals, those who were in semiconscious state were 12.145 times more likely to die (AOR: 12.145, 95% CI: 3.686-40.019, $p < 0.001$) than those who were in conscious state. A bivariate analysis was done for each potential variable assumed to be associated with the treatment outcome. The variables that were verified to be significantly associated with treatment outcomes in the bivariate were included in the multivariate logistic regression model. Based on the patients' level of consciousness upon arrival at the emergency departments of the hospitals, those who were in a semiconscious state were 12.145 times more likely to die (AOR: 12.145, 95% CI: 3.686-40.019, $p < 0.001$) than those who were in a conscious state. Moreover, those who were in an unconscious state at arrival were 38.423 times more likely to die (AOR: 38.423, 95% CI: 10.408-141.848, $p < 0.001$) than who those were in a conscious state. An hour increase in the time of hospital stay decreased the likely likelihood of death by 9.1% ($\beta = -0.096$, AOR: 0.909, 95% CI: 0.882-0.936, $p < 0.001$).

Patients poisoned with metal phosphides were 6.983 times (AOR: 6.983, 95% CI: 2.559–19.056, $p < 0.001$) more likely to die than those intoxicated with organophosphate agents. In addition, the odds of death among poisoned patients who had no laboratory result as a part of their diagnosis process or investigation of extent of toxicity was 5.376 times (AOR: 5.376, 95% Cl: 1.633–17.704, $p < 0.01$) than ~~of~~ those whose diagnosis involved laboratory tests (Table ~~7~~ 5).

Table ~~7~~ 5. Multivariate analysis of factors associated with treatment outcome (R^2=0.654, Hosmer and Lemeshow goodness of Fit Test, p=0.924).

Explanatory Variables	β-coefficient	Treatment outcome		AOR (95% C.I.)	p-value
		Cured	Died		
**Age (year)**	0.032	-	-	1.033 (0.997-1.069)	0.071
**Residence**					0.215
Rural	-	169	47	1	
Urban	0.693	195	31	0.657 (0.338-1.275)	
**Level of consciousness[1]**					0.000
Conscious	-	189	19	1	
Semiconscious	2.497	88	19	**12.145 (3.686-40.019)*** **	
Unconscious	3.649	87	40	**38.423 (10.408-141.848)*** **	
**Distance (km)[2]**	0.004	-	-	1.004 (.986-1.022)	0.686
**Time of arrival (hr)[3]**	-0.040	-	-	0.961 (0.890-1.038)	0.312
**Duration of hospital stay**	-0.096	-	-	**0.909 (0.882-0.936)*** **	0.000
**Major poison groups**					0.000
Organophosphates	-			1	
Metal phosphides	1.944			**6.983 (2.559-19.056)*** **	
**Laboratory test**					0.006
Yes	-	111	11	1	
No	**1.682**	253	67	**5.376 (1.633-17.704)** **	

The cut point for significant association is at p<0.05.

*p < 0.05, **p < 0.01, ***p < 0.001

[1]Level of consciousness measured just on arrival to the referral hospitals

[2]The distance is from the place of exposure to the referral hospital.

[3]Time of arrival is the time from the time of exposure to arrival to the referral hospitals.

Discussion

This study is dedicated to investigate poisoning in concern with the poisoning patterns in relation to socio-demographic characteristics, the type of poisoning agents involved, treatments given and associated factors with clinical outcomes as death or cure among poisoning cases attended the selected referral hospitals in Amhara Regional State, Ethiopia. The main findings of the study are explained and/or compared with previously published findings of similar studies.

The incidence of poisoning incidence may vary with different categories of socio-demographic characteristics such as age group, sex, residence, and occupation. In this study, the majority of the poisoning cases were females, (63.1%). This result is consistent with the results of previous studies in Ethiopia and Taiwan, which reported 59.20% and 54.76% of incidences, respectively, were females [6, 13]. More than half, 53.2%, of the cases were in the age group of 21–40 years, and this is in line with the results of other similar studies [7, 14]. Regarding residence, the poisoning incidences were found to be somewhat greater in the urban areas, 51.1% in urban and 48.9% in rural areas. This difference might be, at least in part, because of some of the cases from the rural areas may not attend hospitals as they may be treated in health posts, health centers, or district hospitals in their locality. Considering the distribution of the cases by marital status, 50.5% of the poisoned patients were single. This proportion is lower than reports of similar studies in Addis Ababa, Ethiopia and Selected Hospitals in Western Ethiopia, which are 54.98% and 57.10%, respectively [6, 15]. The largest proportion of the poisoning cases were students (31.4%), followed by farmers (29.4%) in occupation. This may be due to the fact that students are prone to emotional liability associated with academic failure and love-related issues leading to intentional poisoning for suicidal purpose.

As it can be depicted from Table 2, the time of arrival to the health facilities seems correlated with the distance from the patients' residential areas to the hospitals. Accordingly, albeit not at all points, the proportion of patients who arrived after 2 hours of the poisoning incidence increased as the distance category from where the patients came was more remote. This might have negative impact on the treatment outcome as the poisoning is a medical emergency that should be treated as soon as possible after its occurrence. Also regarding their consciousness when arrived at the emergency departments of the hospitals, more than half of the cases, 52.9%, were arrived with their level of consciousness compromised. Most of the deaths were among the

~~patients arrived with their level of consciousness compromised, especially in those that were unconscious. This shows that treatment outcome might be affected by the level of consciousness of the victims when arrived at the health facilities. Therefore, it is advisable to come to the health facilities soon after the incidence of poisoning before the symptoms of poisoning is worsened and consciousness is altered.~~

Regarding the poisoning agents involved, organophosphate compounds~~,~~ and metal phosphides (mainly aluminum phosphide)~~,~~ were the first and the second most commonly reported causes of poisons, followed by other agents such as bleaching agents, medicines, unknown chemicals regarded as rat poison, alcohol, and carbon monoxide in decreasing order of frequency. Concerning organophosphates, this result is consistent with the findings of other studies reporting these compounds as the most frequently encountered poisoning agents [14, ~~17-19~~ 16-18], but inconsistent with another study at Jimma University Specialized Hospital [10]. In Ethiopia organophosphate compounds and aluminum phosphide are widely available as pesticides. This may increase the availability and ease their accessibility for intentional poisoning.

~~As shown in Table 5 of the results section, the majority of poising cases were caused by poisons of solid and liquid dosage forms and the commonest route of exposure was the oral route. The liquid dosage forms with other routes of exposure were snake venoms because of five snake bite accidents and one topical exposure of an organophosphate compound. The inhalational route of exposure was for gaseous forms of toxicants and majorly carbon monoxide poisoning.~~ Concerning the sources of the poisoning agents, the majority were obtained at residential homes and local shops. This result is in agreement with the results of other studies in that most of the poisoning agents were obtained at home [7~~.~~,15]. This could be because of the inappropriate storage of the poisoning agents at home, when leftover from their use as insecticides or against grain storage pastes. ~~The second most frequently reported sources of poisoning agents were shops and is in line with the result report by a similar study carried out in western Ethiopia [9].~~ Shops were found to be the second most frequently reported source of poisoning agents, and this result is consistent with the result reported by a similar study carried out in western Ethiopia [9]. The most common reasons for intentional poisoning were dispute-related, mainly family disharmonies, followed by psychiatric conditions, love affairs, and unidentified causes, among

others. The reasons reported in this study are similar ~~with~~ to those in a systematic review by Chelkeba *et al* [~~20~~ 19]. Furthermore, regarding the most frequently reported reason for poisoning, the result of this study is in line with other previous studies [~~17, 21~~ 16, 20].

~~Pharmacologic and nonpharmacologic methods of treatment were employed in managing the poisoning cases.~~ Various interventions were used to treat the poisoning cases. The sole proven antidote among the pharmacologic treatments mentioned was atropine, known to be an antidote ~~of~~ to organophosphate poisoning. Magnesium~~ sulphate~~ sulfate has been reported ~~for having~~ to have beneficial effects ~~in~~ on aluminum phosphide ~~poising~~ and organophosphate poisonings [~~22, 23~~ 21, 22]. ~~Mechanistically, it has antidotal activities, especially against organophosphate poisoning [22 21]. However, it has not been approved as an antidote for either organophosphate or aluminum phosphide poisoning (no literature search results showing its approval).~~ The remaining pharmacologic agents are used more ~~of~~ for supportive treatment of poison-related symptoms rather than for antidotal effects. The most commonly employed nonpharmacologic interventions were supportive care~~s~~ (ABC, mainly IV resuscitation), and this finding is favorable as ensuring a protected airway, adequate ventilation, and hemodynamic stability through the ABC~~-~~approach is crucial in the management of poisoning emergenc~~y~~ies [~~20~~ 19].

Gastric lavage was ~~the second most frequently used nonpharmacologic approach~~ the only GI decontamination method used ~~for poisoning management~~ identified in this assessment. Gastric lavage is indicated for immediate removal of stomach contents after an orally ingested overdose or poisoning [~~24~~ 23]. The use of this method in about 17.0% of the cases~~,~~ in this study~~,~~ was most probably from this perspective. ~~But~~ However, it is not recommended for routine use and should not be considered unless a patient has ingested a potentially life-threatening amount of a poison [~~25~~ 24]. It should be considered only when there is a risk of significant toxicity or imminent fatality, and in situations ~~of~~ where antidotal or other supportive modalities are inadequate, and after contraindications are ruled out [~~24~~ 23]. Because its efficiency in ~~removal of~~ removing the poisoning agents was found to be highly variable and diminished with time in experimental studies and clinical studies, it showed limited beneficial effects. Moreover, the procedure is associated with serious risks, including hypoxia, dysrhythmias, perforation of the GI tract or pharynx, aspiration pneumonitis, and others [~~26~~ 25]. Therefore, consideration should first be given to other less invasive modalities of gastrointestinal decontamination, such as activated

charcoal or whole-bowel irrigation [~~24~~ 23]. However, there was no report of using other GI decontamination agents or methods such as activated charcoal and whole bowel irrigation. ~~Interestingly, among all the cases, dialysis was used only for in one case. This may might be because of unavailability of the facility or unaffordability of the procedure, but might not be because of need for this intervention~~. Interestingly, among all the cases, dialysis was used only in one case. This might be because of unavailability of the facility or unaffordability of the procedure, but not because cases needing the intervention were rare.

~~The duration of hospital stay of each case was considered as of from the time of admission to termination of hospital stay because of discharge following recovery from poisoning symptoms or because of death. Based on this the majority of the cases (60.0%) were hospitalized for not longer than 24 hours. As it can be depicted from Fig 2, most of the death records were from the cases with hospital stays of ≤24 hours, accounting for 15.4% of the deaths out of the total 17.6% fatality rate. This could be because as the cases died no further care is required and immediately discharged from the hospitals and is not indicative of a relation between short duration of stay and high fatality rate. In the other time categories the fatality rates were lower and even no record of fatality was noted among the cases hospitalized for longer than 72 hours. The mean duration of hospital stay was found to be 34.37 hours. This is longer than the findings of previous study by Adinew *et al* [21] and Dessalew *et al* [27] which are 11.26 hours and 1 day, respectively.~~

The case fatality rate from acute poisoning was found to be 17.6%. This finding is much higher than previous findings of similar studies carried out in Ethiopia [6, 9, 13, 15, ~~28~~ 26] and India [14], ranging 4.10-10.20%. Out of 78 ~~cases died~~ deaths, ~~aluminum~~ metal phosphides ~~was~~ were responsible for 55.1% of the deaths, whereas organophosphates were attributed to 32.1% of the deaths. Even though bleaching agents were the third most common cause of poisoning, no death was recorded among the victims poisoned by these agents. Organophosphates, aluminum phosphide, and other chemicals are available in Ethiopia for use as rodenticide, insecticide, or herbicide [~~29~~ 27]. ~~and~~ However, a comparative study showed the Ethiopian officers who handle these chemicals are less knowledgeable about ~~these~~ the products [~~30~~ 28]. Aluminum phosphide is widely available in Ethiopia with approved use for the control of maize weevils and flour beetles on stored maize [~~29~~ 27]. Organophosphate compounds, especially malathion, are also

among the commonly available pesticides for agricultural uses [30 28]. ~~Their availability and ease of accessibility plus their inherent toxicity is likely to pose a great health burden in the community.~~ Because of their wide availability and ease of accessibility, plus their inherent toxicity, these agents are more likely to pose a great health burden to the community. This result may dictate the need for the design and implementation of strategies to create awareness about and prevent ~~the~~ poisonings by such frequently encountered agents in the community.

Multivariate logistic regression analysis was done to identify predictors of treatment outcome in the present study. Accordingly ~~age~~, level of consciousness upon hospital arrival, ~~distance from the place of poisoning incidence to the hospitals~~, length of hospitalization, type of poisoning agents encountered, and use of laboratory test as a part of diagnosis were identified as significant potential predictors of treatment outcome. Based on the level of consciousness, the patients ~~that~~ who were in semiconscious and unconscious states upon hospital arrival were 12.145 and 38.423 times more likely to die, respectively, than those who were conscious. The level of consciousness may be compromised due to a delay ~~of~~ in intervention or because of the inherent toxic characteristics of a poisoning agent, leading to rapid loss of consciousness and ~~affect~~ negatively affecting the treatment outcome ~~negatively~~. ~~The odd of the likely to die was not significantly increased with distance.~~ On the other hand, the analysis showed that as the length of hospitalization increased the probability of death was decreased. This could be the case because the likelihood of death decreased once the patients managed to overcome acute symptoms and received care after being admitted to the hospitals. There was no discernible relationship between distance and treatment outcome. It makes sense that the treatment outcome would be poor consistently the further a patient traveled to get to the hospital. The ~~inconsistent~~ findings of the present study in this regard could be the consequence of pre-hospital interventions carried out at home, or in community health centers, or smaller general hospitals, which were not taken into account in this study. Patients poisoned with metal phosphides were 6.983 times more likely to die ($p < 0.001$) than those intoxicated with organophosphate agents. Moreover, 43 of 78 the fatal cases were among metal phosphide-poisoned patients. ~~This might indicate a higher severity of metal phosphide poisoning and should be considered as a great public health threat in the community.~~ This could indicate that metal phosphide poisoning is more severe and should be considered as a great public health threat in the community. The ~~odds~~ likelihood of death among poisoned patients who had no laboratory result as a part of their diagnosis process was 5.376

times than that of those whose diagnosis involved laboratory tests. This might be because the laboratory diagnosis can help investigate the severity of toxicity and direct ~~to better~~ treatment intervention accordingly.

detailed

The prospective nature of the study design enables us to collect detail clinical and laboratory data to determine the severity of the cases as a determinant of treatment outcome. This can be taken as the strength of this study ~~[27]. But~~ However, ~~this~~ the study lacks some parameters including pre-hospital interventions (that might be given at home or local health facilities), referral cases, and appropriateness of interventions ~~at the hospitals~~. ~~Missing~~ The absence of such variables can be considered ~~as the~~ a limitation of this study. Especially, the result of this study may be affected by referral bias. If these variables were included, it would be better for a more appropriate explanation of potential predictor variables of the treatment outcome. For example, the likelihood of death was not significantly increased with time of delay for hospital arrival, and this might be because of pre-hospital interventions at home or local health centers. ~~and~~ It does not indicate that time of delay to come after intoxication has no negative impact on treatment outcome. Similarly, the inconsistent association between the distances from the areas where the poisoning incidence occurred to the hospitals would be more objectively explained if the possible pre-hospital interventions and referral cases were considered.

Conclusion

The majority of the poisoning cases were females. The most common reasons for the intent of self-poisoning were dispute-related, mainly family disharmonies, followed by psychiatric conditions. The poisoning agents were mostly obtained from households. Organophosphate compounds and metal phosphides were the first and the second most frequently encountered poisoning agents, respectively, and it was noted that the later ones were responsible for most of the fatal cases. Of the pharmacologic, atropine was the only agent regarded as antidote. The most commonly employed agent for supportive treatment was cimetidine followed by maintenance fluids, while gastric lavage was the only GI decontamination method used among others. ~~Cimetidine was the most often used agent for supportive treatment, followed by maintenance fluids and gastric lavage was the only GI decontamination techniques that was utilized.~~ The fatality rate of poisoning in this study was found to be much higher than in other similar studies. Impaired consciousness upon hospital arrival, type of poisoning, and no involvement of

laboratory investigation were found to significantly associate with the likelihood of death. Generally the results dictate the need for the design and implementation of strategies to create awareness, about and prevent, and manage poisonings incidences by such frequently encountered agents in the community. Impaired consciousness upon hospital arrival, type of poisoning, and no involvement of laboratory investigation were found to significantly associate with the likelihood of death.

Availability of data

The data set of the study is available online and can be accessed without any restriction.

Authors' contributions

All authors were participated in the conception, design, and development of the proposal of the study for grand competition and in supervision of the data collection process. GMA had substantial contribution in the preparation of the data collection tool. ABA accomplished the data entry, analysis, interpretation, and the writing up of the manuscript. Finally, all authors read and approved the submission of the manuscript for publication. Moreover, all of the authors are responsible regarding the appropriateness and originally of this work.

Acknowledgements

We are grateful to the administration offices of the hospitals for their cooperation in facilitating the official process of getting permission to undertake the study in their respective institutions. Besides, we are thankful to the physicians and nurses who were working in the emergency departments of the hospitals for their unwavering cooperation in the data collection process.

Competing interests

The authors have declared that no competing interests exist.

Funding

This study was done using a grant awarded by University of Gondar, Ethiopia (Reference №: R/T/T/C/Eng.58/03/2018). The funder had no role in the study design, data collection and analysis, decision to publish, or preparation of the manuscript.

References

1. G/Mariam ET, Gelaw BK. Global Epidemiology of Acute Poisoning with an Emphasis to Ethiopia: Systematic Review. *Int J Pharm Sci & Scient Res*. 2016; 2(4): 120-130.
2. WHO. 2021. Chemical Safety and Health: Prevention and management of cases of poisoning. Accessed on November 12, 2022. Available on https://www.who.int/teams/environment-climate-change-and-health/chemical-safety-and-health/incidents-poisonings/prevention-and-management-of-cases-of-poisoning
3. Mew EJ, Padmanathan P, Konradsen F, Eddleston M, Chang SS, Phillips MR, Gunnell D. The global burden of fatal self-poisoning with pesticides 2006-15: Systematic review. *J Affect Disord*. 2017;219:93-104.
4. Abd-Elhaleem ZAE, Muqhem BAA. Pattern of acute poisoning in Al Majmaah region, Saudi Arabia. *Am J Clin Exp Med*. 2014; 2(4): 79–85.
5. Kebede D, Alem A. Suicide attempts and ideation among adults in Addis Ababa, Ethiopia. *Acta Psychiatr Scand Suppl*. 1999; 397:35-39.
6. Woyessa AH, Palanichamy T. Patterns, Associated Factors, and Clinical Outcomes of Poisoning among Poisoning Cases Presented to Selected Hospitals in Western Ethiopia: Hospital-Based Study. *Emerg Med Int*. 2020; 2020:5741692.
7. Getie A, Belayneh YM. A Retrospective Study of Acute Poisoning Cases and Their Management at Emergency Department of Dessie Referral Hospital, Northeast Ethiopia. *Drug Healthc Patient Saf*. 2020;12:41-48.
8. Teklemariam E, Tesema S, Jemal A. Pattern of acute poisoning in Jimma University Specialized Hospital, South West Ethiopia. World J Emerg Med. 2016;7(4):290-293.
9. Singh S, Sharma BK, Wahi PL, Anand BS, Chugh KS. Spectrum of acute poisoning in adults (10 year experience). *J Assoc Physicians India*. 1984 Jul;32(7):561-3. PMID: 6501193.
10. Padmanabha TS, Gumma K, Kulkarni GP. Study of profile of organophosphorus poisoning cases in a tertiary care hospital, North Karnataka, Bidar, India. *Int J Pharm Bio Sci*. 2014;5(1):332-339.
11. Kumar S, Pathak A, Mangal HM. Trends of fatal poisoning in Saurashtra region of Gujarat (a prospective study). *Journal of Indian Academy of Forensic Medicine*. 2011; 33(3):197-199.
12. Somasundaram KV, Patil A, Shukla SK. Epidemiological profile of OP poisoning cases treated at Pravara hospital, Loni, India. *Indian J Prev Soc Med*. 2009;40(3&4):184-188.

13. Lee HL, Lin HJ, Yeh ST, Chi CH, Guo HR. Presentations of patients of poisoning and predictors of poisoning-related fatality: findings from a hospital-based prospective study. *BMC Public Health*. 2008;8:7.
14. Kumar SV, Venkateswarlu B, Sasikala M, Kumar GV. A study on poisoning cases in a tertiary care hospital. *Journal of natural science, biology, and medicine*. 2010;1(1):35.
15. Zemedie B, Sultan M, Zewdie A. Acute Poisoning Cases Presented to the Addis Ababa Burn, Emergency, and Trauma Hospital Emergency Department, Addis Ababa, Ethiopia: A Cross-Sectional Study. *Emerg Med Int*. 202; 2021:6028123.

~~Alghafees MA, Abdulmonen A, Eid M, Alhussin GI, Alosaimi MQ, Alduhaimi GS, Albogami MT, Alhelail M. Poisoning-related emergency department visits: the experience of a Saudi high-volume toxicology center. *Ann Saudi Med*. 2022;42(1):36-44.~~

16. Adinew GM, Woredekal AT, DeVos EL, Birru EM, Abdulwahib MB. Poisoning cases and their management in emergency centres of government hospitals in northwest Ethiopia. *Afr J Emerg Med*. 2017;7(2):74-78.
17. Jamie AH, Abdosh MZ. Patterns and Clinical Outcomes of Poisoning Among Poisoning Cases Presented in a Hospital: A Retrospective Study. *Lokman Hekim Health Sci*. 2021;1(3):70–73.
18. Ramesha KN, Rao KB, Kumar GS. Pattern and outcome of acute poisoning cases in a tertiary care hospital in Karnataka, India. *Indian J Crit Care Med*. 2009;13(3):152-155.
19. Chelkeba L, Mulatu A, Feyissa D, Bekele F, Tesfaye BT. Patterns and epidemiology of acute poisoning in Ethiopia: systematic review of observational studies. *Arch Public Health*. 2018 Jul 2;76:34.
20. Adinew GM, Asrie AB, Birru EM. Pattern of acute organophosphorus poisoning at University of Gondar Teaching Hospital, Northwest Ethiopia. BMC Res Notes. 2017;10(1):149.
21. Eddleston M, Buckley NA, Eyer P, Dawson AH. Management of acute organophosphorus pesticide poisoning. The Lancet. 2008 ;371(9612):597-607.
22. Karimani A, Mohammadpour AH, Zirak MR, Rezaee R, Megarbane B, Tsatsakis A, Karimi G. Antidotes for aluminum phosphide poisoning–An update. Toxicology reports. 2018 1;5:1053-1059.

23. Jenny J. Lu. Gastric Lavage. Emergency Medicine Procedures., edited by Eric F. Reichman, McGraw-Hill Education, 2013, 393-398
24. Vale JA. Position statement: gastric lavage. American Academy of Clinical Toxicology; European Association of Poisons Centres and Clinical Toxicologists. *J Toxicol Clin Toxicol*. 1997;35(7):711-719.
25. Benson BE, Hoppy K, Troutman WG, Bedry R, Erdman A, Höjer J, *et al*. American Academy of Clinical Toxicology; European Association of Poisons Centres and Clinical Toxicologists. Position paper update: gastric lavage for gastrointestinal decontamination. *Clin Toxicol (Phila)*. 2013;51(3):140-146.

~~Desalew M, Aklilu A, Amanuel A, Addisu M, Ethiopia T. Pattern of acute adult poisoning at Tikur Anbessa specialized teaching hospital, a retrospective study, Ethiopia. *Hum Exp Toxicol*. 2011;30(7):523-527.~~

26. Tefera GM, Teferi LG. Prevalence, Predictors and Treatment Outcome of Acute Poisoning in Western Ethiopia. *Open Access Emerg Med*. 2020;12:365-375.
27. Ethiopian Ministry of Agriculture. List of registered pesticides. Accessed on July 17, 2022. http://www.moa.gov.et/list-of-registered-pesticides
28. Tessema RA, Nagy K, Ádám B. Pesticide Use, Perceived Health Risks and Management in Ethiopia and in Hungary: A Comparative Analysis. *Int J Environ Res Public Health*. 2021;18(19):10431.

Response to Reviewers

Manuscript Title: Poisoning Cases and Their Management in Amhara Region, Ethiopia: Hospital-Based Prospective Study

Manuscript Submission Number: PONE-D-22-26894R2

Journal: PLOS ONE

Dear Editor,

We are very thankful to you for handling the publication process of this manuscript.

Modified portions or newly added texts in the revision process are marked-up with greed color, whereas deletions are shown by red colored texts with Strikethrough line, ~~example~~.

Response to Reviewer #3

Dear Reviewer,

We are grateful for your dedication and time spent in reviewing this manuscript. The comments forwarded are greatly appreciated and used to help improve the manuscript. They have been addressed as follows.

Reviewers' comments:

Reviewer's Responses to Questions

Comments to the Author

1. If the authors have adequately addressed your comments raised in a previous round of review and you feel that this manuscript is now acceptable for publication, you may indicate that here to bypass the “Comments to the Author” section, enter your conflict of interest statement in the “Confidential to Editor” section, and submit your "Accept" recommendation.

   Reviewer #3: (No Response)

2. Is the manuscript technically sound, and do the data support the conclusions?

   Reviewer #3: Partly

   Thank you!

By taking into account the feedback you sent we have made changes to improve the manuscript in this regard. We include additional points in the conclusion part.

3. Has the statistical analysis been performed appropriately and rigorously?

   Reviewer #3: I Don't Know

   As the result is dichotomous, i.e., survived or died, we have performed binary logistic regression adjusted to potential factors that might affect patient outcome (as cured or died). First we performed bivariate logistic regression to identify potential predictors of the patient outcome. Then the factors found to significantly associate with probability of death in bivariate analysis were included in multivariate analysis. We performed backward stepwise elimination logistic regression and we selected the best model among the alternative models found in the analysis.

4. Have the authors made all data underlying the findings in their manuscript fully available?

   Reviewer #3: No

   We think that we made sufficient data underlying the findings available in the manuscript. Moreover, the full data set was submitted to the journal during manuscript submission. However, as another reviewer commented that there are too many tables and figures and the result could be summarized in 2-3 tables, 2 tables and 2 figures have been removed in this version.

5. Is the manuscript presented in an intelligible fashion and written in standard English?

   Reviewer #3: No

   Thanks! We have reviewed the entire document and made a number of changes to fix grammatical errors. We corrected some grammatical errors using QuillBot online grammar checker. Additionally, the document was revised for language clarity by Dr. Yoseph, a senior English language professional (PhD in Teaching English as a Foreign Language (TEFL)) at University of Gondar, and further corrections were made based on his recommendation.

6. Review Comments to the Author

Reviewer #3: You need be more specific about design, although it is not explicitly stated it appears that you targeted a total of 440 patients recruiting 110 in each site. If that is the case

you should say that explicitly including whether all consecutive patients were approached, how many refused (and include some basic information about the refusal such as the poison they were exposed to , gender and age) Further how Lon did it take you in each site to recruit the patients eg how many weeks

Thank you for your concern!

The number of cases targeted was determined to be 442 (based on sample size calculation, page 4 of the previous version of the manuscript). And then, the subjects were divided to the four hospitals considered in this study.

- University of Gondar Comprehensive Specialized Hospital = 110 patients
- Felege Hiwot Referral Hospital = 110 patients
- Debre Markos Referral Hospital = 111 patients
- Dessie Referral Hospital = 111 patients

The number of cases (110 or 111) to be targeted from each site was determined simply by lottery method.

From preliminary assessment we made before the data collection, we had been informed that irregular numbers of poisoning cases attend each hospital. Referring log books in the emergency departments and communicating the healthcare professionals working in the emergency department of each hospital, we had been informed that the number of poisoning cases attending the departments in each year is unpredictable and roughly ranges 80 to 130 patients per year. Because of this we couldn't approach randomly selected cases and rather we decided to and approached all consecutive cases until we got the number of cases taken from each hospital. Very few cases refused to participate in the study (refusal was so rare) and had been excluded from the study. In addition, very few cases from whom adequate data were not obtained had been excluded from the study. However, we collected no data from such cases so that we have no information about them at hand.

The data collection was started on the same date at all sites, whereas there were some differences in the length of time needed to finish the data collection at each site. In all

hospitals, the data collection was started on January 01, 2018 and completed in December 2018 on different specific dates shown below.

- University of Gondar Comprehensive Specialized Hospital = on December 26, 2018
- Felege Hiwot Referral Hospital = on December 24, 2018
- Debre Markos Referral Hospital = on December 21, 2018
- Dessie Referral Hospital = on December 29, 2018

It was taken almost a year to complete the data collection at each site. For this reason, we stated that the data collection period ran from January to December of 2018 (to collect the data from the total of 442 cases).

Response to Reviewer #4

Dear Reviewer,

Thank you for your time and effort invested in reviewing this manuscript. The comments raised are highly valued and used as input to improve the manuscript. They have been addressed as follows:

Reviewers' comments:

Reviewer's Responses to Questions

Comments to the Author

1. If the authors have adequately addressed your comments raised in a previous round of review and you feel that this manuscript is now acceptable for publication, you may indicate that here to bypass the “Comments to the Author” section, enter your conflict of interest statement in the “Confidential to Editor” section, and submit your "Accept" recommendation.

   Reviewer #4: (No Response)

2. Is the manuscript technically sound, and do the data support the conclusions?

   Reviewer #4: No

   Thank you!

We think that this issue has been improved through addressing the feedbacks you provided under the "Review comments to Author".

3. Has the statistical analysis been performed appropriately and rigorously?
   Reviewer #4: No
   As the outcome measure is dichotomous, i.e., survived or died, we have performed binary logistic regression adjusted to potential factors that might affect the outcome (as cured or died). Initially, we performed bivariate logistic regression to identify potential predictors of the patient outcome. Then the factors that were found to be significantly associated with the probability of death in bivariate analysis were included in multivariate analysis. We performed backward stepwise elimination logistic regression and we selected the best model among the alternative models found in the analysis.
4. Have the authors made all data underlying the findings in their manuscript fully available?
   Reviewer #4: Yes
5. Is the manuscript presented in an intelligible fashion and written in standard English?
   Reviewer #4: No
   We reviewed the manuscript to increase the clarity and ease of understandability. Accordingly, we made many changes at various sections in the process of revision.
6. Review Comments to the Author

Reviewer #4: Manuscript ID: PONE-D-22-26894R2

Thank you for the opportunity to review this manuscript entitled: “Poisoning Cases and Their Management in Amhara Region, Ethiopia: Hospital Based Prospective Study”

There are still numerous errors relating to grammar, syntax and academic one. I suggest that the authors employ the services of a professional English language editor with a background in medical journal articles

We have gone through the whole document and made numerous amendments in this regard. First we made corrections on grammatical errors using “quillbot online grammar checker”. Additionally, the document was revised for language clarity by Dr. Yoseph, a senior English language professional (PhD in Teaching English as a Foreign Language (TEFL)) at University of Gondar, and further corrections were made based on his recommendation. The manuscript has been also revised for academic issues by Dr. Zewdneh Shewamene Sabe (working at Population

Council, Ethiopia) and additional corrections were made (eg, deletion of portions in the result and discussion parts). But we are unable to get professional language editor with a background in medical journal articles.

In the Methods section of the Abstract it is stated that data collection was from Jan to Dec 2028, which implies that a convenience sampling was used. However, in the main text under Methods, the authors indicate that sample size determination was used. This is conflicting and needs to be clarified.

Thank you!

First, we determined the number of cases to be included in the study by calculating the sample size as stated in the manuscript (not conveniently determined). Then, we distributed the 442 samples to the four study sites; 111 cases to each of two hospitals and 110 cases to each of the other two hospitals. After this, we started data collection and the period stated is the time required to collect the data in each hospital.

At all sites, the data collection was started on January 1, 2018 and completed in the fourth week of December 2018 on different specific dates. For this reason, we stated that the data collection period ran from January to December of 2018. The time was not predetermined before the data collection. We determined the sample cases to be considered first, distributed them to the four study sites. It took the stated period (January to December 2018) to complete the data collection at each site.

As of the information we got from the emergency departments of the hospitals before starting the data collection, the cases flow was not predictable. Because of this we couldn't follow a specific sampling technique and rather we decided to consider all consecutive cases till we got the specified number of patients in each hospital. There were few refusal cases and few others with incomplete data and excluded from the study. However, since the data was collected until we achieved the predetermined number of subjects in each study site; the total cases targeted are equal to the originally calculated sample size.

Overall, there are too many tables and figures. Most of the data can be included in 2-3 tables. Also, there is a lot of repetition between the text and figures/tables.

Thank you!

In consideration of this comment, 2 tables and 2 figures that we feel are redundant or do not contain as much relevant information have been removed. The discussion points pertaining to the contents of these tables and figures have also been eliminated. These are the figures and tables that have been removed.

- **Table 2** on Page 7…Variable included in the multivariate analysis
- **Table 4** on page 8…We think the content is not as much relevant.
- **Figure 2** on page 11…The variable included in the multivariate analysis
- **Figure 3** on page 12….Because the content appeared in the multivariate analysis result table.

Table 3: Seems like "Died" and "Cured" under "Treatment outcome" have been incorrectly labelled. The authors are encouraged to read through other similar publications which will assist them with improving the layout and design of this article. The discussion section is too long.

Thank you!

We replaced the term "died" by "cured" and the vice versa.

The discussion part has been reduced from 5 and half pages to about 4 pages.

Please note that:

~~Texts with red font color and strikethrough line~~ like this– indicate deletion in the process of revision.

Texts with green highlight – indicate newly added texts in the revision process.
