# Supplementary material for: Poisoning cases and their management in Amhara National Regional State, Ethiopia: Hospital-based prospective study
Source: PLoS One. 2024 May 31;19(5):e0303438. doi: 10.1371/journal.pone.0303438 (PMC11142576; doi:10.1371/journal.pone.0303438)
Supplement: Attachment — Submitted filename: Response to Reviewer.docx [file pone.0303438.s003.docx]

**Response to Reviewer**

PONE-D-22-26894R1
Poisoning Cases and Their Management in Amhara Region, Ethiopia: Hospital Based Prospective Study PLOS ONE

Dear Dr. Asrie,

Thank you for submitting your manuscript to PLOS ONE. After careful consideration, we feel that it has merit but does not fully meet PLOS ONE’s publication criteria as it currently stands. Therefore, we invite you to submit a revised version of the manuscript that addresses the points raised during the review process.

Unfortunately the two reviewers from the first round were not available to reassess your manuscript, so we have sought input from an additional reviewer whose report can be found below. As you will see from the comments, there remain significant concerns relating to the framing of the study within the body of existing literature and the reporting of the methodology which must be addressed before your manuscript is suitable for publication.

Please submit your revised manuscript by Apr 15 2023 11:59PM. If you will need more time than this to complete your revisions, please reply to this message or contact the journal office at [plosone@plos.org](mailto:plosone@plos.org). When you're ready to submit your revision, log on to https://www.editorialmanager.com/pone/ and select the 'Submissions Needing Revision' folder to locate your manuscript file.

Please include the following items when submitting your revised manuscript:

- A rebuttal letter that responds to each point raised by the academic editor and reviewer(s). You should upload this letter as a separate file labeled 'Response to Reviewers'.
- A marked-up copy of your manuscript that highlights changes made to the original version. You should upload this as a separate file labeled 'Revised Manuscript with Track Changes'.
- An unmarked version of your revised paper without tracked changes. You should upload this as a separate file labeled 'Manuscript'.

All the above items have been included in this submission.

If you would like to make changes to your financial disclosure, please include your updated statement in your cover letter. Guidelines for resubmitting your figure files are available below the reviewer comments at the end of this letter.
If applicable, we recommend that you deposit your laboratory protocols in protocols.io to enhance the reproducibility of your results. Protocols.io assigns your protocol its own identifier (DOI) so that it can be cited independently in the future. For instructions see: https://journals.plos.org/plosone/s/submission-guidelines#loc-laboratory-protocols. Additionally, PLOS ONE offers an option for publishing peer-reviewed Lab Protocol articles, which describe protocols hosted on protocols.io. Read more information on sharing protocols athttps://plos.org/protocols?utm_medium=editorialemail&utm_source=authorletters&utm_campaign=protocols.

We look forward to receiving your revised manuscript.

Kind regards,

Dr Joseph Donlan
Senior Editor
PLOS ONE
[Note: HTML markup is below. Please do not edit.]

Thank you dear editor for handling the peer review process of this manuscript.

Reviewers' comments:

Reviewer's Responses to Questions

**Comments to the Author**

1. If the authors have adequately addressed your comments raised in a previous round of review and you feel that this manuscript is now acceptable for publication, you may indicate that here to bypass the “Comments to the Author” section, enter your conflict of interest statement in the “Confidential to Editor” section, and submit your "Accept" recommendation.

Reviewer #3: (No Response)

2. Is the manuscript technically sound, and do the data support the conclusions?
The manuscript must describe a technically sound piece of scientific research with data that supports the conclusions. Experiments must have been conducted rigorously, with appropriate controls, replication, and sample sizes. The conclusions must be drawn appropriately based on the data presented.

Reviewer #3: Partly

3. Has the statistical analysis been performed appropriately and rigorously?

Reviewer #3: No

We think that the statistical analysis has been improved during modifications based on a specifc comment in this regard (in revier comments to the author below).

4. Have the authors made all data underlying the findings in their manuscript fully available?

The PLOS Data policy requires authors to make all data underlying the findings described in their manuscript fully available without restriction, with rare exception (please refer to the Data Availability Statement in the manuscript PDF file). The data should be provided as part of the manuscript or its supporting information, or deposited to a public repository. For example, in addition to summary statistics, the data points behind means, medians and variance measures should be available. If there are restrictions on publicly sharing data—e.g. participant privacy or use of data from a third party—those must be specified.

Reviewer #3: Yes

5. Is the manuscript presented in an intelligible fashion and written in standard English?
PLOS ONE does not copyedit accepted manuscripts, so the language in submitted articles must be clear, correct, and unambiguous. Any typographical or grammatical errors should be corrected at revision, so please note any specific errors here.

Reviewer #3: No

We have made several modifications for language correcs through out the whole paper.

**Response to Reviwer #3**

Dear reviewer,

Thank you very much for your interesting and valuable comments, which are important to improve the quality of the manuscript to the highest standard possible. The comments have been addressed as follows.

Reviewer #3: The article still has residual errors in grammar, I would suggest the authors use Grammarly or tools within their word processor.

We have made modifications in different parts of the paper for grammatical and/or typographical errors using “quillbot grammar checker” online software.

Poisoning in emerging countries is a significant problem. It seems to me that his article needs to be clear upon its focus. It is long and information is repeated especially in the results section.

Thank you!

Taking this comment in account, we have reframed the contents of the manuscript in such a way that the focus to be on:

- type of poisoning agents encountered
- reasons for poisoning
- treatments given
- patient outcomes
- potential factors associated with poor outcome (death)

Considering the comment, we have removed some contents from the result section that we feel repeated or are not as such relevant.

It is important to describe the nature poisons being seen which this article does well. What is lacking is clearer description of the clinical setting such as rates of inter hospital transfer, antidote supply, health staff training and resources for advanced supportive care. This provides a context for the treatment and the results being provided. Given the breadth of significant toxicity I found the discussion of treatment rationale to be a bit superficial and in places outside established guidelines for treatment in these settings. Your article may describe the treatment your patients received but it is not an article about how to treat poisoned patients

Right! We fully admitted what has been raised about what is lacking in this work.

Regarding hospital transfer, the hospitals accept both referral and direct admission cases. However, this study did not segregate patients as direct admissions and transferred (referral) cases. We have already mentioned this in the discussion section as a limitation of the study.

…antidote supply, health staff training and resources for advanced supportive care

This study is the first phase to be followed by poisoning treatment guideline preparation, establishing poisoning information centers, and preparing training manuals and delivering trainings to the health workers in the hospitals selected and others also. We planned to address the above mentioned issues just before these activities provided that we could secure a fund. Otherwise, even though we can’t support with data at this time and include in the paper, we know from our context that:

- there is no strong antidote supply system
- on job trainings on poisoning and poisoning management are rare and there are few/no health professionals specialized with poisoning management or emergency medicine
- no separate emergency departments for poisoning cases
- there is scarcity of advanced supportive care facility in general and no specific advanced supportive care facilities dedicated for poisoning cases

So we may address these issues in a type of operational research or recommend to be addressed by other bodies including other scholars, the health facilities themselves or health bureau of the region.

Specific comments

Abstract

Poisoning is a significant public health problem globally and cases are being increased from time to time. As the advances in technological and social developments have led to ease accessibility of most drugs and chemical substances in the society, the number of incidences of poisoning can be higher. Ethiopia is one of the low-income countries that share its considerable burden of poisoning incidences and deaths.

Suggest
Poisoning is a significant public health problem globally. Ethiopia is a low-income country undergoing technological and social change that may increase access to drugs and chemicals potentially increasing the incidence of poisoning. This study describes the epidemiology of hospital admissions due to poisoning in a region of Ethiopia.

Thank you! This part has been modified as per this suggestion.

Methods: Include a time period eg 2 years

The time period of the study was one year, and included there in the abstract.

Results

Some reorganisation of the order of the text would be useful and shortening as much of this is repeating the main results section

Eg

There were ZZZ cases with XX deaths. Almost all poisoning cases were intentional self- poisonings The most frequent poisonings were Organophosphate compounds, 145 (32.8%), and metal phosphides (majorly aluminum phosphide), 115 (26.0%). The ingested poison was most frequently accessed from the patients home, 243 (55.0%), followed by purchase from local shops, 159 (36%)

The median duration of admission was XX. YY% required ventilation, the most poisoned case requiring ventilation was (insert compound) 43/78 deaths were due to aluminium phosphide

From the multivariate logistic regression analysis, age, altered level of consciousness on hospital arrival, distance from the area where the poisoning incidence happened to the hospital they attended, and no laboratory result as a part of the diagnosis process or investigation of the extent of toxicity were found to be significantly associated with the likelihood of poor treatment outcome or death.

Thank very much! We have adapted this text with some changes and incorporated in the manuscript (methods in the abstract).

Main Article
Background
I think this could be shortened, the first two paragraphs don’t add much to the background

The paragraph

“Poisoning is a common cause for emergency visit and hospital admission worldwide and a major reason for illness and death in many countries (3). The incidence of poisoning could be intentional or unintentional, and in children, because of the desire for imitating adults, the unintentional or accidental poisoning is frequent among them (4).”

Comment: most hospital admissions for poisoning are intentional and in the context of deliberate self harm. The second sentence regarding children is not supported by the reference nor is it correct that the motivation is a “desire for imitating adults”. In very young children accidental poisoning is common but in ages greater than 12 years it is commonly deliberate self harm with a wide range of precipitating factors including domestic violence, sexual assault, relationship difficulties etc I think this sentace is probably not required

Consider

“Intentional self-poisoning is a common cause for emergency visit and hospital admission worldwide and a major reason for illness and death in many countries (3).” And join this with the subsequent paragraph starting poisoning from pesticides

Thank you! Based on the comments, the first paragraph has been deleted and the sentence about self-poisoning has been merged with the second paragraph (page 3).

Methods
Please clarify are these 4 referral hospitals the only referral hospitals in the region? What population size do they serve?
Are there specific transfer criteria from smaller hospitals for example are all poisoning cases transferred or only severe ones…..if it is the later then that referral bias needs to be discussed later

There were 5 referral hospitals in the region during the study period and 1 specialized hospital was inaugurated lately. Accordingly, there are currently 6 referral hospitals in the region. Four of the referral hospitals were conveniently selected as the study site.

The locations of the hospitals and the catchment area populations they serve have been included in the Methods section (The study area, page 5).

With the current practice, the major criteria (reason) to transfer cases from smaller/primary hospitals to referral hospitals is the severity of the poisoning and/or unsatisfactory response to the treatments given. The referral hospitals also accept and treat cases that are not referred from the smaller hospitals. Accordingly, the poisoning cases in the referral hospitals include both referral cases and direct admissions. However, this study didn’t segregate the cases in this way and discussed as a limitation in the original submission.

Results
Most of the result data sits in the tables, you should not repeat those results in the text rather you can provide a shorter overview of the table. In the discussion section you have an opportunity to bring the various themes together

Thank you for the suggestion.

Socio-demographic

This is well described in the table, your text is really just repeating the information in the table. So try rephrasing the text into a succinct overview of the table. Eg the majority of the patients were under 40 years of age, female, Christian of Amhara ethnicity see table 1

Thank you! We have made changes in this part based on the comment.

Distance and time of arrival to hospital

Please clarify is the primary hospital that they have presented to (ie not the referal hospital)

What percentage were direct admissions to the referral hospital versus transfer…this may have impact on outcomes as well as data interpretation

(see: Senarathna L, Buckley NA, Jayamanna SF, Kelly PJ, Dibley MJ, Dawson AH. Validity of referral hospitals for the toxicovigilance of acute poisoning in Sri Lanka. Bulletin of the World Health Organization. 2012;90:436-43a.)

Thank you for the concern and the reference provided!

The distances and times of arrival at the hospitals are regarding the referral hospitals considered in the study (not the primary hospitals). As we tried to indicate above, this study didn’t identify patients as direct admission or referral cases.

Signs and symptoms

I am not sure what this section adds. The signs and symptoms will often depend upon the poisoning….the frequency of signs and symptoms with various poisons is well described in the literature. It may be more useful to characterise the patients using a poison severity score , or perhaps need for ventilation.

Persson HE, Sjöberg GK, Haines JA, de Garbino JP. Poisoning severity score. Grading of acute poisoning. Journal of Toxicology: Clinical Toxicology. 1998 Jan 1;36(3):205-13.

Davies JO, Eddleston M, Buckley NA. Predicting outcome in acute organophosphorus poisoning with a poison severity score or the Glasgow coma scale. QJM: An International Journal of Medicine. 2008 May 1;101(5):371-9.

Right! We removed the "Signs and Symptoms" section (Table 3 of the previous version) considering this comment. Instead, we kept the level of consciousness data. In each case it was determined using clinical judgment and especially in the patients with altered levels of consciousness using the Glasgow coma scale. Accordingly, the cases were classified as conscious, semiconscious, and unconscious. This result has been presented and discussed in relation to treatment outcomes.

Types of poisoning and death distributions by poisoning agents

The text here is also just duplicating the table. I would suggest a summary statement.
In the table you could potentially add the deaths attributed to each poisoning ie the data that is in table 8

Thank you! We moved the contents of “Table 8” to “Table 4” and the text of this section has been summarized. In the meantime, the title has been modified to **"Types of poisoning agents and death distributions"**. Table 8 has been deleted as it would be a mere repetition.

Dosage form of the poisons encountered and the routes of exposure
Reasons for poisoning incidents
Again the text is just duplicating the tables in these sections

The texts of these section have been also modified as recommended.

Treatment

Probably the most important area of treatment is the need for advanced supportive care such as ventilation. Such treatment has important resource implications. Move the section from the discussion to the results

Thank you! We have moved it to the results part.

You mention gastric lavage but there is no mention of activated charcoal as part of the decontamination.

Even though activated charcoal is a universal adsorbent of various toxins and is used for GI decontamination, it has been years since it disappeared from public pharmacies in Ethiopia. No regiment containing activated charcoal was identified in this study. That is why it is not mentioned as GI decontaminat in the study.

The subsequent list of treatment would be more informative if it focused upon recognised antidotal treatment eg atropine and perhaps calcium and magnesium. The rest of the drugs mentioned to not seem to specifically relate to poisoning

Thank you!

Atropine is the only well-established antidote among the treatments mentioned (Table 7 of the previous version). Magnesium sulfate has been reported to have beneficial effects in almunium phosphide poisoning and organophosphate poisoning(DOI:10.1016/S0140-6736(07)61202-1, DOI: 10.1016/j.toxrep.2018.10.009). However, it has not been approved as an antidote for either organophosphate or aluminum phosphide poisoning (no literature search results show its approval). This is also the case in our country. Rather, it may be considered a supportive treatment. But since mechanistically it has antidotal activities, especially against organophosphate poisoning, we considered it an antidote, even though we didn't get a source showing its approval.

Calcium gluconate is reported to be used to control aluminum phosphide poisoning-associated hyperkalemia (https://doi.org/10.53986/ibjm.2022.0018). But not considered as specific antidode yet. Therefore we feel that it is not appropriate to consider this agents as an antidote.

All other treatments are more of supportive and important as symptomatic treatments in poisoning emergencies.

We have reorganized the contents of table 7 of the original submission, in attempt of making it more informative.

**Duration of hospital stay**

Time is a continuous variable, it is not clear to me why you have moved into a categorical variable, if you have the original data it may worth while in analysing it as continuous data especially for death.

Thank you!

It is possible and appropriate to consider time as a continuous variable in the multivariate analysis of predictors of death, and we included it in the analysis based on this comment (Table 8 of this submission). Additionally, other continuous variables that were already in the analysis categorized form have been analyzed as continuous data, and the associations have been interpreted accordingly.

On the other hand, as in the case of Fig. 2, it seems impossible to consider time as a continuous variable. There are many different records of the duration of hospital stays, each with some number of patients. So it is unmanageable to consider time as a continuous variable to present data as in Fig. 2.

In the predictor of death analysis I think you should include the major poison groups, clinically it is likely that coma is driven by with organophophate or aluminium phosphide toxicity. Type of poisoning may be significant for the distance from primary hospital which would be a surrogate for delay to treatment. An adjusted analysis may help explain this

Thank you!

Based on this recommendation, we have included the major poison groups, organophosphates and metal phosphides (mostly aluminum phosphide), in the predictors of death analysis (Table 8 of this version). Then, the discussion of the predictors of death analysis has been modified in accordance with the result of the analysis.

Discussion

In the discussion you need to discuss the potential referal bias as a potential limitation.

Thank you!

We already tried to discuss the referral bias as a limitation of the study, and we have consolidated it a bit in this revision.

In treatment it would be useful to have some discussion about supply of medications, do you have shortages, how does your antidote stock compare with say WHO essential antidote list. What training do your medical staff have specifically for treatment of poisoning. I think it would be more useful to focus upon these bigger general questions than the discussion of individual drugs. Especially as many of the drugs you are suggesting to have a role in poisoning treatment are not mainstream and don’t feature in evidence based guidelines.

Thank you!

These issues were not included in the focus of this study, so we didn’t collect data regarding them. We have a plan to address these issues in the future, as we indicated above in the introductory part of the comments.

Within the discussion you mention activated charcoal but this doesn’t appear to have been used in your patients

Activated charcoal was mentioned just as an example of agents used for GI decontamination, not as an agent used in our patients. Moreover, the reason why activated charcoal was not mentioned in the agents used in managing our cases is outlined in response to the concern that activated charcoal was not mentioned as part of the decontamination (above).

The need for intubation and ventilation should move into the results

Have been moved to the results part, in the text just above Table 6 of this version.

Referencing
Many of your references are old and have been superceded by bigger studies or systematic reviews….this is particularly the case with treatment

Some referces have been removed in the revision processes, while 2 other references were added (ref. 22 & 23).

**Note that:**

~~Texts with red font color and strikethrough line~~ like this– indicate deletions in the process of revision.

Texts with this color highlight – are modified substitutes for deleted texts or newly added texts.
