# Supplementary material for: Poisoning cases and their management in Amhara National Regional State, Ethiopia: Hospital-based prospective study
Source: PLoS One. 2024 May 31;19(5):e0303438. doi: 10.1371/journal.pone.0303438 (PMC11142576; doi:10.1371/journal.pone.0303438)
Supplement: Attachment — Submitted filename: Response to Reviewers.docx [file pone.0303438.s004.docx]

**Response to Reviewers**

**Manuscript Title:** Poisoning Cases and Their Management in Amhara Region, Ethiopia: Hospital-Based Prospective Study

**Manuscript Submission Number:** PONE-D-22-26894R2

**Journal:** PLOS ONE

Dear Editor,

We are very thankful to you for handling the publication process of this manuscript.

Modified portions or newly added texts in the revision process are marked-up with greed color, whereas deletions are shown by red colored texts with Strikethrough line, ~~example~~.

**Response to Reviewer #3**

Dear Reviewer,

We are grateful for your dedication and time spent in reviewing this manuscript. The comments forwarded are greatly appreciated and used to help improve the manuscript. They have been addressed as follows.

Reviewers' comments:
Reviewer's Responses to Questions

**Comments to the Author**

1. If the authors have adequately addressed your comments raised in a previous round of review and you feel that this manuscript is now acceptable for publication, you may indicate that here to bypass the “Comments to the Author” section, enter your conflict of interest statement in the “Confidential to Editor” section, and submit your "Accept" recommendation.

Reviewer #3: (No Response)

2. Is the manuscript technically sound, and do the data support the conclusions?

Reviewer #3: Partly

Thank you!

By taking into account the feedback you sent we have made changes to improve the manuscript in this regard. We include additional points in the conclusion part.

3. Has the statistical analysis been performed appropriately and rigorously?

Reviewer #3: I Don't Know

As the result is dichotomous, i.e., survived or died, we have performed binary logistic regression adjusted to potential factors that might affect patient outcome (as cured or died). First we performed bivariate logistic regression to identify potential predictors of the patient outcome. Then the factors found to significantly associate with probability of death in bivariate analysis were included in multivariate analysis. We performed backward stepwise elimination logistic regression and we selected the best model among the alternative models found in the analysis.

4. Have the authors made all data underlying the findings in their manuscript fully available?

Reviewer #3: No

We think that we made sufficient data underlying the findings available in the manuscript. Moreover, the full data set was submitted to the journal during manuscript submission. However, as another reviewer commented that there are too many tables and figures and the result could be summarized in 2-3 tables, 2 tables and 2 figures have been removed in this version.

5. Is the manuscript presented in an intelligible fashion and written in standard English?

Reviewer #3: No

Thanks! We have reviewed the entire document and made a number of changes to fix grammatical errors. We corrected some grammatical errors using QuillBot online grammar checker. Additionally, the document was revised for language clarity by Dr. Yoseph, a senior English language professional (PhD in Teaching English as a Foreign Language (TEFL)) at University of Gondar, and further corrections were made based on his recommendation.

6. Review Comments to the Author

Reviewer #3: You need be more specific about design, although it is not explicitly stated it appears that you targeted a total of 440 patients recruiting 110 in each site. If that is the case you should say that explicitly including whether all consecutive patients were approached, how many refused (and include some basic information about the refusal such as the poison they were exposed to , gender and age) Further how Lon did it take you in each site to recruit the patients eg how many weeks

Thank you for your concern!

The number of cases targeted was determined to be 442 (based on sample size calculation, page 4 of the previous version of the manuscript). And then, the subjects were divided to the four hospitals considered in this study.

- University of Gondar Comprehensive Specialized Hospital = 110 patients
- Felege Hiwot Referral Hospital = 110 patients
- Debre Markos Referral Hospital = 111 patients
- Dessie Referral Hospital = 111 patients

The number of cases (110 or 111) to be targeted from each site was determined simply by lottery method.

From preliminary assessment we made before the data collection, we had been informed that irregular numbers of poisoning cases attend each hospital. Referring log books in the emergency departments and communicating the healthcare professionals working in the emergency department of each hospital, we had been informed that the number of poisoning cases attending the departments in each year is unpredictable and roughly ranges 80 to 130 patients per year. Because of this we couldn’t approach randomly selected cases and rather we decided to and approached all consecutive cases until we got the number of cases taken from each hospital. Very few cases refused to participate in the study (refusal was so rare) and had been excluded from the study. In addition, very few cases from whom adequate data were not obtained had been excluded from the study. However, we collected no data from such cases so that we have no information about them at hand.

The data collection was started on the same date at all sites, whereas there were some differences in the length of time needed to finish the data collection at each site. In all hospitals, the data collection was started on January 01, 2018 and completed in December 2018 on different specific dates shown below.

- University of Gondar Comprehensive Specialized Hospital = on December 26, 2018
- Felege Hiwot Referral Hospital = on December 24, 2018
- Debre Markos Referral Hospital = on December 21, 2018
- Dessie Referral Hospital = on December 29, 2018

It was taken almost a year to complete the data collection at each site. For this reason, we stated that the data collection period ran from January to December of 2018 (to collect the data from the total of 442 cases).

**Response to Reviewer #4**

Dear Reviewer,

Thank you for your time and effort invested in reviewing this manuscript. The comments raised are highly valued and used as input to improve the manuscript. They have been addressed as follows:

Reviewers' comments:
Reviewer's Responses to Questions

**Comments to the Author**

1. If the authors have adequately addressed your comments raised in a previous round of review and you feel that this manuscript is now acceptable for publication, you may indicate that here to bypass the “Comments to the Author” section, enter your conflict of interest statement in the “Confidential to Editor” section, and submit your "Accept" recommendation.

Reviewer #4: (No Response)

2. Is the manuscript technically sound, and do the data support the conclusions?

Reviewer #4: No

Thank you!

We think that this issue has been improved through addressing the feedbacks you provided under the "Review comments to Author".

3. Has the statistical analysis been performed appropriately and rigorously?

Reviewer #4: No

As the outcome measure is dichotomous, i.e., survived or died, we have performed binary logistic regression adjusted to potential factors that might affect the outcome (as cured or died). Initially, we performed bivariate logistic regression to identify potential predictors of the patient outcome. Then the factors that were found to be significantly associated with the probability of death in bivariate analysis were included in multivariate analysis. We performed backward stepwise elimination logistic regression and we selected the best model among the alternative models found in the analysis.

4. Have the authors made all data underlying the findings in their manuscript fully available?

Reviewer #4: Yes

5. Is the manuscript presented in an intelligible fashion and written in standard English?

Reviewer #4: No

We reviewed the manuscript to increase the clarity and ease of understandability. Accordingly, we made many changes at various sections in the process of revision.

6. Review Comments to the Author

Reviewer #4: Manuscript ID: PONE-D-22-26894R2

Thank you for the opportunity to review this manuscript entitled: “Poisoning Cases and Their Management in Amhara Region, Ethiopia: Hospital Based Prospective Study”

There are still numerous errors relating to grammar, syntax and academic one. I suggest that the authors employ the services of a professional English language editor with a background in medical journal articles

We have gone through the whole document and made numerous amendments in this regard. First we made corrections on grammatical errors using “quillbot online grammar checker”. Additionally, the document was revised for language clarity by Dr. Yoseph, a senior English language professional (PhD in Teaching English as a Foreign Language (TEFL)) at University of Gondar, and further corrections were made based on his recommendation. The manuscript has been also revised for academic issues by Dr. Zewdneh Shewamene Sabe (working at Population Council, Ethiopia) and additional corrections were made (eg, deletion of portions in the result and discussion parts). But we are unable to get professional language editor with a background in medical journal articles.

In the Methods section of the Abstract it is stated that data collection was from Jan to Dec 2028, which implies that a convenience sampling was used. However, in the main text under Methods, the authors indicate that sample size determination was used. This is conflicting and needs to be clarified.

Thank you!

First, we determined the number of cases to be included in the study by calculating the sample size as stated in the manuscript (not conveniently determined). Then, we distributed the 442 samples to the four study sites; 111 cases to each of two hospitals and 110 cases to each of the other two hospitals. After this, we started data collection and the period stated is the time required to collect the data in each hospital.

At all sites, the data collection was started on January 1, 2018 and completed in the fourth week of December 2018 on different specific dates. For this reason, we stated that the data collection period ran from January to December of 2018. The time was not predetermined before the data collection. We determined the sample cases to be considered first, distributed them to the four study sites. It took the stated period (January to December 2018) to complete the data collection at each site.

As of the information we got from the emergency departments of the hospitals before starting the data collection, the cases flow was not predictable. Because of this we couldn’t follow a specific sampling technique and rather we decided to consider all consecutive cases till we got the specified number of patients in each hospital. There were few refusal cases and few others with incomplete data and excluded from the study. However, since the data was collected until we achieved the predetermined number of subjects in each study site; the total cases targeted are equal to the originally calculated sample size.

Overall, there are too many tables and figures. Most of the data can be included in 2-3 tables. Also, there is a lot of repetition between the text and figures/tables.

Thank you!

In consideration of this comment, 2 tables and 2 figures that we feel are redundant or do not contain as much relevant information have been removed. The discussion points pertaining to the contents of these tables and figures have also been eliminated. These are the figures and tables that have been removed.

- **Table 2** on Page 7…Variable included in the multivariate analysis
- **Table 4** on page 8…We think the content is not as much relevant.
- **Figure 2** on page 11…The variable included in the multivariate analysis
- **Figure 3** on page 12….Because the content appeared in the multivariate analysis result table.

Table 3: Seems like “Died” and “Cured” under “Treatment outcome” have been incorrectly labelled. The authors are encouraged to read through other similar publications which will assist them with improving the layout and design of this article. The discussion section is too long.

Thank you!

We replaced the term “died” by “cured” and the vice versa.

The discussion part has been reduced from 5 and half pages to about 4 pages.

**Please note that:**

~~Texts with red font color and strikethrough line~~ like this– indicate deletion in the process of revision.

Texts with green highlight – indicate newly added texts in the revision process.
