# Supplementary material for: Poisoning cases and their management in Amhara National Regional State, Ethiopia: Hospital-based prospective study
Source: PLoS One. 2024 May 31;19(5):e0303438. doi: 10.1371/journal.pone.0303438 (PMC11142576; doi:10.1371/journal.pone.0303438)
Supplement: Attachment — Submitted filename: Response to Reviewers.docx [file pone.0303438.s006.docx]

**Response to Review Comments**

Manuscript Title: Poisoning Cases and Their Management in Amhara Region, Ethiopia: Hospital-Based Prospective Study

Manuscript Submission Number: PONE-D-22-26894R3

Journal: PLOS ONE

**Response to the Editor**

Dear Editor,

We extend our heartfelt gratitude to you for handling the publication process of this manuscript. We strongly believe that your skillful management and suggestions are instrumental in ensuring the smooth progress and successful completion of this endeavor.

We have responded to each point raised. The changes made in this revision were implemented after removing all tracked changes in the previous version of the manuscript. Texts that have been newly added or modified during the revision process are indicated with a green highlight, while texts that have been deleted are indicated with a red font color and a strikethrough line. ~~Example~~

Comments from PLOS Editorial Office: We note that one or more reviewers has recommended that you cite specific previously published works. As always, we recommend that you please review and evaluate the requested works to determine whether they are relevant and should be cited. It is not a requirement to cite these works. We appreciate your attention to this request.

One article has been recommended concerning this matter. We deemed the suggested article pertinent and believed it would help consolidate our discussion. Thus, we have cited the paper in the manuscript for its relevance, not merely due to the reviewer's recommendation. It has been cited as reference 23.

Journal Requirements:

Please review your reference list to ensure that it is complete and correct. If you have cited papers that have been retracted, please include the rationale for doing so in the manuscript text, or remove these references and replace them with relevant current references. Any changes to the reference list should be mentioned in the rebuttal letter that accompanies your revised manuscript. If you need to cite a retracted article, indicate the article’s retracted status in the References list and also include a citation and full reference for the retraction notice.

We couldn’t retrieve reference 12 from its online source, and we have replaced it with another recent and relevant reference.

**Response to Reviewer #3**

Dear Reviewer,

We want to express our sincere gratitude for your thorough review of our paper. Your insights and feedback were incredibly valuable to us. Your constructive feedback helped us to see points for improvement and act accordingly. We truly appreciate once again for the time and effort you put into providing such thoughtful feedback. The points raised have been addressed as follows.

Please note that newly added or modified texts during the revision process are indicated with a green highlight, while texts that have been deleted are indicated with a red font color and a strikethrough line. ~~Example~~

Reviewers' comments:
Reviewer's Responses to Questions

**Comments to the Author**

1. If the authors have adequately addressed your comments raised in a previous round of review and you feel that this manuscript is now acceptable for publication, you may indicate that here to bypass the “Comments to the Author” section, enter your conflict of interest statement in the “Confidential to Editor” section, and submit your "Accept" recommendation.

Reviewer #3: (No Response)

2. Is the manuscript technically sound, and do the data support the conclusions?

Reviewer #3: Yes

Thank you!

3. Has the statistical analysis been performed appropriately and rigorously?

Reviewer #3: Yes

Thank you!

4. Have the authors made all data underlying the findings in their manuscript fully available?

Reviewer #3: Yes

Thank you!

5. Is the manuscript presented in an intelligible fashion and written in standard English?

Reviewer #3: No

Thank you!

We have gone over the entire manuscript document again and made some revisions to address some grammatical errors and clarity issues, with emphasis on the suggestions you provided in the 'Review Comments to the Author' section below.

6. Review Comments to the Author

Reviewer #3: There are still some errors in English or confusing expressions, in some places repetition. I have marked up your tracked document in yellow and also suggested strike thru to address this.

Thank you for marking up specific sentences and texts that need more attention for language correction. We have gone through the manuscript and made some modifications based on this suggestion.

In the results you need to detail how many patients were approached and how many refused consent and were subsequently excluded.

Thank you!

A small number of cases declined consent to participate in the study and were consequently excluded. Additionally, some cases from whom sufficient data could not be obtained were excluded from the study. Thus, we have included a description in the first part of the results section, stating how many patients were approached and how many refused consent and were subsequently excluded. We've also provided information on the number of cases that were excluded due to the incomplete data they provided.

Your paper and discussion is really about the epridemioogy of poisoning and some service utilisation. In this context I would avoid to much discussion around the rationale for treatments inevitably it will be a bit superficial.

Thank you!

We acknowledged that it would be appropriate to maintain our primary emphasis on epidemiology and service utilization in discussing the results. We streamlined our discussion, ensuring that the discussion regarding the rationale behind treatments has been limited. However, there are still some points seemingly made about the rationale behind the use of gastric lavage as a GI decontamination method. We were advised by a reviewer during the first round of revision to include these points regarding gastric lavage.

In the discussion the use of exisiting medicines for antidotes is rarely approved it is more commonly recommended in guidelines based on the level of evidence. As this is an epidimology paper you probably do not need to discuss the evidence for the role of magnesium.

For magnesium I have suggested shortening that section. Alos a more approbate reference if needed is

Brvar M, Chan MY, Dawson AH, Ribchester RR, Eddleston M. Magnesium sulfate and calcium channel blocking drugs as antidotes for acute organophosphorus insecticide poisoning–a systematic review and meta-analysis. Clinical Toxicology. 2018 Aug 3;56(8):725-36.

Thank you!

We deleted the section as suggested. Besides, we found the recommended article appropriate and believed it would help consolidate our discussion. Thus, we have cited the paper in the manuscript as per the suggestion (ref. 23).

**Reviewer #5**

We would like to extend our heartfelt appreciation for taking the time to review our work. Your comments have been greatly valuable in improving the manuscript to the highest possible quality. Thank you once again for your highly considerable contribution to the review process of our work.
